# R-catcher, a potent molecular tool to unveil the arginylome

**DOI:** 10.1007/s00018-021-03805-x

**Published:** 2021-03-09

**Authors:** Taewook Seo, Jihyo Kim, Ho-Chul Shin, Jung Gi Kim, Shinyeong Ju, Laxman Nawale, Goeun Han, Hye Seon Lee, Geul Bang, Jin Young Kim, Jeong Kyu Bang, Kyung Ho Lee, Nak-Kyun Soung, Joonsung Hwang, Cheolju Lee, Seung Jun Kim, Bo Yeon Kim, Hyunjoo Cha-Molstad

**Affiliations:** 1 Anticancer Agent Research Center, Korea Research Institute of Bioscience and Biotechnology, Ochang-eup, Cheongju-si, Chungcheongbuk-do 28116 Republic of Korea; 2 Department of Biomolecular Science, KRIBB School, University of Science and Technology, Daejeon, 34113 Republic of Korea; 3 Disease Target Structure Research Center, Korea Research Institute of Bioscience and Biotechnology, Daejeon, 34141 Republic of Korea; 4 Center for Theragnosis, Korea Institute of Science and Technology, Seoul, 02792 Republic of Korea; 5 Research Center for Bioconvergence Analysis, Korea Basic Science Institute, Ochang, 28116 Republic of Korea; 6 Division of Magnetic Resonance, Korea Basic Science Institute, Ochang, 28116 Republic of Korea; 7 KHU-KIST Department of Converging Science and Technology, Kyung Hee University, Seoul, 02447 Republic of Korea

**Keywords:** ATE1 R-transferase, Bait, FBLN1, CLUS, SERPINH1, PRDX4, Unfolded Protein Response, Extracellular exosome, Innate Immune System, Prostate cancer, Ovarian cancer

## Abstract

Protein arginylation is a critical regulator of a variety of biological processes. The ability to uncover the global arginylation pattern and its associated signaling pathways would enable us to identify novel disease targets. Here, we report the development of a tool able to capture the N-terminal arginylome. This tool, termed R-catcher, is based on the ZZ domain of p62, which was previously shown to bind N-terminally arginylated proteins. Mutating the ZZ domain enhanced its binding specificity and affinity for Nt-Arg. R-catcher pulldown coupled to LC–MS/MS led to the identification of 59 known and putative arginylated proteins. Among these were a subgroup of novel ATE1-dependent arginylated ER proteins that are linked to diverse biological pathways, including cellular senescence and vesicle-mediated transport as well as diseases, such as Amyotrophic Lateral Sclerosis and Alzheimer’s disease. This study presents the first molecular tool that allows the unbiased identification of arginylated proteins, thereby unlocking the arginylome and provide a new path to disease biomarker discovery.

**Supplementary Information:**

The online version contains supplementary material available at 10.1007/s00018-021-03805-x.

## Introduction

**Fig. 1:**
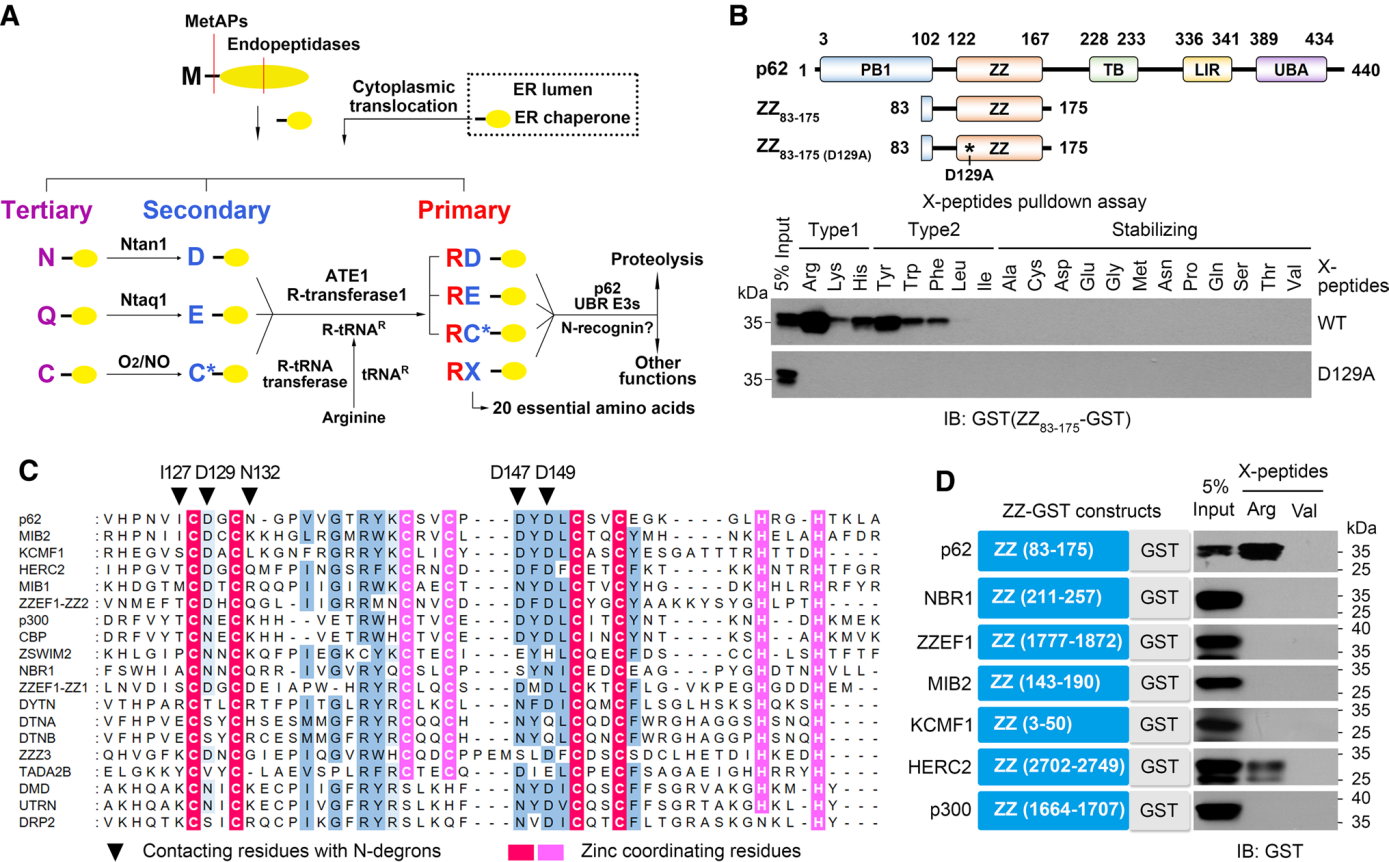
p62-ZZ domain possesses a uniquely high affinity for Nt-Arg. **a** The mammalian Arg/N-degron pathway focusing on protein arginylation. See introduction for a description of the pathway’s mechanistic aspects and biological functions. Neo-N-terminal residues are indicated by single-letter abbreviations for amino acids. Yellow ovals symbolize the remaining portion of a cleaved protein substrate. **b** Schematic drawing of p62 domains as well as wild type and D129A mutant ZZ domain spanning aa 83–175 (upper panel). In vitro peptide pulldown assays to determine binding characteristics of p62-ZZ domain (83–175) to 20 different N-terminal residues of synthetic peptides. N-terminal residues of bead-conjugated peptide are indicated by three-letter abbreviations. Biotinylated 11-mer X-peptides derived from nsP4 N-end rule model substrate were covalently linked to streptavidin agarose beads. Wild type (WT) and mutant (D129A) ZZ_83-175_ tagged with C-terminal GST expressed in HEK293 cells were used as prey. Pulled down ZZ-GST was visualized by western blot analysis using antibody directed against GST. **c** Sequence alignment of 18 ZZ domains present in the human proteome. The black triangles indicate the major residues interacting with the first amino acid of an N-degron. Red and pink columns show zinc-coordinating residues that are composed of two zinc-binding motifs. **d** Assessing binding ability of different ZZ domains to Nt-Arg using in vitro peptide pulldown assays. Blue squares represent ZZ domains derived from 7 different proteins tagged with GST

The N-terminal (Nt) arginylation of proteins is typically mediated by the arginyl-tRNA-protein transferase 1 (ATE1) enzyme encoded by a single arginyltransferase gene that is highly conserved from yeast to human. *ATE1* is essential in mammalian embryogenesis while dispensable in yeast, hinting to acquired ATE1 functions in higher organisms. Mice contain four major ATE1 isoforms that exhibit similar Nt-arginylation specificity and whose expressions are tissue-specific [1, 2]. Without ribosomes, ATE1 can transfer Arg from Arg-tRNA to Nt-Asp, -Glu, and -oxidized Cys residues exposed upon the non-processive proteolytic cleavage of a protein. Regarding the oxidation of Nt-Cys, it has recently been shown to be enzymatically mediated by dioxygenases in plants and humans [3, 4]. Nt-Asp and -Glu residues can also be generated through the deamidation of newly exposed Nt-Asn and -Gln, respectively (Fig. 1a). In addition to ATE1-mediated arginylation, proteolytic cleavage can directly generate neo-Nt-Arg, which results in Arg as the P1’ site, and can be considered as an ATE-independent means of Nt-arginylation. Since proteolytic cleavage by non-possessive proteases including methionine aminopeptidases (MetAP), separases, caspases, calpains produces hundreds of cleaved protein fragments with arginylation-permissive Nt-residues as well as neo-Nt-Arg, Nt-arginylation plays a role in many diverse biological pathways and processes within cells [5, 6].

To date, there are over 30 confirmed mammalian ATE1 substrates that are associated with a broad range of biological functions including cardiovascular development; regulation of G proteins [7, 8]; sensing of heme, nitric oxide, and oxygen [9–12]; inhibition of apoptosis [5]; protein quality control [13]; cell migration [14–16]; gametogenesis and fat metabolism [17]; and neurogenesis and neurodegeneration [18, 19]. Although the arginylation of some proteins, such as β-actin and calreticulin, does not lead to their metabolic instability [14, 20], most arginylated proteins described in these studies have been shown to undergo proteasomal degradation by E3 ubiquitin ligases containing the UBR box motif; hence, Nt-Arg is referred to as a “canonical N-degron” in the N-degron pathway (Fig. 1a).

Our previous studies have revealed that Nt-Arg plays a role in autophagy [21]. We have shown that many ER proteins acquire arginylation-permissive residues upon cleavage of their signal peptides. Under proteotoxic stress, ER chaperones, binding immunoglobulin protein (BiP), calreticulin (CRT), and protein disulfide isomerase (PDI) were found to be relocated into the cytoplasm and arginylated by ATE1. In the cytoplasm, arginylated BiP binds the ZZ domain of p62/sqstm1, an autophagic cargo receptor, via its Nt-Arg. This interaction stimulates autophagy and the autophagic clearance of misfolded proteins, thereby promoting cell survival [21, 22]. Other studies have shown that arginylated CRT is associated with stress granules (SGs) and localized in cell membrane (PM), which regulates the induction of apoptosis [23]. In addition to BiP, CRT, and PDI, more ER proteins have the potential to be arginylated in response to proteotoxic stress, but the identification of these proteins is difficult due to the lack of a molecular tool to visual them.

In 2007, Wong et al. made the first attempt to identify all the arginylated proteins in a cell [24]. This study utilized antibodies raised against peptides containing Arg-Asp- or Arg-Glu- sequences. Therefore, the antibodies were only able to recognize a specific subset of arginylated proteins whose second residue is Asp or Glu. To capture the complete arginylome, multiple antibodies would be needed to capture all possible arginylated proteins. Since it is possible to generate arginylated proteins without ATE1 through endopeptidase cleavage exposing neo-Nt-Arg, the number of antibodies needed would be considerable.

To resolve this issue, we have developed a molecular tool termed R-catcher. R-catcher exploits the ability of the ZZ domain of p62 to bind Nt-arginylated proteins. After defining the minimal ZZ region required for this binding, we further modified the sequence to enhance the binding affinity for Nt-Arg while removing the ability of ZZ to bind other N-degrons. The resulting 54-amino-acid-long polypeptide was linked to the Twin StrepII-His6 tag completing the R-catcher tool. Using HeLa cells treated with MG132 plus thapsigargin (TG), a condition previously seen to induce the arginylation of ER proteins [21], we coupled R-catcher pulldown with LC–MS/MS analysis which led to the identification of 59 known and putative arginylated proteins. As we predicted, novel ATE1-dependent and -independent arginylated ER proteins were identified. Analyzing these proteins and their interactions with other proteins from our pulldown revealed their involvement in many critical biological processes. Therefore, R-catcher represents a valuable tool to unlock the arginylome, which has the potential to reveal novel disease targets.

## Materials and methods

### Antibodies and reagents

The antibodies used in this study are as follows: mouse monoclonal anti-GST (Santa Cruz, sc-138, 1:2000), mouse monoclonal anti-p62 (Abcam, ab56416, 1:100,000), rabbit polyclonal anti-BiP (CST, 3177S, 1:2000), rabbit polyclonal anti-R-BiP (Abfrontier, AR05MA0001, 1:2000), rabbit polyclonal anti-R-CRT (Abfrontier, AR05-PA0002, 1:2000), mouse monoclonal anti-ATE1 (Santa Cruz, sc-271220, 1:1000), mouse monoclonal anti-flag (Sigma, F3165, 1:10,000), mouse monoclonal anti-β-actin (Sigma, A1978, 1:5000), mouse monoclonal anti-Myc (Santa Cruz, sc-40, 1:5000), and mouse monoclonal anti-FK2 specific to Ub-conjugated proteins (ENZO, BML-PW8810-0500, 1:5000). The following secondary antibodies were used: Anti-Mouse IgG (CST, 7074, 1:5000), Anti-Rabbit IgG (CST, 7076, 1:5000).

Formic acid, ammonium bicarbonate, urea, dithiothreitol (DTT), iodoacetamide (IAA), D-biotin (2031), Pierce^™^ GST agarose (20,211), L-Glutathione reduced (G4251), thapsigargin, MG132 and isopropyl-b-D-thio-galactoside (11,411,446,001) were purchased from Sigma-Aldrich (St. Louis, MO, USA). GSTrap 4B (28,401,747), HisTrap HP (17,524,851) and HiLoad ^™^ 25/600 Superdex^™^ 75 pg (28–9893) columns were purchased from GE healthcare. Strep-Tactin XP agarose was purchased from IBA Lifesciences. *E. coli* BL21 (DE3) RIL competent cells were purchased from Novagen. Sequencing-grade-modified trypsin was purchased from Promega (Madison, WI, USA). Streptavidin agarose bead (20,353), Ni–NTA agarose (R90115) and invitrosol solubilizer (MS10007) were purchased from Thermo Fisher scientific (Waltham, MA USA). HPLC-grade acetonitrile was purchased from Burdick and Jackson (Muskegon, MI, USA). Water was purified using a Milli Q system (Millipore, Molsheim, France). RA (4,017,629.1000) and AR (4,005,990.011) dipeptides were purchased from BACHEM. Silver staining kit (EBP-1051) was purchased from ELPISBIO (Deajeon, Korea).

### Plasmid construction and mutagenesis

ZZ domains of p62, NBR1, ZZEF1, MIB2, KCMF1, HERC2 and p300 were sub-cloned into pcDNA3.1-GST (To generate pcDNA3.1-GST, GST encoding DNA was inserted into pcDNA3.1(+)/myc/His A using EcoRI and XhoI sites) using either BamHI-EcoRI (NBRI-ZZ, ZZEF1-ZZ, KCMF1-ZZ and p300-ZZ) sites or HindIII-EcoRI (MIB2-ZZ and HERC2-ZZ) sites. pGST-N3 was previously constructed by switching EGFP of pEGFP-N3 vector to GST using BamHIII/NotI sites. To generate N- and C-terminally deleted constructs of p62-ZZ (83–175), PCR amplified DNAs were inserted into pGST-N3 vector (Clone Tech.) using HindIII/BamHI sites. Point mutations, such as N132D or N132Q of ZZ (122–175), were introduced by performing two-step PCR using primers containing appropriate mutations. MX-RGS4 (X = Cys or Val), Ub-X-BiP (X = Arg or Val), Ub-X-PDI (X = Arg or Val) were sub-cloned into pcDNA3.1( +)/myc/His A using EcoRI/XhoI sites. For bacterial expression and purification, ZZ_122-175_ N132D-amplified DNA was sub-cloned into pGEX6p-1 vector using EcoRI/XhoI sites. R-catcher was constructed by sub-cloning PCR amplicon encompassing Twin strepII-His6-ZZ_122-175_^N132D^ into pETDuet-1 (Novagen) vector using NdeI/XhoI sites. For the confirmation of novel arginylated ER proteins, PCR amplified cDNAs (FBLN1, FKBP10, SERPINH1, PRDX4, ENPL, CLUS and LG3BP) were inserted into pcDNA3.1(+)/myc/His B using KpnI/XhoI (PRDX4, CLUS and LG3BP) or EcoRI/NotI (SERPH1, FKB10, ENPL and FBLN1) sites. The point mutation at the P1’ site of SERPINH1, PRDX4, CLUS, and FBLN1 was introduced by performing two-step PCR using primers containing appropriate mutations.

### Cell culture and immunoblotting

HEK293 and HeLa (CCL2, ATCC) cells as well as + / + and *ATE1*^*−/−*^ MEFs were cultured in DMEM (Hyclone) supplemented with 10% FBS (Gibco) in 5% CO_2_ incubator. ATE knockout MEFs were verified using immunoblotting analysis. We regularly test cell lines for mycoplasma contamination. For immunoblotting, cells were lysed directly in 2 × SDS sample buffer, then separated by SDS-PAGE and transferred onto PVDF membrane (Bio-Rad). For pulldown assays, cells were lysed in hypotonic buffer (20 mM HEPES pH7.9, 10 mM KCl, 1.5 mM MgCl2) with protease inhibitor cocktail tablet (Roche).

### Purification of GST-ZZ recombinant proteins and R-catcher

To purify GST-p62-ZZ recombinant proteins, plasmid DNA encoding either wild type or D129A mutant GST-ZZ^N132D^ was transformed into BL21 DE3 (Novagen). Cells were grown overnight in Luria–Bertani broth (LB broth) at 37 °C, 200 rpm until it reached O.D600 0.6 at which 1 mM isopropyl-β-D-thio-galactoside (IPTG) was added. The *E. coli* culture was transferred to 18 °C incubator and grown for another 16 h at 200 rpm. Cells were collected by centrifugation at 6,790 × *g* for 20 min and lysed in buffer A (50 mM Tris–HCl pH 8.0, 300 mM NaCl, 100 µM PMSF, 10% glycerol) with gentle sonication followed by centrifugation at 26,000 × *g* for 30 min. The supernatant was transferred to a fresh tube and filtrated with 0.45 µM pore syringe membrane filter. The filtered supernatant was loaded on GSTrap 4B (GE healthcare) column which was connected with AKTA FPLC (GE Healthcare) at a flow rate of 1 mL/min and washed with 10 CV (column volume) of lysis buffer (50 mM Tris–HCl pH 8.0, 300 mM NaCl, 100 µM PMSF, 10% glycerol) at the same flow rate when real time chromatogram reached the basal level. Bound proteins were eluted using lysis buffer containing 20 mM reduced glutathione.

For isothermal titration calorimetry (ITC), GST-fused p62 (122–175) wild type and mutants (N132Q and N132D) were expressed in *E. coli* BL21 (DE3) RIL competent cells in Luria–Bertani media with 0.1 mM Isopropyl-β-D-thio-galactoside (IPTG) at 18 °C. Cells were collected and sonicated in a B buffer (50 mM Tris–HCl pH 7.5, 200 mM NaCl) containing 3 mM β-mercaptoethanol (β-ME). Cell lysates were centrifuged at 18,000 × *g* for 1 h to remove cell debris, and this supernatant was loaded onto Glutathione Sepharose^™^ 4 Fast Flow resin and washed with B buffer. GST-fused proteins were eluted with 20 mM glutathione in an elution buffer (100 mM Tris–HCl pH 7.5, 200 mM NaCl, and 3 mM β-ME). The eluted samples were dialyzed in a dialysis buffer (50 mM Tris–HCl pH 7.5, 50 mM NaCl, and 3 mM β-ME) with or without TEV protease. These samples were loaded onto anion exchange column (HiTrap Q HP, GE Healthcare) and unbounded samples were collected and loaded onto size exclusion chromatography (HiLoad ^™^ 25/600 Superdex^™^ 75 pg column, GE healthcare) with the B buffer containing 0.2 mM Tris(2-carboxyethyl)phosphine (TCEP).

Plasmid DNA encoding R-catcher (pETDuet1-twin strepII-His6-ZZ_122-175_ N132D) was transformed into BL21 DE3. Cells were cultured in 1L LB media containing 0.5 mM isopropyl-b-D-thio-galactoside (IPTG) at 18 °C overnight and pelleted by centrifugation at 6790 × *g*. The cell pellet was re-suspended and lysed in lysis buffer (50 mM Tris–HCl pH 8.0, 300 mM NaCl, 100 nM PMSF, 10% glycerol) with gentle sonication. The cell lysates were centrifuged at 26,000 × *g* for 30 min to remove cell debris and then filtrated using 0.45 µm pore syringe membrane. The filtered supernatant was loaded on HisTrap HP (5 ml) column connected with AKTA FPLC (GE Healthcare) at a flow rate of 1 ml/min. Bound proteins were eluted using lysis buffer containing 250 mM imidazole with concentration gradient after washing.

### X-peptide and R-catcher pulldown assays

Plasmids encoding p62-ZZ-GST were transiently transfected into HEK293 cells following Xtreme Gene HP transfection protocol. After 24 h, cells were washed with PBS and harvested by scraping. Cell pellets were re-suspended in hypotonic buffer (5 volume of cell pellet), incubated on ice for 30 min and subjected to five times of freeze–thaw cycles, followed by 15,928 × *g* at 4 °C for 20 min. For X-peptide pull-down assays, 200 µg total protein or 1 µg purified GST-ZZ was incubated with biotinylated X-nsP4 11-mer peptides (X = Arg, His, Lys, Tyr, Trp, Phe, Leu, Ile, Ala, Cys, Asp, Glu, Gly, Met, Asn, Pro, Gln, Ser, Thr or Val) conjugated on streptavidin agarose resin in binding buffer (20 mM HEPES pH7.9, 0.2 M KCl, 0.05% Tween 20, 10% glycerol) with gentle rotation for 3 h at 4 °C.

For small-scale R-catcher pull-down assays, 300 µg of purified R-catcher was conjugated with 120 µl of Strep-Tactin sepharose resin by incubating overnight at 4 °C. To test R-catcher’s ability to capture known arginylated proteins, such as R-BiP, R-PDI, R-nsP4 and R-RGS4, plasmids encoding Ub-X-(BiP, PDI and nsP4) or MX-RGS4 (X = Arg or Val) were transiently transfected into HEK293 cells. After 24 h, cells were treated with 10 µM MG132 and 100 nM thapsigargin for another 24 h. Cells were harvested and lysed in hypotonic buffer and 300 µg total protein was incubate with 25 µl R-catcher-sepharose resin in binding buffer (20 mM HEPES pH 7.9, 0.05% Tween 20, 10% glycerol, 0.2 M KCl, protease inhibitor and phosphatase inhibitor) for 3 h with gentle rotation at 4 °C. After three washes with binding buffer, bound proteins were dissociated with 2 × SDS sample buffer and boiling at 100 °C and subjected to immunoblotting using anti-myc or flag antibodies.

For large-scale R-catcher pulldown assays, HeLa cells were treated with 5 µM MG132 and 100 nM thapsigargin for 24 h. Cells were lysed in hypotonic buffer and 10 mg total was incubated with 750 µl R-catcher-sepharose beads for 3 h at 4 °C after pre-clearing with D129A mutant R-catcher-sepharose beads for 3 h at 4 °C. After three washes with binding buffer, bound proteins were eluted with binding buffer containing 20 mM D-biotin. To remove R-catcher, the eluent was dialyzed using 20 kDa cut-off dialysis membrane in binding buffer containing 3.5 M MgCl_2_ overnight followed by another overnight dialysis in distilled-deionized water. The dialyzed eluent was lyophilized overnight and dried powder was dissolved in 100 µl LC/MS solubilizer for silver staining and mass spectrometry analysis.

### Isothermal titration calorimetry (ITC)

Purified proteins were concentrated to 0.05 mM. 4-mer peptides as injectant were resolved in B buffer to a final concentration of 1 mM. ITC measurement was performed at 25 °C on a Microcal Auto-ITC200.

## Liquid chromatography and mass spectrometry/mass spectrometry

### Protein digestion and solubilization

Protein samples solubilized in Invitrosol LC/MS solubilizer (Invitrogen) as following by manufacturer protocol. For removal of incompatible buffer components, protein samples filtrate with 10 K MW ultrafilter for complete digestion. For preparing trypsinization of samples, the retentates were dissolved in 50 mM of ammonium bicarbonate (pH 8.0) untill it reach 1 μg/μL of final concentration. The samples treated with 10 mM DTT at 60 °C for 45 min. Following 20 mM IAA addition, the final mixture incubated in dark for 45 min at 25 °C. 1:50 ratio of Trypsin was added on mixture. The mixture incubated overnight at 37 °C. Final digested samples dried with speed-vacuum. Samples re-dissolved in 20 μL of water containing 0.1% formic acid for LC–MS/MS analysis.

### LC–MS/MS analysis

Peptides were analyzed using a LC–MS/MS system consisting of an Easy nLC 1000 (Thermo Fisher scientific) and an Orbitrap Fusion Lumos mass spectrometer (Thermo Fisher Scientific) equipped with a nano-electrospray source (EASY-Spray Sources, Thermo Fisher Scientific). Peptides were trapped 75 μm × 2 cm C18 pre-column (nanoViper, Acclaim PepMap100, Thermo Fisher Scientific) before being separated on an analytical C18 column (75 μm × 50 cm PepMap RSLC, Thermo Fisher Scientific) at a flow rate of 300 nL/min. The mobile phases A and B were composed of 0 and 100% acetonitrile containing 0.1% formic acid, respectively. The LC gradient began with 5% B and was ramped to 8% B for 1 min, 10% B for 16 min, to 40% B for 79 min, to 80% B for 1 min, and remained at 80% B over 8 min. Finally, it was ramped to 5% B for another 5 min. The column was re-equilibrated with 2% B for 10 min before the next run. The voltage applied to produce an electrospray was 1900 V. During the chromatographic separation, the Orbitrap Fusion Lumos was operated in data-dependent mode, automatically switching between MS1 and MS2. The MS data were acquired using the following parameters: Full-scan MS1 spectra (400–1600 m/z) were acquired in the Orbitrap for a maximum ion injection time of 100 ms at a resolution of 120,000 and an automatic gain control (AGC) target value of 4.0e5. MS2 spectra were acquired in the Orbitrap mass analyzer at resolution of 30,000 with high-energy collision dissociation (HCD) of 27% normalized collision energy and AGC target value of 5.0e4 with maximum ion injection time of 54 ms. Previously fragmented ions were excluded for 30 s.

### Protein identification and quantification

MS/MS spectra were analyzed using the following software analysis protocols with the Uniprot human DB (2019). The reversed sequences of all proteins were appended into the database for calculation of false discovery rate (FDR). ProLucid [25] was used to identify the peptides, a precursor mass error of 5 ppm, and a fragment ion mass error of 200 ppm. Trypsin was selected as the enzyme, with one potential missed cleavage. Carbamidomethylation at cystein was chosen as static modifications. Oxidation at methionine was chosen as variable modification. The output data files were filtered and sorted to compose the protein list using the DTASelect [26] (The Scripps Research Institute, USA) with two and more peptides assignments for a protein identification and a false positive rate less than 0.01.

### Identification of arginylation sites

Upon separation of the immunoprecipitated protein by SDS-PAGE and staining with Coomassie Blue, the corresponding protein band was excised, half of which was subjected to GelNrich and the other half to conventional in-gel digestion GelNrich is an N-terminal peptide enrichment method based on in-gel digestion. The method includes in-gel protein level labeling of primary amines using d6-acetic anhydride and post-digestion negative selection of labeled N-terminal peptide(s) using N-hydroxysuccinimide activated agarose beads. Peptides recovered from GelNrich or in-gel digestion were dried in a vacuum evaporator and reconstituted in 0.1% formic acid. An aliquot was injected from a cooled (10 °C) auto-sampler into a reversed-phase EASY-Spray column (25 cm × 75 μm, Thermo Fisher Scientific) on an Eksigent nanoLC-ultra 1D plus system (Danaher Corporation) at a flow rate of 300 nL/min. Prior to use, the column was equilibrated with 96% buffer A (0.1% formic acid in water) and 4% buffer B (0.1% formic acid in acetonitrile). The peptides were eluted with a linear gradient from 4 to 35% buffer B over 60 min and 35–80% buffer B over 2 min followed by an organic wash and aqueous re-equilibration at a flow rate of 300 nL/min with a total run time of 96 min. The HPLC system was coupled to a Q-Exactive mass spectrometer (Thermo Fisher Scientific) operated in a data-dependent acquisition (DDA) mode. Survey full-scan MS spectra (m/z 400–1800) were acquired with a resolution of 70,000. The MS/MS spectra of the 12 most intense ions from the MS1 scan with charge states 1 ~ 5 were acquired with the following options: resolution, 17,500; automatic gain control (AGC) target, 5E4; isolation width, 2.0 m/z; normalized collision energy, 27%; dynamic exclusion duration, 60 s; and ion selection threshold, 4.2E2 counts. Source ionization parameters were as follow: spray voltage, 1.9 kV; capillary temperature, 275 °C; and s-lens level, 50.0. The RAW-files from the Q-Exactive were directly matched to the peptide sequence in the Uniprot human database (release 2020. 2) using a Sequest HT node of Proteome Discoverer (v2.4). The search engine settings for GelNrich data were as follows: Arg-C enzyme specificity with 1 as the number of tolerable termini; 20 ppm for MS1 tolerance; variable modification: oxidation of methionine (+ 15.9949 Da); fixed modifications: carbamidomethyl of cysteine (+ 57.021464 Da) and D_3_-acetyl of lysine; N-term variable modifications: Glu- > pyro-Glu (-18.0106 Da), acetyl (+ 42.0106 Da), D_3_-acetyl (45.029395 Da) and D_3_-acetyl-arginyl (+ 201.130506 Da). The settings for in-gel digestion data were as follows: trypsin enzyme specificity with 1 as the number of tolerable termini; 20 ppm for MS1 tolerance; variable modification: oxidation of methionine (+ 15.9949 Da); fixed modification: carbamidomethyl of cysteine (+ 57.021464 Da); N-term variable modifications: Glu- > pyro-Glu (− 18.0106 Da), acetyl (+ 42.0106 Da) and arginyl (156.101111 Da). Search outputs from Sequest HT were conveyed to Percolator. The false discovery rate was set to 1% at the PSM level. For annotating peptide spectra, we used a freely accessible version of The Interactive Peptide Spectrum Annotator.

### Bioinformatic data analysis

GeneAnalytics free bioinformatic online program in Gene cards was used to analyze Molecular Function, Biological Processes, Cellular Comopartment, Biological Pathway and Diseases associated with the arginylated ER protein networks.

### Statistical analysis

Data are presented as mean ± SD of more than three independent experiments. Statistical analysis was performed with Prism 6 software (Graph Pad) using ANOVA. Differences with *P* < 0.05 were considered statistically significant [***P < 0.001; n.s., no statistically significant difference (*P* ≥ 0.05)].

## Results

### The ZZ domain of p62 has the highest binding affinity for Nt-Arg than any other ZZ domain in the human proteome

We have previously shown that the ZZ domain fragment (ZZ_83-175_), aa 83 to aa 175 of p62, can bind basic Nt residues, Arg, Lys and His (type-1 N-degrons) and bulky aromatic residues, Tyr, Trp and Phe (type-2 N-degrons), but not stabilizing Nt-Val. Among these, p62 exhibited the highest affinity for Nt-Arg [22]. Since other Nt residues, such as Pro and Gly, can act as degrons in certain contexts, we decided to analyze the binding of ZZ_83-175_ with all 20 possible Nt amino acid residues using in vitro peptide pulldown assays [27, 28]. In these assays, we used HEK293 cell lysates expressing ZZ_83-175_-GST and biotinylated rotavirus non-structural protein 4 (nsP4) peptides, a model N-end rule substrate. As expected, ZZ_83-175_ had a high binding affinity for Nt-Arg, and moderate affinity for Nt-Lys, -His, -Tyr, -Phe, and -Trp (Fig. 1b). However, ZZ_83-175_ was unable to bind any of the other typically stabilizing residues. As previously shown with full-length p62, the alanine substitution of Asp129 (D129A) within ZZ_83-175_ abolished its ability to bind all N-degrons [22].

To determine whether other ZZ domains in the human proteome can bind Nt-Arg, we searched the UniProt database. Approximately 18 proteins (Supplementary Table 1) in the human proteome were found to contain a ZZ-type zinc finger domain similar to the p62-ZZ domain. These domains possess 4 to 6 Cys residues plus additional His residues that can coordinate two zinc ions. The alignment of the 18 ZZ domains revealed that the zinc-coordinating residues are highly conserved (Fig. 1c). According to the X-ray crystal structure of the p62-ZZ domain [29], four Cys residues (Cys128, C131, Cys151, and Cys154) coordinate one zinc ion, and two Cys residues (Cys142 and Cys145) and two His residues (His160 and His163) coordinate the other zinc ion. In addition, a negatively charged patch composed of Asp129, Asp147, and Asp149 is conserved in many of the ZZ domains. Notably, Asn132 is only present in the ZZ domain of p62 (Fig. 1c), and the bipolar nature of this residue has been shown to be responsible for the interaction of the p62-ZZ domain with both type-1 and type-2 N-degrons [29, 30]. We sub-cloned the ZZ domains of mind bomb homolog 2 (MIB2), potassium channel modulatory factor 1 (KCMF1), HECT domain and RCC1-like domain containing protein 2 (HERC2), zinc finger ZZ-type and EF-hand domain containing protein1 (ZZEF1), histone acetyltransferase p300 (p300), and next to BRCA1 gene 1 protein (NBR1), which showed the highest similarity to the p62-ZZ domain, and evaluated their ability to bind Nt-Arg using in vitro peptide pulldown assays. Interestingly, only the p62-ZZ domain had a high affinity for Nt-Arg, while other ZZ domains showed no or minimal binding to Nt-Arg (Fig. 1d). Therefore, we chose to use the p62-ZZ domain as the basis for our tool.

### ZZ_122-175_ retains a high binding affinity for Nt-Arg

**Fig. 2:**
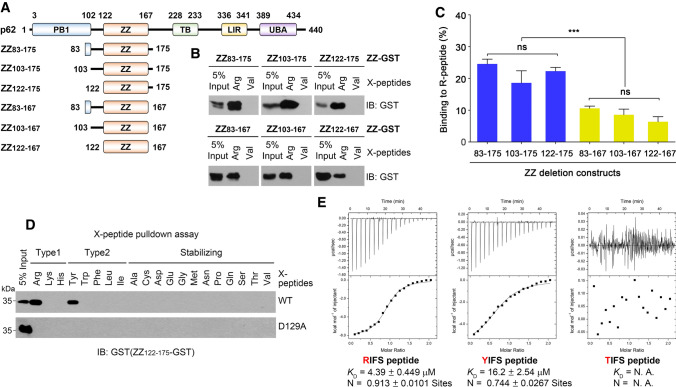
Minimized p62 ZZ_122-175_ retained its ability to bind only Nt-Arg and Nt-Tyr. **a** A schematic drawing describing serial deletions of p62 ZZ_83-175_. Each deleted ZZ fragment was linked to C-terminal GST tag. **b** X-peptide pulldown assays to assess the ability of each ZZ fragment to bind Nt-Arg. Bound ZZ-GST was detected by western blot analysis using anti-GST antibody. **c** represents western blot quantification of **b** by image J. The graph represents the average percent binding of ZZ-GST to R-nsP4 peptide. The intensity of ZZ-GST band was compared to 5% input. Data represent the mean (± S.D.) of three independent experiments. Statistical significance was calculated using a two-way ANOVA test (*P ≤ 0.05; ***P < 0.001; ****P < 0.0001; n.s., not significant). **d** X-peptide pulldown assays to examine GST-ZZ_122-175_ fragment’s binding characteristics to 20 different N-terminal residues. D129A mutant was used as a negative control. **e** Isothermal titration calorimetry to measure the binding affinities of GST-ZZ_122-175_ domain for 4-mer peptides; RIFS, YIFS, and TIFS. The upper panel of each graph shows the heat changes with each injection as a function of time. The lower panel shows a plot of the calculated enthalpies per injection versus molar ratio of ligand and target (squares). Also shown is the result of fitting the data to a single site-binding model using the MicroCal origin software package

To construct our tool, we decided to minimize ZZ_83-175_ since it contains flanking regions outside of the established ZZ domain (residues #122–167) (Fig. 2a). To determine if these flanking regions are required for p62 to optimally bind Nt-Arg, we generated N-terminally and C-terminally deleted constructs of ZZ_83-175_ tagged with C-terminal GST and tested their ability to bind Nt-Arg using in vitro peptide pulldown assays. The results showed that deleting residues 83 to 122 did not significantly affect Nt-Arg binding; however, the deletion of residues 167 to 175 substantially reduced Nt-Arg binding (Fig. 2b, c). Based on the results, we selected ZZ_122-175_ as a minimal unit that retains the desired binding affinity for Nt-Arg. Using ZZ_122-175_, we next tested its ability to bind all 20 possible Nt residues using biotinylated peptides and HEK293 cell lysates expressing the ZZ_122-175_-GST construct. The results showed that ZZ_122-175_ retained its high binding affinity for Nt-Arg but lost its ability to bind all other Nt residues except Nt-Tyr (Supplementary Fig. 1A). At this point, we also examined the UBR box of human UBR1, which is a predecessor of p62-ZZ and had been shown to bind Nt-Arg. We found that the UBR box (aa 97 to aa 167) could bind Nt-Arg and Nt-His while it did not bind other N-degrons (Supplementary Fig. 1c). However, we decided to continue to develop p62-ZZ instead of the UBR box because its published *K*_D_ for Nt-Arg was low (24 µM), it is larger (17 amino acid longer) than ZZ_122-175_. Next, we purified bacterially expressed GST-ZZ_122-175_ using glutathione resin (Supplementary Fig. 1B). Using this purified protein, the peptide pulldown assays were repeated with similar results (Fig. 2d). Again, the D129A point mutation abolished the binding of all Nt residues. Our isothermal titration calorimetry (ITC) analysis revealed that GST-ZZ_122-175_ binds the RIFS tetrapeptide (Nt-Arg) with a *K*_D_ of 4.4 µM and the YIFS tetrapeptide (Nt-Tyr) with a *K*_D_ of 16.15 µM (Fig. 2e).

### ZZ_122-175_^N123D^ binds exclusively to Nt-Arg at a higher affinity than ZZ_122-175_

**Fig. 3:**
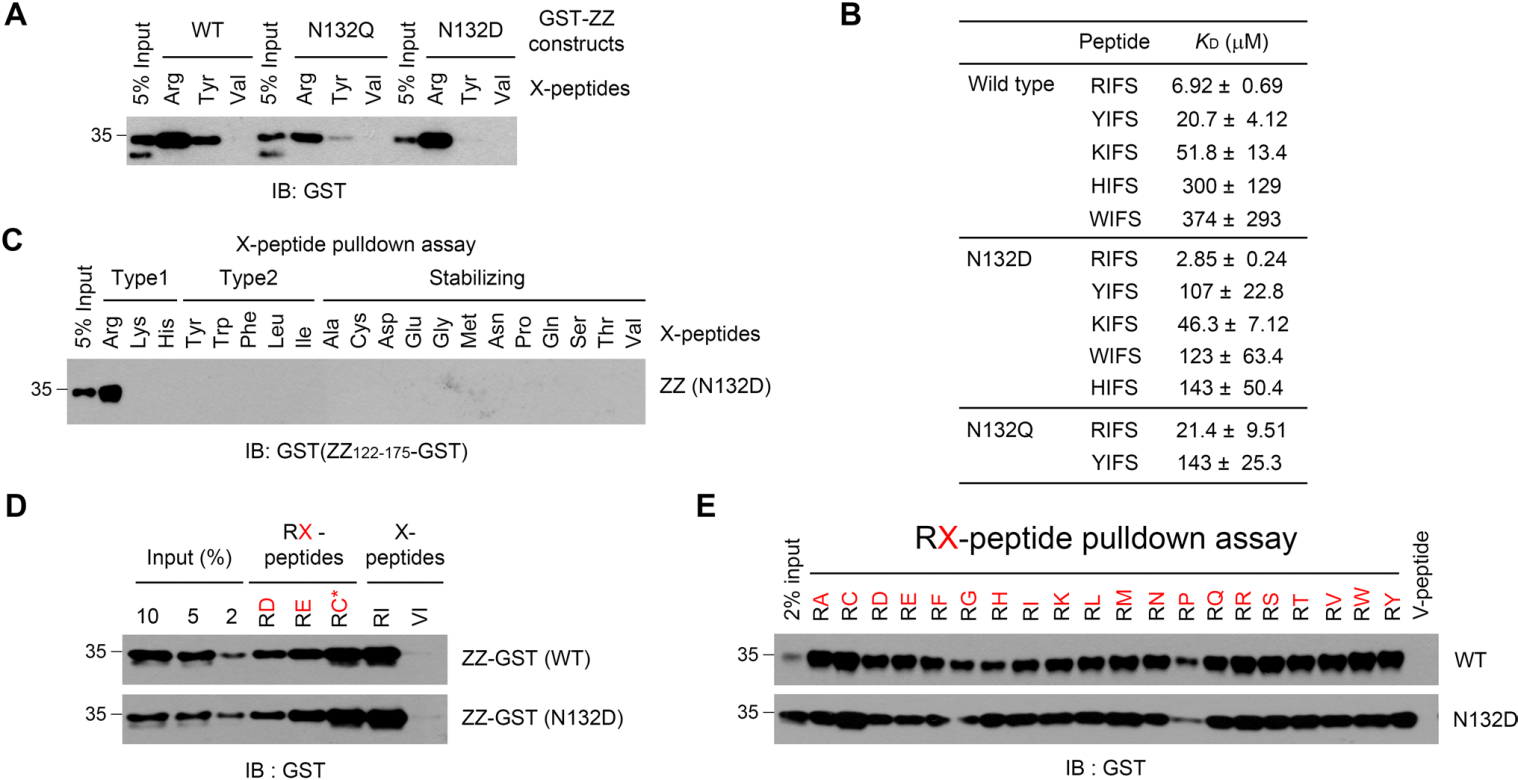
N132D mutation enhanced the binding affinity and specificity of ZZ_122-175_ for Nt-Arg. **a** Mutational analysis of ZZ_122-175_ to increase its binding specificity for Nt-Arg. Wild type, N132Q and N132D ZZ-GST transiently expressed in HEK293 cells were tested for their binding specificity for Nt-Arg using in vitro X-peptide pulldown assays (X = Nt-Arg, Nt-Tyr or Nt-Val). Bound ZZ-GST was detected by immunoblotting using anti-GST antibody. **b** ITC results showing the binding affinity (*K*_D_) of purified wild type, N132D and N132Q ZZ fragments for 4-mer peptides containing N-degrons (RIFS, YIFS, KIFS, HIFS and WIFS). Calculated dissociation constants (*K*_D_) are shown on the table. **c** X-peptide pulldown assays using purified ZZ_122-175_^N123D^-GST to examine its binding of 20 different N-terminal amino acid residues. **d** RX-peptide pulldown assays to assess the effect of the 2nd arginylation accepting residues, Asp, Glu and oxidized Cys on the binding of affinity-purified GST tagged wild type and N132D mutant ZZ to the first Arg residue. **e** RX-peptide pulldown assays to examine the effect of 20 different 2nd residues (X) on the binding of affinity-purified GST tagged wild type and N132D mutant ZZ to the 1st Arg residue

To generate a tool that binds explicitly to Nt-Arg, we mutated ZZ_122-175_ to reduce its affinity for Nt-Tyr. To accomplish this, we focused on the ZZ residue Asn132 since its bipolar nature is critical for the p62-ZZ domain to bind both basic and aromatic N-degrons (Supplementary Fig. 2). We substituted this residue with either glutamine (N132Q), a methylene group longer version of asparagine, or aspartic acid (N132D), a polar version of asparagine. These mutants and the wild type version of ZZ_122-175_ were expressed in HEK293 cells and tested for their ability to bind Nt-Arg or Nt-Tyr using in vitro peptide pulldown assays. The results showed that compared to wild type, the N132Q mutation decreased the affinity of ZZ_122-175_ for N-degrons in general, whereas the N132D mutation not only increased the affinity for Nt-Arg but also significantly lowered the affinity for Nt-Tyr (Fig. 3a). Our ITC analysis verified these results in that the N132D mutant had a binding affinity of 2.85 µM for the RIFS tetrapeptide, which is over two-fold higher than wild type (6.9 µM). On the other hand, the N132D mutant had a binding affinity of 107 µM for the YIFS tetrapeptide, which is approximately five times lower than the wild type (20.7 µM) (Fig. 3b and Supplementary Fig. 1A–C). We again confirmed that compared to the wild type and the N132D mutant, the UBR box had lower affinity for Nt-Arg (Supplementary Fig. 1E). Consistently, our in vitro peptide pulldown assays using biotinylated X-peptides containing 20 different Nt residues demonstrated that ZZ_122-175_ containing the N132D mutation (ZZ_122-175_^N132D^) only bound Nt-Arg and lost its ability to bind Nt-Tyr (Fig. 3c).

### ZZ_122-175_^N132D^ is capable of binding any Nt-arginylated protein, regardless of its 2nd residue

The 2nd residue of an Nt-arginylated protein can affect the binding affinity of Nt-Arg to the UBR box [31]. To determine whether this is the case with the ZZ domain, affinity-purified GST- ZZ_122-175_ and GST- ZZ_122-175_^N132D^ (Supplementary Fig. 3D) were used to test the effect of arginylation-permissive residues Asp, Glu, and oxidized Cys (2nd residue of an ATE1-dependent arginylated protein) on the binding of Nt-Arg using in vitro peptide pulldown assays. The results showed that both the wild type and the N132D mutant could bind all ATE1-dependent arginylated proteins (Fig. 3d). Since Nt-arginylated proteins can be generated in an ATE1-independent manner through proteolytic cleavage, we again performed peptide pulldown assays using biotinylated peptides representing all possible 2nd residues. The results demonstrated that both ZZ_122-175_ and ZZ_122-175_^N132D^ could bind all Nt-arginylated proteins regardless of the 2nd residue (Fig. 3e) while the UBR box’s affinity was affected by the 2nd residue (Supplementary Fig. 3F). All together, we concluded that ZZ_122-175_^N132D^ is the best basis for creating a bait to comprehensively capture Nt-arginylated proteins in vivo.

### R-catcher can capture known cellular Nt-arginylated proteins induced under proteotoxic stress

**Fig. 4:**
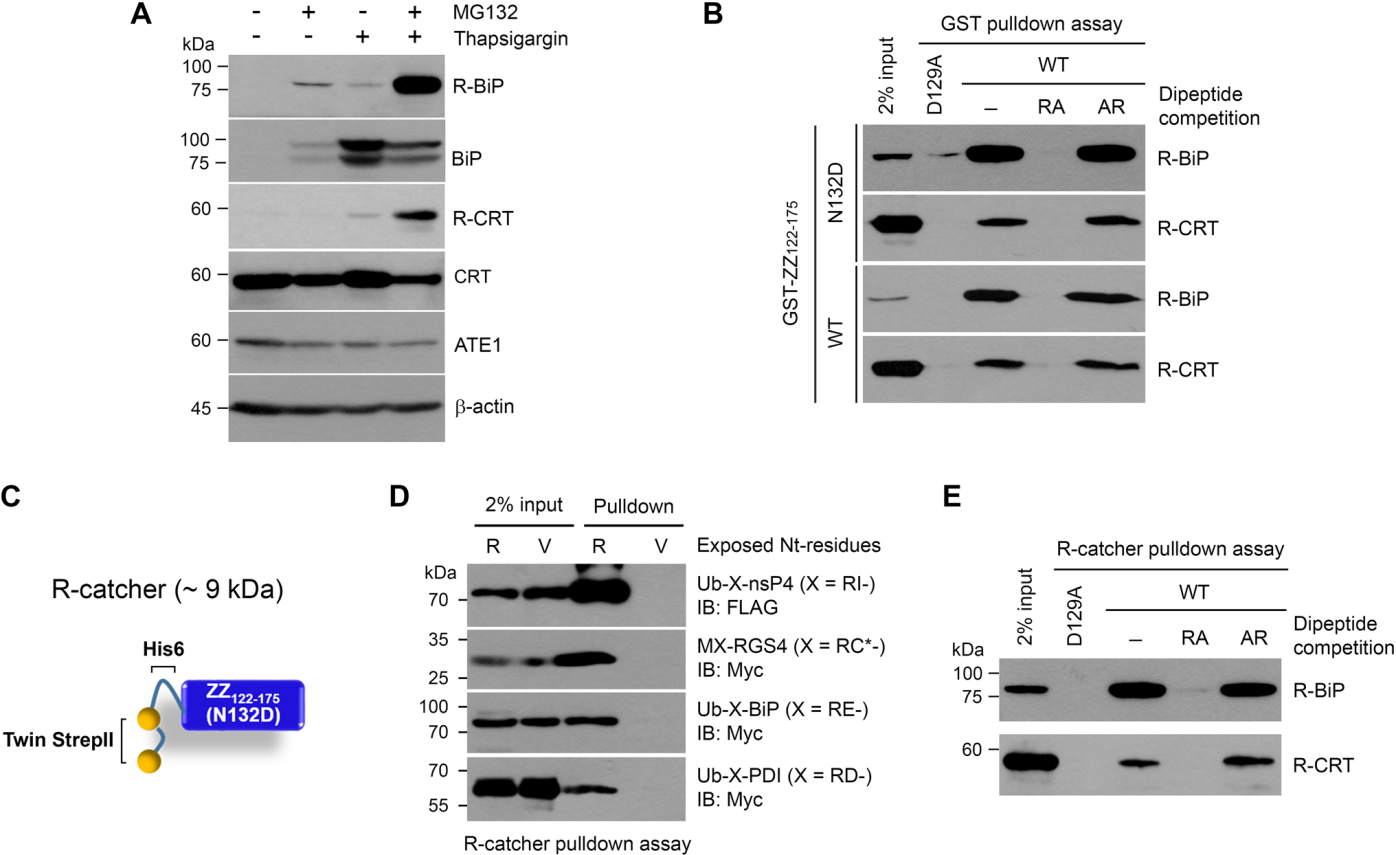
ZZ_122-175_^N132D^ is capable of capturing known arginylated proteins in cells. **a** Western blot analysis to visualize arginylated BiP and CRT. To induce the arginylation of BiP and CRT, HeLa cells were stimulated with 10 µM MG132 plus 100 nM thapsigargin (TG) for 24 h. N-terminally arginylated BiP and CRT were detected by immunoblotting using anti R-BiP and R-CRT antibodies. **b** GST pulldown assays to test the ability of wild type (WT) and N132D GST-ZZ in capturing cellular R-BiP and R-CRT. HeLa cell lysates stimulated with MG132 plus TG were incubated with purified GST-ZZ (WT & N132D) immobilized on GSH beads in the absence and presence of 25 mM dipeptides. D129A mutant and competition with RA dipeptide were used to inhibit ZZ and Nt-Arg interaction. Pulled down R-BiP and R-CRT were detected using anti R-BiP and R-CRT antibodies. **c** Schematic drawing illustrating R-catcher. R-catcher is composed of ZZ_122-175_^N132D^ tagged with twin strepII-His6 at the N-terminus, which generates approximately a 9 kDa protein. **d** R-catcher pulldown assays to test its ability to capture a variety of known arginylated proteins. ATE1 model substrates (Ub-X-nsP4, MX-RGS4, Ub-X-BiP and Ub-X-PDI) were transiently overexpressed in HEK293 cells and the lysates were incubated with R-catcher immobilized on tactin beads. R-catcher bound arginylated proteins were detected by immunoblotting using anti-Myc or Flag antibody. **e** R-catcher pulldown assay to capture R-BiP and R-CRT induced in HeLa cells stimulated with MG132 plus TG in the absence or presence of 20 mM dipeptides

To test whether ZZ_122-175_^N132D^ can capture known arginylated proteins in vivo, we stimulated HeLa cells with the proteasome inhibitor MG132 plus the ER stress inducer thapsigargin (TG) which has been shown to induce the Nt-arginylation of ER chaperones, BiP and CRT (Fig. 4A). Using these lysates and purified GST tagged ZZ_122-175_^N132D^ (Supplementary Fig. 4A) immobilized on glutathione resin, we performed pulldown assays. The results showed that ZZ_122-175_^N132D^ could efficiently pull down Nt-arginylated BiP and CRT regardless of the presence of AR dipeptides, whereas these interactions were inhibited by the D129A mutation and RA dipeptide competition (Fig. 4b). In addition to pulling down R-BiP and R-CRT, GST-ZZ_122-175_^N132D^ was able to capture polyubiquitinated proteins, which were also disrupted by the D129A mutation and RA dipeptide competition (Supplementary Fig. 4B). This was expected because arginylated proteins are known to be ubiquitinated by E3 ligases containing a UBR box leading to their proteasomal degradation.

Due to technical issues related to mass spectrometry (MS), we switched the GST tag previously used with a smaller Twin StrepII-His6 tag to generate an approximately 9 kDa protein (Twin StrepII-His6-ZZ_122-175_^N132D^), termed “R-catcher” (Fig. 4c). To ensure this new tag did not affect the binding of R-catcher to Nt-Arg, we used purified R-catcher (Supplementary Fig. 4C) to demonstrate that R-catcher could pulldown arginylated proteins containing various 2nd residues; nsP4 (2nd Ile), RGS4 (2nd oxidized Cys), BiP (2nd Glu) and PDI (2nd Asp) (Fig. 4d). We also confirmed that R-catcher could pulldown endogenous arginylated BiP and CRT, and these interactions were also abolished by the D129A mutation and RA dipeptide competition (Fig. 4e). Overall, these results suggest that R-catcher would make an ideal bait to capture any arginylated proteins in vivo.

### R-catcher captured 59 known and putative Nt-arginylated proteins

**Fig. 5:**
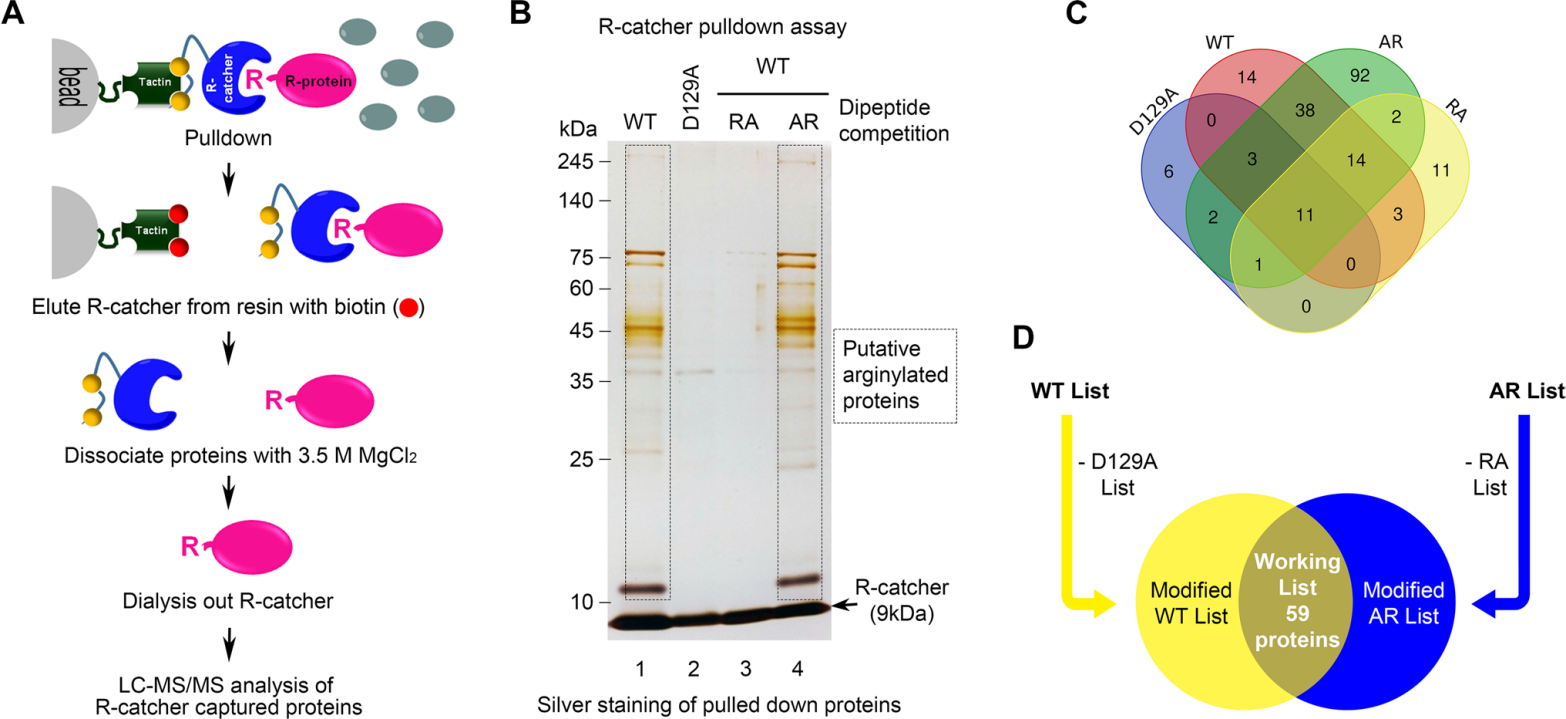
Proteomic identification of R-catcher captured proteins. **a** Schematic representation illustrating R-catcher pulldown coupled to LC–MS/MS. **b** Silver staining of R-catcher captured proteins. To perform large-scale R-catcher pulldown assay, HeLa cell lysates prepared after MG132 plus TG stimulation were precleared with beads conjugated with D129A mutant R-catcher for 3 h. 10 mg precleared cell lysates were incubated with R-catcher in the absence or presence of 25 mM dipeptides (RA or AR) for 3 h at 4 °C. After washing beads, bound proteins were eluted with 10 mM biotin and dialyzed using 20 kDa MW cut-off membranes to remove biotin, R-catcher and MgCl_2_. Dialyzed samples were lyophilized overnight followed by solubilization with 100 µl invitrosol LC/MS solubilizer. LC–MS/MS analysis was performed to identify R-catcher captured proteins. **c** Venn diagram showing analyzed MS data using Calculate and Draw Custom Venn diagrams in Bioinformatics and Evolutionary Genomics website. Venn diagram was generated based on the sum of peptide intensity. **d** Schematic diagram summarizing the selection protocol for the working list

In an attempt to comprehensively identify arginylated proteins in vivo, we performed large-scale R-catcher pulldown assays using HeLa cell extracts stimulated with MG132 plus TG (Fig. 5a). Small aliquots of pulldown samples were separated on a 4 to 20% gradient gel, and protein bands were visualized by silver staining (Fig. 5b). Proteins captured by wild type R-catcher (WT) without and with AR dipeptide competition (AR) revealed a similar band pattern whose molecular weight ranged from 10 kDa to over 245 kDa, but the majority of the proteins were concentrated between 25 and 70 kDa (Fig. 5b lane 1 and 4). Both the D129A mutation (D129A) and RA dipeptide competition (RA) abolished the majority of the proteins (Fig. 5b ane 2 and 3). To identify proteins captured by R-catcher, liquid chromatography mass spectrometry (LC–MS/MS) was performed, and the results are summarized in Fig. 5c. Identified R-catcher binding proteins were analyzed based on total peptide intensities. We generated four protein lists based on the pulldown conditions: WT, AR, D129A, and RA. As shown in the Venn diagram (Fig. 5c, Supplementary Tables 2–5), the WT (83) and AR (163) protein lists contain about four times more proteins than the D129A (23) and RA (42) protein lists, which is consistent with the silver staining results (Fig. 5b). To generate our final working list, we first removed any proteins from the WT list that were in the D129A list except for the proteins whose total peptide intensity was at least threefold greater in the WT list. We repeated the process using the AR and RA list. Then, we only selected proteins found in both the modified WT list and the modified AR list to obtain a final working list of 59 proteins (Fig. 5d, Supplementary Table 6). An examination of the list revealed that approximately 10% of the proteins had been previously reported as arginylated proteins [24]. The R-catcher pulldown was done under proteotoxic stress conditions (MG132 + TG), a condition known to lead to the arginylation of ER chaperones BiP and CRT. As expected, both BiP and CRT were among the known arginylated proteins. These results confirm that R-catcher can capture known arginylated proteins.

### R-catcher captured the ER arginylome induced by proteotoxic stress

**Fig. 6:**
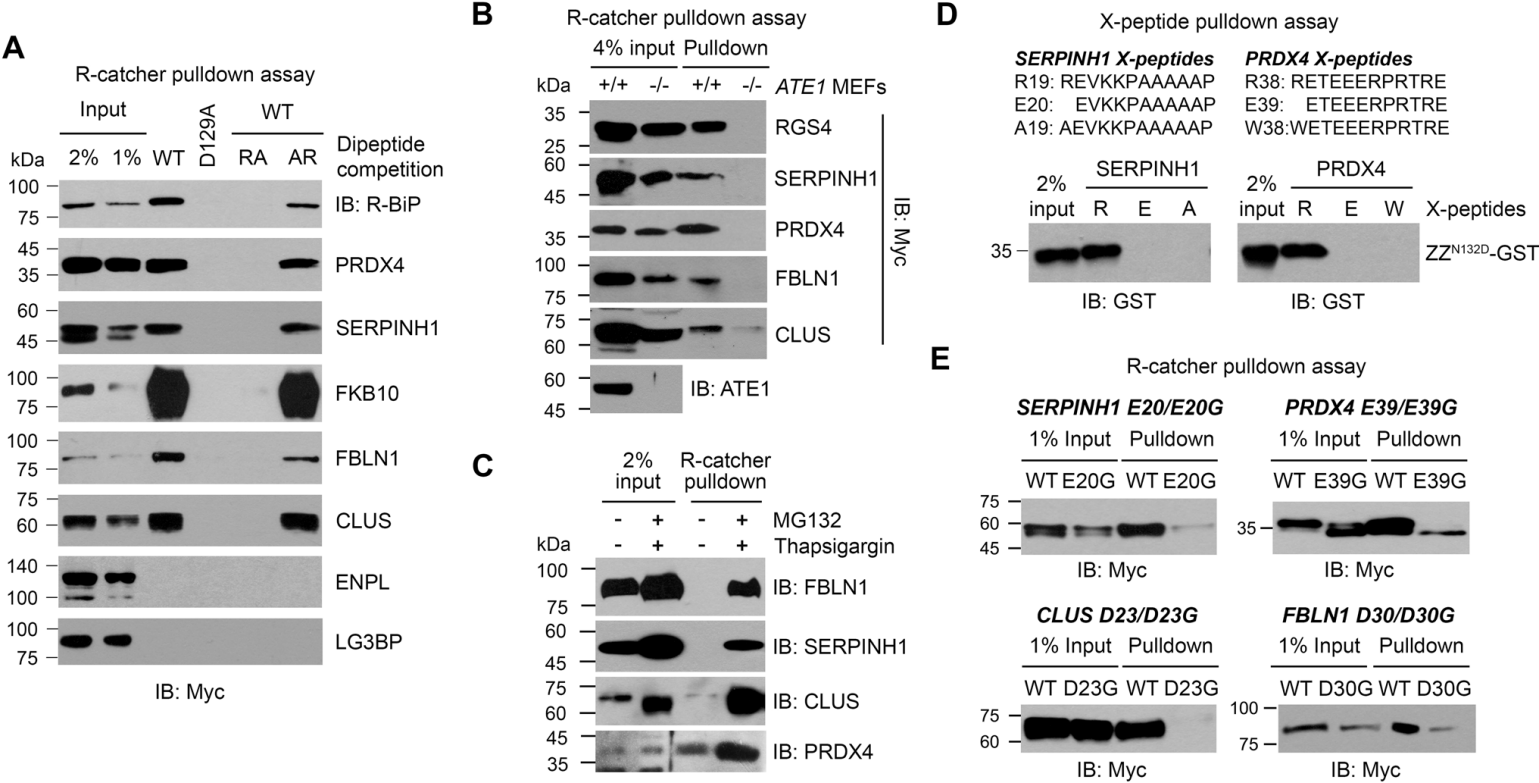
R-catcher revealed novel arginylated ER proteins. **a** R-catcher pulldown assays to validate the N-end rule dependent binding of putative arginylated ER proteins with R-catcher. Plasmids encoding the proteins were transiently transfected to HeLa cells for 24 h and stimulated with 5 µM MG132 plus 100 nM TG for another 24 h. Cell lysates were incubated with R-catcher immobilized on tactin beads for 3 h. After wash, bound proteins were eluted with 2 × SDS sample buffer followed by boiling at 100 °C for 10 min and visualized by western blot analysis. **b** R-catcher pulldown assays to validate ATE1-dependent arginylation of SERPHINH1, PRDX4, FBLN1 and CLUS. Plasmids encoding SERPHINH1, PRDX4, FBLN1 and CLUS were transiently transfected into + / + and *ATE1*^−/−^ MEFs for 24 h and stimulated with 5 µM MG132 plus 100 nM TG for another 9 h. Cell lysates were subjected to R-catcher pulldown, and bound proteins were detected by western blot analysis. **c** R-catcher pull-down assays to evaluate if the arginylation of FBNL1, SERPINH1, CLUS and PRDX4 is induced in response to MG132 plus thapsigargin. **d** X-peptide pulldown assays to confirm no interaction of R-catcher with Nt-Ala19 of SERPINH1 peptides and Nt-Trp38 of PRDX4 peptides. X-peptides where X is A19 (SignalP predicted P1’ site), E20 (next possible P1’ site predicted by PrediSi) or R19 (Arginylated form of Glu) for SERPINH1 and W38 (SignalP predicted P1’ site), E39 (next possible P1’ site predicted by PrediSi) or R38 (Arginylated form) for PRDX4. Biotinylated X-peptides immobilized on streptavidin agarose beads were incubated with affinity-purified GST-ZZ (N123D). Bound GST-ZZ (N132D) was detected by immunoblotting. **e** Identification of arginylated sites employing mutational analysis. Predicted P1’ sites of SERPINH1, PRDX4, CLUS and FBLN1 were substituted to Gly, and the inhibitory effect of the mutation on binding with R-catcher was examined by performing R-catcher pulldown assays

Our previous bioinformatic analysis of the ER proteome revealed that approximately 43 ER residents and clients acquire arginylation-permissive Nt residues (Asp, Glu, and oxidized Cys) as well as direct Nt-Arg after cleavage of their signal peptides [21]. This led us to speculate that many of these proteins may be arginylated under the same condition as BiP and CRT. In fact, our working list contains 10 ER-associated proteins comprising 2 known (BiP and CRT) and 8 putative arginylated proteins (Table 1). We selected these proteins for further analysis. To examine the Nt-Arg-dependent binding of these proteins to R-catcher, we constructed plasmids encoding these proteins tagged with myc/His. HeLa cells transiently expressing each protein were treated with MG132 plus TG to induce their retro-translocation into the cytoplasm, which would lead to ATE1-dependent arginylation if they possess arginylation-permissive Nt residues. Using the HeLa cell lysates, R-catcher pulldown assays were performed as previously described in Fig. 4e. The results showed that, as expected, endogenous R-BiP bound R-catcher in an Nt-Arg-dependent manner as seen by RA competition (Fig. 6a). Peroxiredoxin-4 (PRDX4), serpin H1 (SERPINH1), clusterin (CLU), fibulin-1 (FBLN1), and FK506-binding protein 10 (FKBP10) also showed Nt-Arg-dependent binding with R-catcher while endoplasmin (ENPL) and lectin galactoside-binding soluble 3-binding protein (LG3BP) showed no significant binding affinity for R-catcher suggesting that their presence in the working list may be through the interaction of another arginylated intermediate. FKBP10 exhibited an unusually high binding affinity for R-catcher likely due to directly having a neo-Nt-Arg as its P1’ site after signal peptide cleavage (Table 1). Next, we determined if R-catcher binding with SERPINH1, PRDX4, FBLN1, and CLUS is dependent on ATE1. To address this, we transiently transfected + / + and *ATE1-/-* MEFs with plasmids encoding these proteins and stimulated the cells with MG132 plus TG followed by R-catcher pulldown assays. The results revealed that all the proteins were captured by R-catcher in an ATE1-dependent manner (Fig. 6b). In addition, we found that the arginylation of these ER proteins appears to be inducible since their binding with R-catcher was strongly induced by MG132 plus thapsigargin (Fig. 6c).

**Table 1:** Arginylated ER protein candidates

Uniprot ID	Gene symbol	Name	Predicted P1’ site (signalP)		Location
P27797	CALR	Calreticulin	MLLSVPLLLGLLGLAVA**E**PAVYFKEOFLOGOGWTSRWIES	E18	Er
P50454	SERPINH1	Serpin H1	MRSLLLLSAFCLLEAALA**A**EVKKPAAAAAPGTAEKLSPKA	A19	Er
P10909	CLU	Clusterin	MMKTLLLFVGLLLTWESGOVLG**D**QTVSONELOEMSNOGSK	D23	Er
096AY3	FKBP10	Peptidyl-prolyl cis-trans isomerase FKBP10	MFPAGPPSHSLLRLPLLOLLLLVVOAVG**R**GLGRASPAGGP	R29	Er
013162	PROX4	Peroxiredoxin-4	MEALPLLAATTPOHGRHRRLLLLPLLLFLLPAGAVOG**W**ET	W38	Er
P14625	HSP90B1	Endoplasmin	MRALWVLGLCCVLLTFGSVRA**D**DEVOVOGTVEEOLGKSRE	D22	Er
P11021	HSPA5	Endoplasmic reticulum chaperone BiP	MKLSLVAAMLLLLSAARA**E**EEOKKEOVGTVVGIOLGTTYS	E19	Er
P81605	OCO	Oermicidin	MRFMTLLFLTALAGALVCA**Y**DPEAASAPGSGNPCHEASAA	Y20	Secreted
008380	LGALS3BP	Galectin-3-binding protein	MTPPRLFWVWLLVAGTOG**V**NOGOMRLAOGGATNOGRVEIF	V19	Secreted
P23142	FBLN1	Fibulin-1	MERAAPSRRVPLPLLLLGGLALLAAGVOA**D**VLLEACCAOG	D30	Secreted

10 ER proteins are listed. SignalP was used to predict P1’ sites. The predicted P1’ site is shown in bold red letters

The predicted P1’ sites for FBLN1 and CLUS are D30 and D23, respectively, both arginylation-permissive sites, explaining their ATE1-dependent arginylation (Table 1). However, the predicted P1’ sites for SERPINH1 and PRDX4 are A19 and W38, respectively, which are not arginylation-permissive (Table 1). In both proteins, the residue after the predicted cleavage site is Glu, which is arginylation-permissive. The PrediSi prediction software showed that this residue is the second most likely P1’ site. As expected, R-catcher bound neither Nt-Ala19 of SERPINH1 nor Nt-Trp38 of PRDX4 while it could bind these proteins when Nt-arginylated at the Glu residue (Fig. 6d). To pinpoint the site of arginylation for these proteins, we substituted their predicted P1’ sites: E20 for SERPINH1, E30 for PRDX4, D23 for CLUS and D30 for FBLN1, to Gly. We confirmed that these point mutations did not affect the predicted cleavage of their signal peptides using both SignalP and PrediSi (data not shown). Our R-catcher pulldown results demonstrated that Gly substitution of their P1’ sites significantly impaired their interaction with R-catcher (Fig. 6e). To further verify the arginylation of these ER proteins, we performed GelNrich [32] and in-gel digestion LC–MS/MS [33] analysis of the immunoprecipitated ER proteins. Consistent with our data shown in Fig. 6e, peptide spectra of CLUS and FBLN1 indicated the arginylation at the D23 residue and the D30 residue, respectively (Supplementary Fig. 5). Unfortunately, the peptide spectra of SERPINH1 and PRDX4 arginylation were not detected, possibly because the length of the peptides (4 amino acid long peptide for SERPINH1 and 9 amino acid long peptide for PRDX4) generated by trypsin digestion is too short to be readily identified by our MS methods. Further investigation is needed to further verify their arginylation. These results indicated that, at minimum, CLUS and FBLN1 are novel substrates of ATE1 and it is likely that SERPINH1 and PRDX4 are also, but further conformation is required.

### The arginylated ER protein network is linked to major biological pathways and diseases, such as Amyotrophic Lateral Sclerosis (ALS), and various cancers

**Fig. 7:**
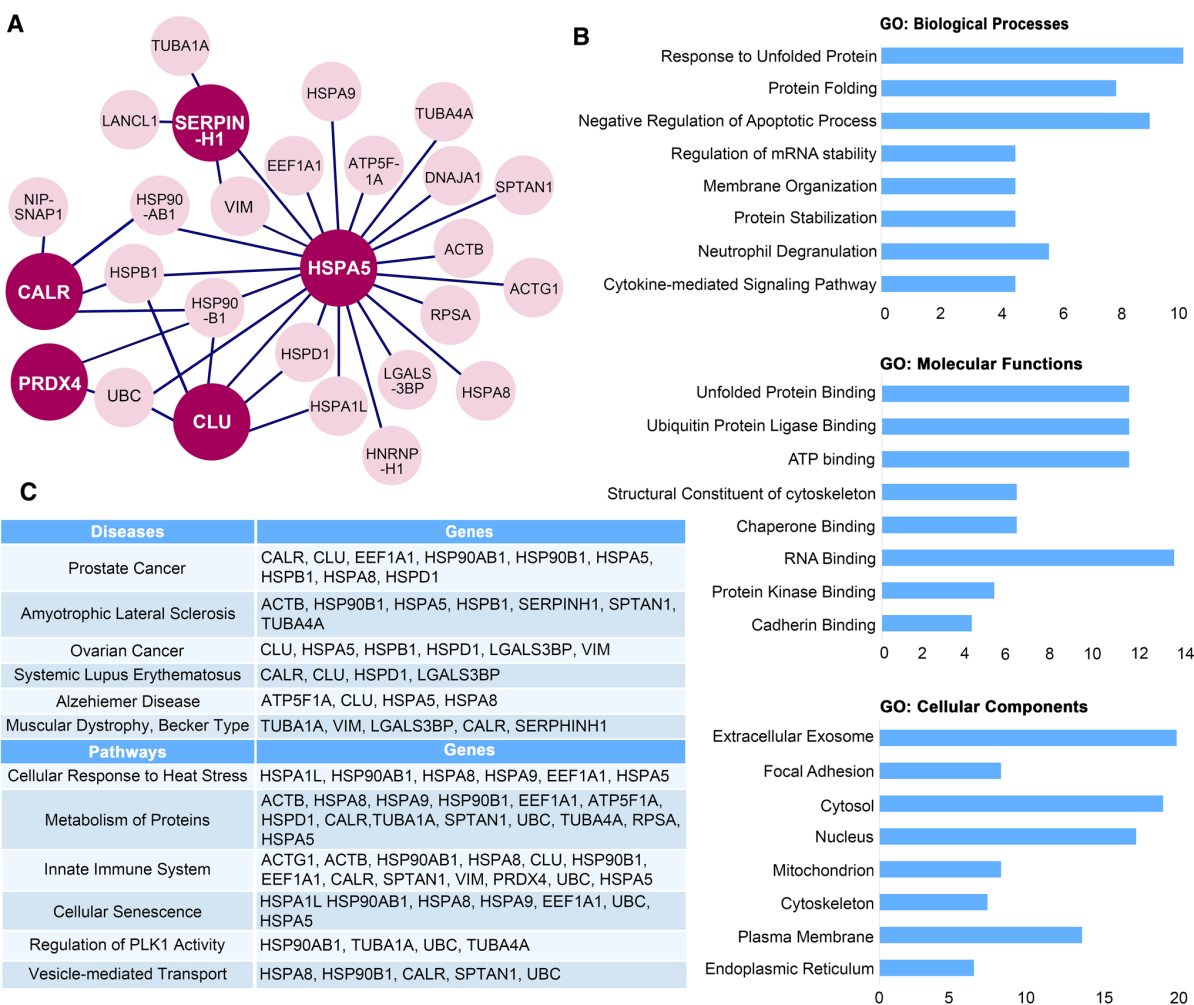
Bioinformatic analysis of arginylated ER proteins. **a** Interaction networks of arginylated ER proteins using BioGRID. Arginylated ER proteins from Table 1 are shown in wine color circles. Their interactors are shown in pink circles. Interactors are from our working list, and the interactions were confirmed using BioGRID. **b** Gene ontology analysis of arginylated ER proteins and their interactors using GeneAnalytics tool. Biological processes, molecular functions and cellular components that are higher than medium grade based on GeneAnalytics are selected. Medium grade means *P* < 0.05 and high grade means *P* < 0.0001 (Suppl. Figure 6). **c** Summary of biological pathways and diseases associated with the arginylated ER protein network obtained using GeneAnalytics tool. Biological pathways and diseases that are higher than medium grade based on GeneAnalytics are selected. Grades of biological pathways and diseases are shown in Suppl. Figure 6

Our previous study demonstrated that luminal BiP retrotranslocates into the cytoplasm, where it is arginylated by ATE1 in response to proteotoxic stress. Nt-arginylated BiP (R-BiP) acquires novel cytoplasmic functions, one of which is mediated by the interaction between its Nt-Arg and the ZZ domain of p62, an autophagic cargo receptor, which leads to p62 oligomerization and autophagic stimulation [21]. To obtain insight into the potential roles of these new ATE1 ER substrates, we examined their interaction with other proteins in our working list using BioGrid data. This inquiry led to the identification of a network of proteins centering around these ER ATE1 substrates (Fig. 7a). Analysis of this protein cluster using the GeneAnalytics tool [34] revealed that these proteins are not only involved in expected processes, such as the unfolded protein response, but also in the regulation of mRNA stability (Fig. 7b). In addition, most of these proteins can be located in extracellular exosomes. Further analysis using the GeneAnalytics tool showed that these proteins are implicated in major cellular pathways, such as the innate immune system, the regulation of PLK1 activity and vesicle-mediated transport (Fig. 7c). Also, they are involved in diseases including prostate cancer and ALS. Using R-catcher would allow us to study the role of arginylation in these diseases and may lead to the identification of novel therapeutic targets.

## Discussion

Protein Nt-arginylation is a post-translational modification required for many critical biological processes. The enzyme mediating this process has been identified as arginyl-tRNA-protein transferase (ATE1) whose substrates are proteins with N-terminal Asp, Glu, and oxidized Cys residue (Fig. 1a). The generation of these residues is dependent on their cleavage by non-processive proteases, including MetAPs, calpains, caspases, and separases (Fig. 1a). Since these proteases are activated in a spatio-temporal manner in response to a variety of cellular cues (stresses) and usually cleaving hundreds of cellular proteins exposing neo-N-termini including arginylation-permissive ones, protein arginylation can occur to a group of proteins in a context-dependent manner. In addition, subcellular compartments, such as the ER lumen and mitochondrial matrix, contain a milieu of neo-N-termini, including arginylation-permissive Nt residues, which may translocate into the cytoplasm where they can be Nt-arginylated by ATE1 upon specific cellular processes or stresses. A prominent example comes from our previous study in which we demonstrated the ATE1-mediated arginylation of ER chaperones, Glu19-BiP, Glu18-CRT, and Asp18-PDI, which were retro-translocated into the cytoplasm under proteotoxic stress using antibodies specific for Nt-arginylated BiP, CRT, and PDI. In addition to these ER chaperones, many other ER-targeted proteins are predicted to be arginylated under this condition according to our bioinformatic analysis [21]. A lack of a molecular tool to comprehensively identify arginylated proteins in cells has been an obstacle to understanding such global and dynamic roles of protein arginylation in response to specific cellular cues.

In this study, we have developed a powerful molecular tool, R-catcher, allowing the unbiased identification and study of cellular arginylated proteins. The tool was built around our discovery that the ZZ domain of p62 binds Nt-arginylated proteins. We first examined whether other ZZ domains in the human proteome are also able to bind Nt-Arg. Our analysis of the six most closely related ZZ domains revealed that only the p62-ZZ domain has a significant affinity for Nt-Arg, showing that subtle differences in biochemical features can severely affect function. In addition to Nt-Arg, the p62-ZZ domain can bind Nt-Lys, -His, -Tyr, -Phe, and -Trp. Through a deletion series, we confirmed that the ZZ domain (residues#122–175) can bind Nt-Arg (*K*_D_ = 4.4 µM) and Nt-Tyr (*K*_D_ = 16.15 µM). The affinity of ZZ_122-175_ for Nt-Arg is substantially higher than the UBR boxes of UBR1 and UBR2, whose *K*_D_ is 24 µM and 19.3 µM, respectively, suggesting that the p62-ZZ domain is also superior to the UBR boxes in binding Nt-Arg [35]. Furthermore, the single N132D mutation not only removed the ability of the ZZ domain to bind Nt-Tyr but also enhanced its affinity for Nt-Arg (*K*_D_ = 2.85 µM) because of the negative charge of Asp. Adding the small Twin StrepII-His6 tag to the construct completed the R-catcher tool. We coupled R-catcher pulldown with LC–MS/MS analysis and identified 59 known and putative arginylated proteins in response to proteotoxic stress using stringent criteria. Six of these proteins were previously reported arginylated proteins, including the expected ER proteins, BiP and CRT. However, the PDI protein was not detected in our list. This could be due to its low binding affinity for R-catcher (Fig. 4d); thus, it may have been masked by other protein signals in the MS/MS spectrum. We are currently working on methods to enhance the N-termini of proteins after R-catcher pulldown. The preliminary result of these experiments has detected R-PDI binding to R-catcher. These results show that R-catcher functioned as expected. Thus, providing a means to capture Nt-arginylated proteins.

An examination of the arginylated proteins captured by R-catcher identified a subset of proteins belonging to the ER–Golgi Secretory pathway. Analysis of these proteins led to the discovery of four putative novel ATE1 substrates, SERPINH1, FBLN1, CLUS, and PRDX4. Since our method has possibility to pulldown non-arginylated proteins co-purified with arginylated proteins, we first attempted to identify their arginylation sites by introducing point mutation (Gly) on their predicted P1’ site, which abolished their binding with R-catcher (Fig. 6e). To further verify their arginylation, we performed mass spec analysis (GelNrich and in-gel digestion LC–MS/MS) and demonstrated that FBLN1 and CLUS are arginylated on the same residues (D30 and D23, respectively) identified using mutagenesis studies shown in Fig. 6e. However, our MS approaches failed to detect the arginylation of SERPINH1 and PRDX4. One likely explanation is that the resulting peptides (SERPINH1: Arg-Glu-Val-Lys, PRDX4: Arg-Glu-Thr-Glu-Glu-Glu-Arg-Pro-Arg) generated by trypsin digestion are too short for us to readily identify their arginylation. Therefore, the arginylation of these proteins warrants further investigation.

Looking further into the biological functions of the novel ATE1 substrates, *CLU* has caught our attention because it is the most cited gene among the four and has been identified as the third greatest late onset Alzheimer’s disease (LOAD) risk gene following *APOE* and *BIN1*. CLUS, a normally secreted protein, has been found intracellularly under certain stress conditions, and its biological roles have been implicated in lipid transport and immune modulation as well as its prominent extracellular chaperone function [36]. It is also involved in cell death and survival, oxidative stress, and proteotoxic stress. Although the upregulation of CLUS levels in hippocampus, cortex, and cerebrospinal fluid of AD brain suggests it as a promising biomarker, CLUS’s mechanism of action in the pathogenesis of AD has not been elucidated [37]. In this regard, it will be very interesting to see if the major species of intracellular CLUS is the arginylated form, and if the arginylated CLUS would play a key role in the pathogenesis of AD and neurodegeneration [38, 39]. Like CLUS, FBLN1 is also a secreted protein. It is not only known as a key regulator of cell adhesion and motility but also involved in cellular transformation and tumor invasion [40–43]. FBLN1 gene point mutation is associated with FBLN1-related developmental delay-central nervous system anomaly-syndactyly syndrome which is characterized by delayed motor development, intellectual disability, dysarthria, pseudobulbar signs, cryptorchidism, and syndactyly. SERPINH1 is an endoplasmic reticulum (ER)-resident chaperon that plays a critical role in proper folding of procollagen in mammalian cells [44]. A missense mutation in SERPINH1 has been shown to cause severe recessive osteogenesis imperfecta which is a connective tissue disorder characterized by low bone mass, bone fragility and susceptibility to fractures after minimal injury [45]. Peroxiredoxin-4 is a thiol-specific peroxidase that catalyzes the reduction of hydrogen and organic hydroperoxides to water and alcohols, respectively. It regulates the activation of NF-kB by modulating IkB alpha phosphorylation. Recent studies have shown that it plays a key role in cancer progression across different types of cancer [46].

Our interactome analysis of the arginylated ER proteins captured by R-catcher with the other members of the pulldown list revealed a distinct protein network. Bioinformatic analysis of this network using the GeneAnalytics tool showed that these proteins are involved in biological processes, such as the regulation of apoptosis and mRNA stability, and associated with various cellular compartments, especially extracellular exosome. In addition, this network is linked to several super pathways, such as the regulation of PLK1, cellular senescence and vesicle-mediated transport. These proteins are also associated with major neurodegenerative diseases, such as ALS and Alzheimer’s disease, which may be related to the previously reported p62 mutation, D129N, found in patients with neurodegenerative diseases [47]. The role of arginylated form of these ER proteins in the cytoplasm warrants further investigations in the context of neurodegeneration. Taken together, R-catcher represents a valuable molecular tool to unlock the arginylome, which has the potential to reveal novel disease targets.

## Supplementary Information

Below is the link to the electronic supplementary material.

Supplementary file1 (PDF 1491 KB)
