# Supplementary material for: R-catcher, a potent molecular tool to unveil the arginylome
Source: Cell Mol Life Sci. 2021 Mar 9;78(7):3725–41. doi: 10.1007/s00018-021-03805-x (PMC8038991; doi:10.1007/s00018-021-03805-x)
Supplement: Supplementary file 1 — Supplementary file1 (PDF 1491 KB) [file 18_2021_3805_MOESM1_ESM.pdf]

Supplementary information for

R-catcher, a Potent Molecular Tool to Unveil the Arginylome

Taewook Seo[1, 2], Ji Hyo Kim[1], Ho-Chul Shin[3], Jung Gi Kim[1, 2], Sin Young Ju[4, 7], Laxman Nawale[1, 2], Goeun Han[1, 2], Hye Seon Lee[3], Geul Bang[5], Jin Young Kim[5], Jeong Kyu Bang[6], Kyung Ho Lee[1], Nak Kyun Soung[1], Jun Sung Hwang[1], Cheolju Lee[4, 7], Seung Jun Kim[2, 3]*, Bo Yeon Kim[1, 2]*, Hyunjoo Cha-Molstad[1, 2]*

1 Anticancer agent research center, Korea Research Institute of Bioscience and Biotechnology, Ochang 28116, Republic of Korea.

2 Department of Biomolecular Science, University of Science and Technology, Daejeon 34113, Republic of Korea.

3 Disease Target Structure Research Center, Korea Research Institute of Bioscience and Biotechnology, Daejeon 34141, Republic of Korea.

4 Center for Theragnosis, Korea Institute of Science and Technology, Seoul 02792, Republic of Korea.

5 Division of Mass Spectrometry Research, Korea Basic Science Institute, Ochang 28116, Republic of Korea.

6 Division of Magnetic Resonance, Korea Basic Science Institute, Ochang 28116, Republic of Korea.

7. KHU-KIST Department of Converging Science and Technology, Kyung Hee University, 02447, Seoul, Republic of Korea

***Corresponding**

*Hyunjoo Cha-Molstad: hcha@kribb.re.kr. ,*Bo Yeon Kim : bykim@kribb.re.kr. , *Seung Jun Kim: ksj@kribb.re.kr.

Supplementary figure 1-6

and

Supplementary Table 1-6

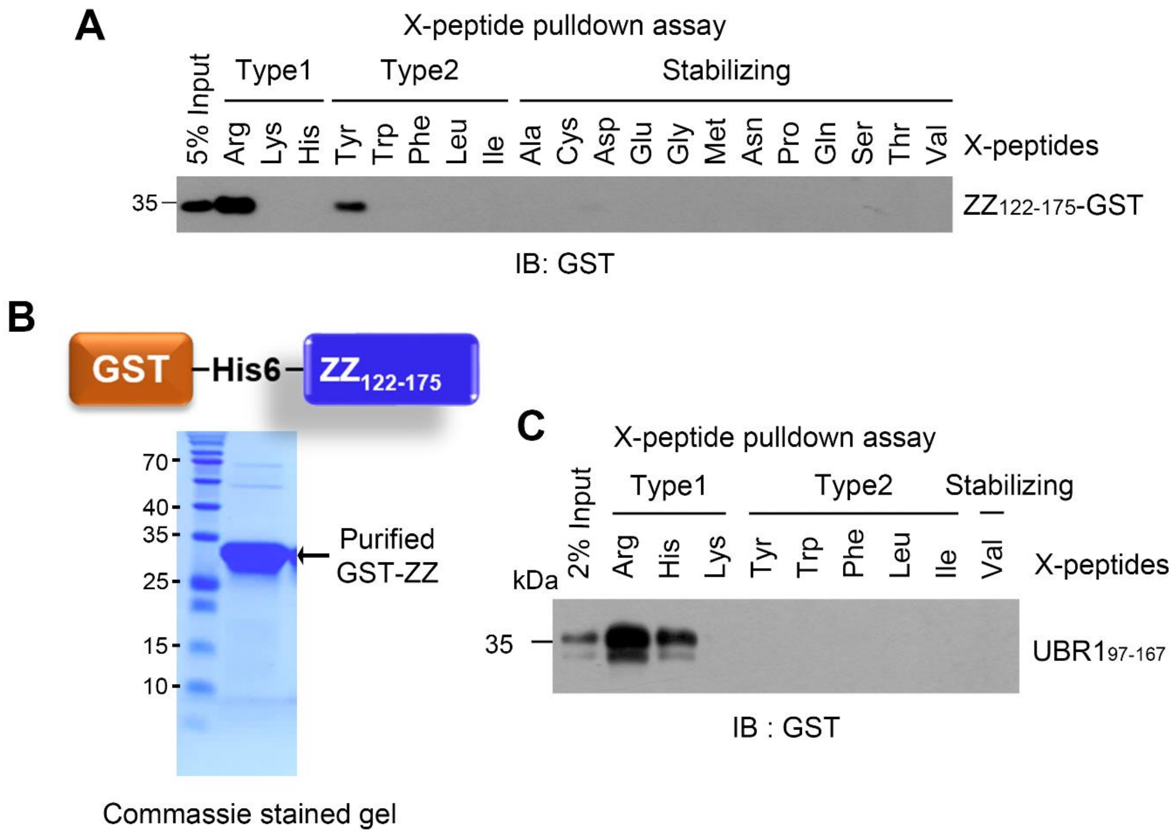

Supplementary Figure 1. p62 $ZZ_{122\text{-}175}$ exhibits strong and moderate binding to Nt-Arg and Nt-Tyr, respectively. a X-peptide pulldown assays to test $ZZ_{122\text{-}175}$ binding to 20 different N-terminal residues using biotinylated 11-mer X-peptides (X represents 20 essential amino acids) as ligands and p62 ZZ domain (122-175) transiently expressed in HEK293 cells. **b** Coomassie staining of purified GST-$ZZ_{122\text{-}175}$ expressed in *Escherichia coli*. **c** X-peptide pulldown assays to examine the binding ability of purified human UBR box (UBR1) with type 1 and type 2 N-degrons.

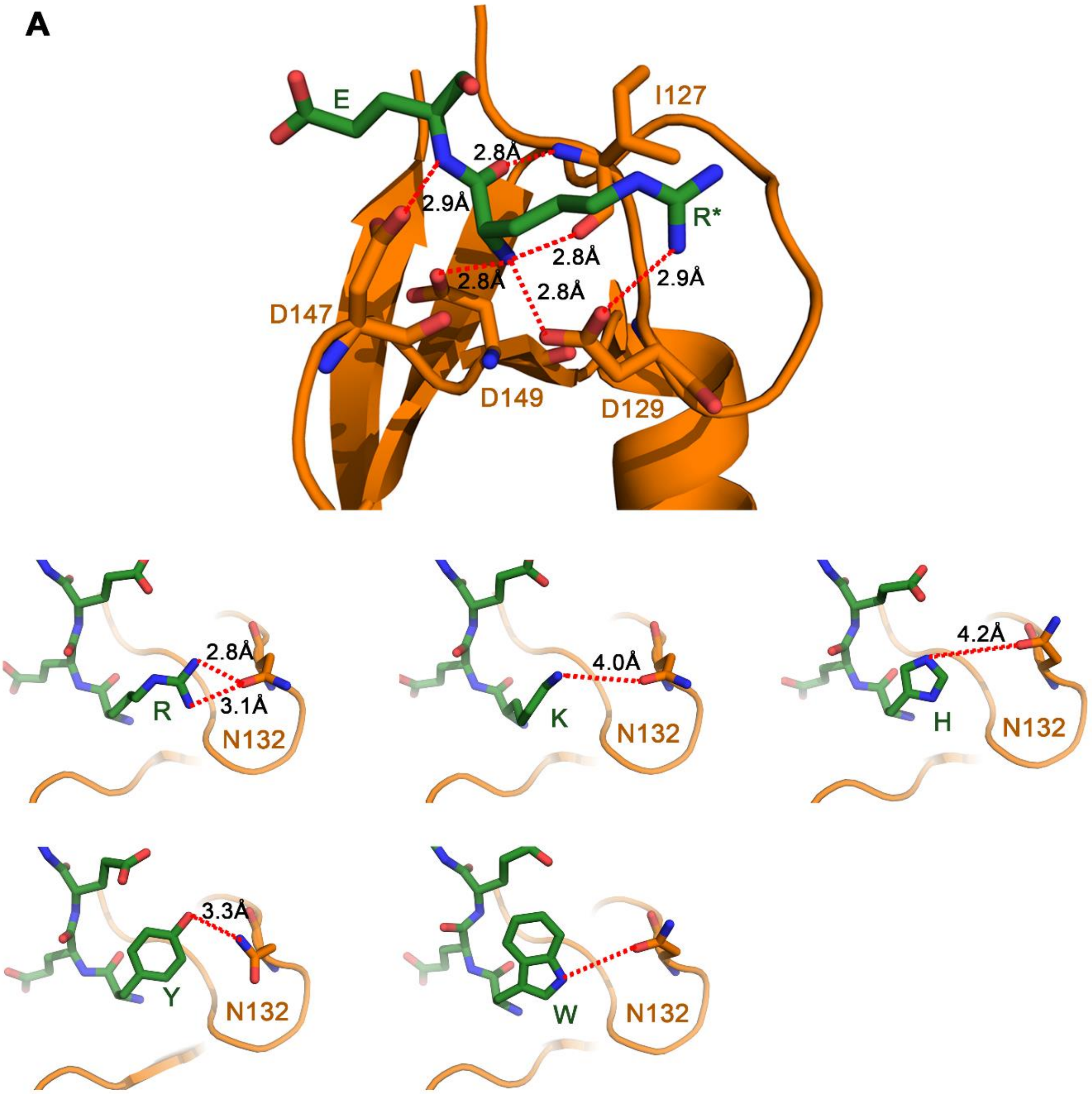

Supplementary Figure 2. Structural analysis of the p62 ZZ domain. **a** The interacting residues of p62 ZZ domain (orange color) with N-terminal residues of N-degron (green color) are presented as cartoon and stick model (PDB ID: 5YP8 (Arg), 5YPA (Lys), 5YPB (His), 5YPE (Tyr), 5YPF (Trp)). The interacting atoms are connected by red dot lines and the distances between interacting atoms are labeled.

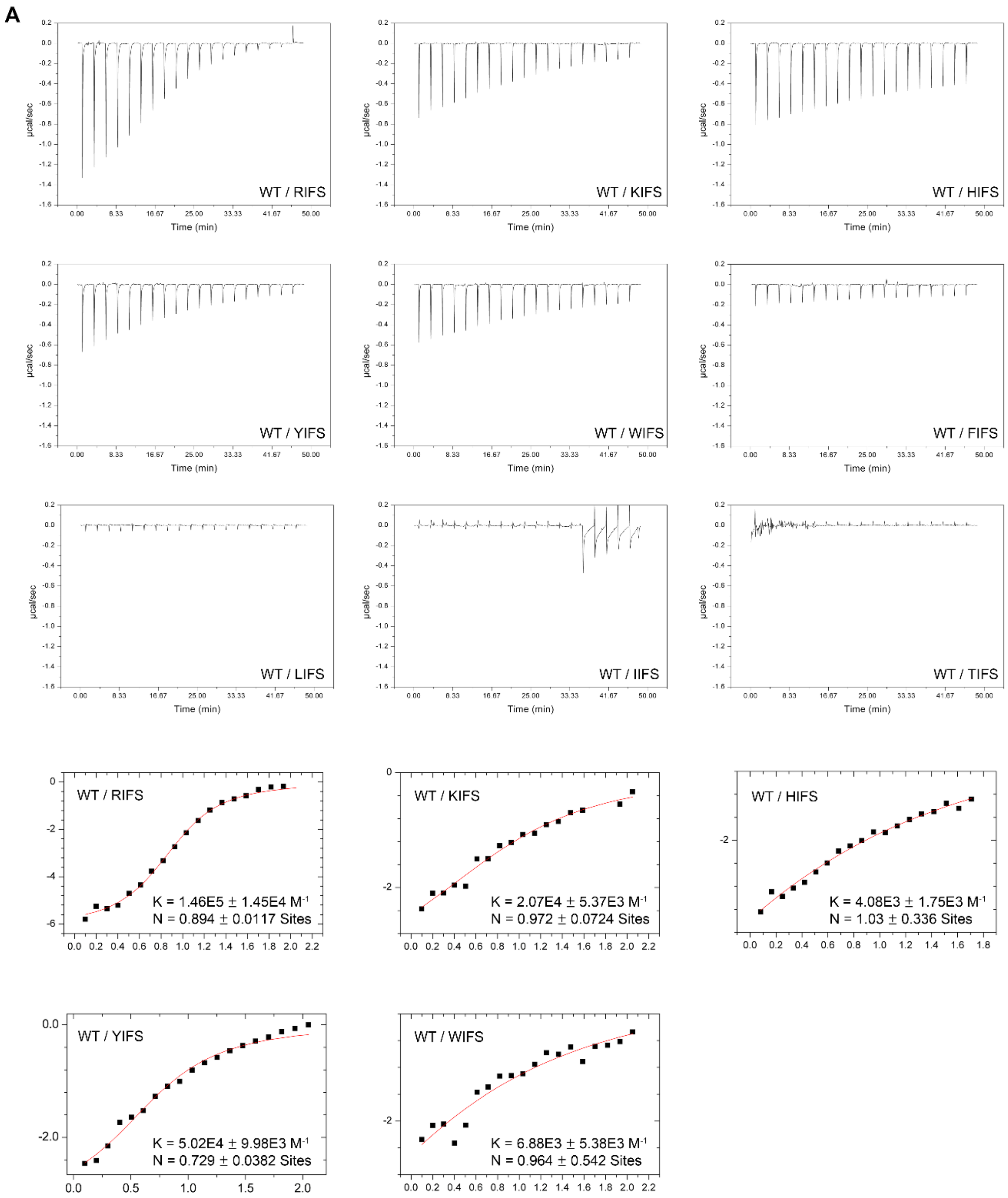
A
µcal/sec
Time (min)
WT / RIFS
WT / KIFS
WT / HIFS
WT / YIFS
WT / WIFS
WT / FIFS
WT / LIFS
WT / IIFS
WT / TIFS
WT / RIFS
K = 1.46E5 ± 1.45E4 M-1
N = 0.894 ± 0.0117 Sites
WT / KIFS
K = 2.07E4 ± 5.37E3 M-1
N = 0.972 ± 0.0724 Sites
WT / HIFS
K = 4.08E3 ± 1.75E3 M-1
N = 1.03 ± 0.336 Sites
WT / YIFS
K = 5.02E4 ± 9.98E3 M-1
N = 0.729 ± 0.0382 Sites
WT / WIFS
K = 6.88E3 ± 5.38E3 M-1
N = 0.964 ± 0.542 Sites

B

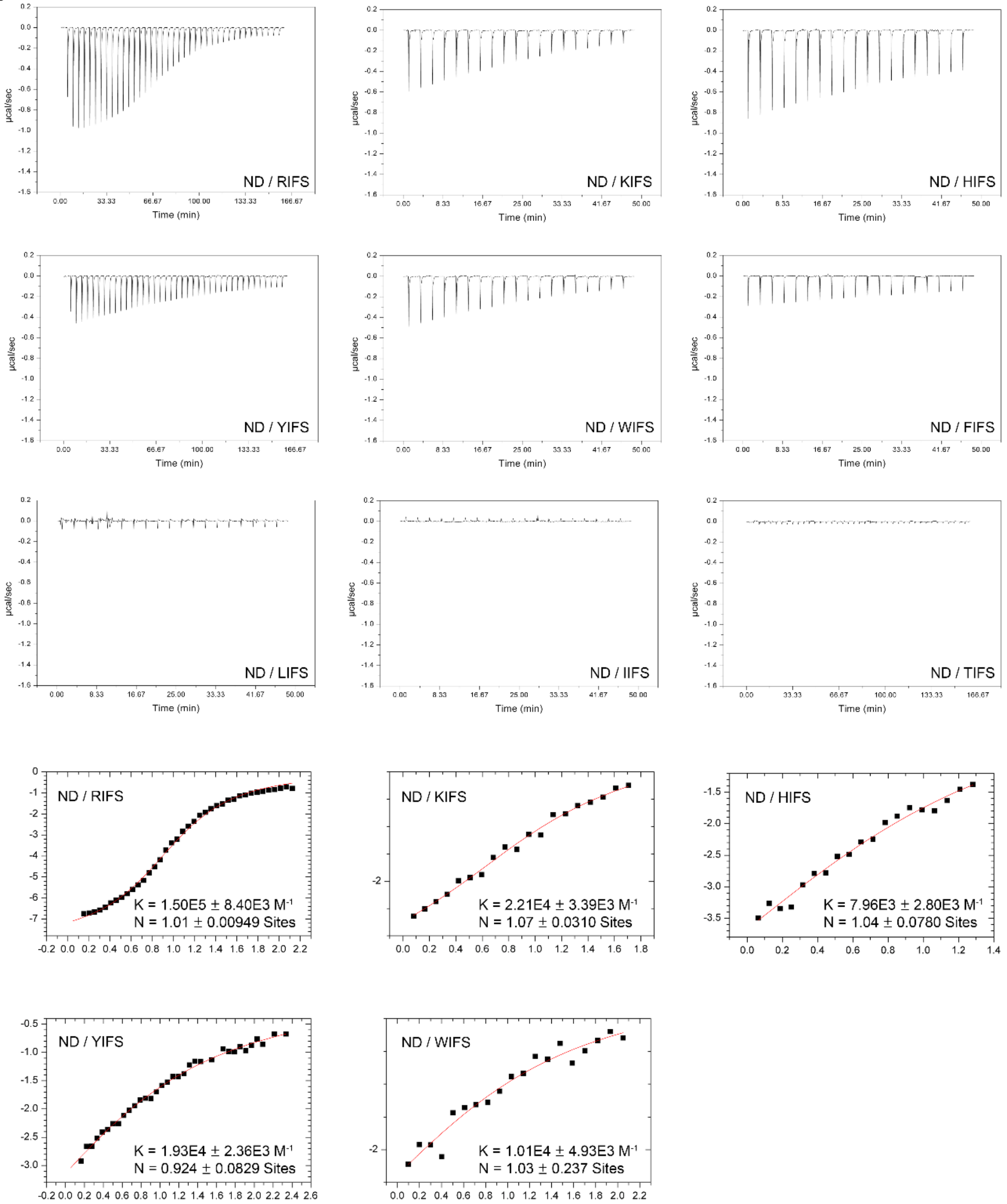

ND / RIFS
ND / KIFS
ND / HIFS
ND / YIFS
ND / WIFS
ND / FIFS
ND / LIFS
ND / IIFS
ND / TIFS
µcal/sec
Time (min)
ND / RIFS
K = 1.50E5 ± 8.40E3 M-1
N = 1.01 ± 0.00949 Sites
ND / KIFS
K = 2.21E4 ± 3.39E3 M-1
N = 1.07 ± 0.0310 Sites
ND / HIFS
K = 7.96E3 ± 2.80E3 M-1
N = 1.04 ± 0.0780 Sites
ND / YIFS
K = 1.93E4 ± 2.36E3 M-1
N = 0.924 ± 0.0829 Sites
ND / WIFS
K = 1.01E4 ± 4.93E3 M-1
N = 1.03 ± 0.237 Sites

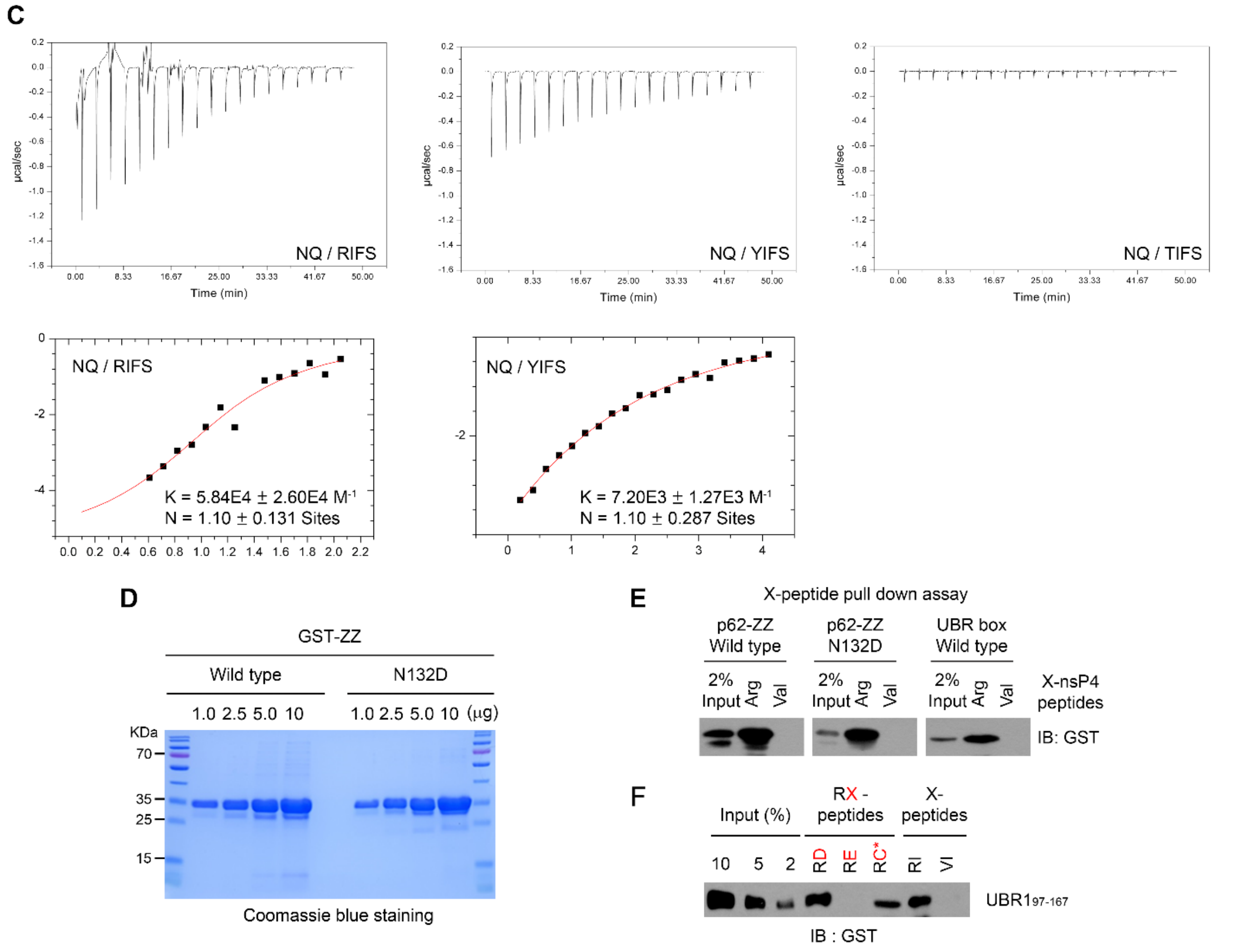

Supplementary Figure 3. Raw ITC data. a The raw ITC data of ZZ wild type with peptides are shown as power graphs and enthalpy ($\delta_b H$) graphs. **b-c** The raw ITC data of ZZ (N132D) and ZZ (N132Q) with peptides are also shown. Only enthalpy graphs were drawn for affinity data that showed binding, and dissociation constants (K_D) were calculated. FIFS, LIFS, IIFS, and TIFS peptides have no affinity for any ZZ domains. **d** Coomassie blue-stained bands of purified wild type and N132D ZZ tagged with Nt-GST. **e** *In vitro* peptide pulldown assays comparing the binding affinity of purified human p62-ZZ and p62-ZZ N132D with the UBR box of hUBR1. **f** *In vitro* peptide pulldown assays to examine the effect of the second residues of RX-peptides (X = Asp, Glu or oxidized Cys) on the binding of UBR box to Nt-Arg.

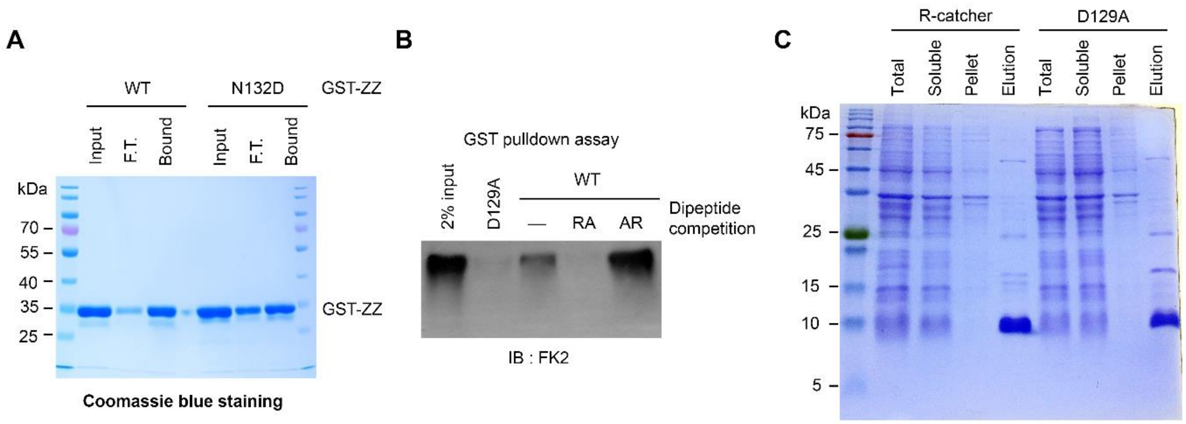

Supplementary Figure 4. Purified recombinant R-catcher. a Coomassie blue stained SDS-PAGE gel presenting purified wild type and N132D $ZZ_{122\text{-}175}$ tagged with Nt-GST (F.T. means flow through). **b** SDS-PAGE gel presenting samples of wild type and D129A mutant R-catcher from Ni-affinity column. **c** Immunoblotting analysis of poly-ubiquitinated proteins captured by R-catcher.

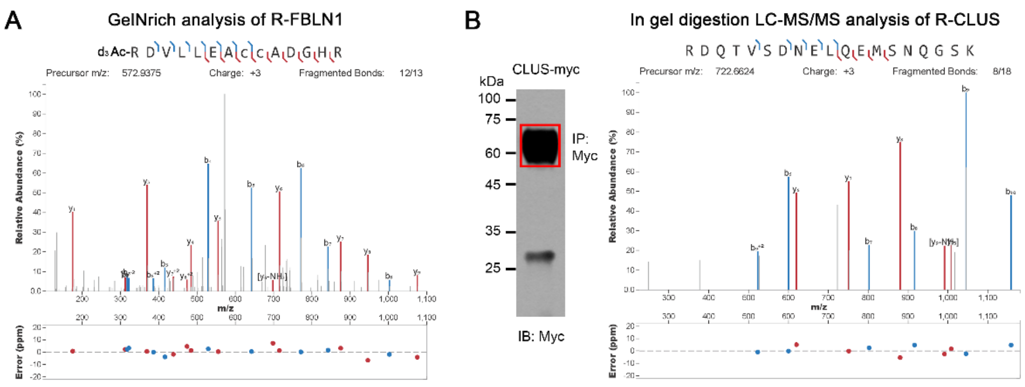

Supplementary Figure 5. Mass Spectrometry analysis of R-FBLN1 and CLUS. a Peptide spectrum of arginylated FBLN1 detected using GelNrich analysis**. b** Peptide spectrum of arginylated CLUS obtained by In-gel digestion and LC-MS/MS analysis.

A

Biological processes	Score (Grade)	Molecular functions	Score (Grade)	Cellular Componets	Score (Grade)
Response to Unfolded Protein	51.42 (High)	Unfolded Protein Binding	57.12 (High)	Extracellular Exosome	56.09 (High)
Protein Folding	28.97 (High)	Ubiquitin Protein Ligase Binding	44.27 (High)	Focal Adhension	29.17 (High)
Negative Regulation of Apoptotic Process	21.51 (High)	ATP binding	19.52 (High)	Cytosol	26.22 (High)
Regulation of mRNA stability	16.13 (High)	Structural Constituent of cytoskelecton	27.88 (High)	Nucleus	14.23 (High)
Membrane Organization	15.13 (High)	Chaperone Binding	27.8 (High)	Mitochondrion	13.55 (High)
Protein Stabilization	13.35 (High)	RNA Binding	26.2 (High)	Cytoskeleton	12.14 (High)
Neutrophil Degranulation	11.43 (High)	Protein Kinase Binding	11.52 (High)	Plasma Membrane	10.55 (High)
Cytokine-mediated Signaling Pathway	10.86 (High)	Cadherin Binding	10.47 (High)	Endoplasmic Reticulum	8.71 (High)

B

Diseases	Score (Grade)
Prostate Cancer	5.5 (High)
Amyotrophic Lateral Sclerosis	5.07 (High)
Ovarian Cancer	4.22 (High)
Systemic Lupus Erythematosus	2.83 (High)
Alzehiemer Disease	2.83 (Medium)
Muscular Dystrophy, Becker Type	2.82 (Medium)

Pathways	Score (Grade)
Cellular Response to Heat Stress	29.13 (High)
Metabolism of Proteins	28.62 (High)
Innate Immune System	20.5 (High)
Cellular Senescence	19.44 (High)
Regulation of PLK1 Activity	12.65 (Medium)
Vesicle-mediated Transport	9.43 (Medium)

Supplementary Figure 6. Score (Grade) based GeneAnalytics analysis shown in Figure 7. a Score (Grade) of biological processes, molecular functions and cellular components shown in Figure 7b according to GeneAnalytics. **b** Score (Grade) of diseases and pathways shown in Figure 7c according to GeneAnalytics.

Supplymentary table 1. ZZ domain containing human proteins.

Uniprot ID	Gene symbol	ZZ domain	Name	Synonyms	Chromosome
Q92793	CREBBP	1701-1744	CREB binding protein	RTS, CBP, KAT3A	16p13.3
P11531	DMD	3307-3354	Dystrophin	BMD, DXS142, DXS164	Xp212
Q13474	DRP2	604-651	Dystrophin related protein 2		Xq22
Q9Y4J8	DTNA	237-284	Dystrobrevin, alpha	D18S892E, DTN, DTN-1, DTN-2, DTN-3, DRP-3	18q12
O60941	DTNB	237-284	Dystrobrevin, beta		2p23.2
A2CJ06	DYTN	222-269	Dystrotelin		2q33.3
Q09472	EP300	1667-1707	E1A binding protein p300	P300, KAT3B	22q13.2
O95714	HERC2	2702-2749	HECT and RLD domain containing E3 ubiquitin protein ligase 2	Jdf2, p528, D15F37S1	15q13
Q14596	NBR1	211-257	Neighbor of BRCA1 gene 1	CA125, KIAA0049, 1A1-3B	17q21.31
Q13501	SQSTM1	122-167	Sequestosome 1	P62, p60, p62B, A170	5q35
Q86TJ2	TADA2B	3-49	Transcriptional adaptor 2B	MGC21874	4p16.1
P46939	UTRN	3064-3111	Utrophin	DRP, DRP1	6q24
Q9P0J7	KCMF1	3-50	Potassium channel modulatory factor 1	ZZZ1, PCMF, DEBT91, DKFZP434L1021	2p11.2
Q8NEG5	ZSWIM2	228-277	Zinc finger, SWIM-type containing 2	ZZZ2, MGC33890	2q32.2
Q8IYH5	ZZZ3	817-867	Zinc finger, ZZ-type containing 3	DKFZP5641052, ATAC1	1p31.1
O43149	ZZEF1-ZZ1	1777-1825	Zinc finger, ZZ-type with EF-hand domain 1	FLJ10821, ZZZ4, KIAA0399	17p13.3
O43149	ZZEF1-ZZ2	1826-1872	Zinc finger, ZZ-type with EF-hand domain 1	FLJ10821, ZZZ4, KIAA0399	17p13.3
Q96AX9	MIB2	143-190	Mindbomb E3 ubiquitin protein ligase 2	ZZZ5, skeletrophin, FLJ39787	1p36.33
Q86YT6	MIB1	79-126	Mindbomb E3 ubiquitin protein ligase 1	ZZZ6, KIAA1323, DIP-1, MIB, ZZANK2	18q11.2

Supplymentary table 2. Raw data WT R-catcher

Unique	XCorr	M+H+	CalcM+H+	PPM	TotalIntensity	Sequence
sp|P08670|VIME_HUMAN						
*	2.2703	967.52045	967.5207	-0.3	3668444.5	R.TYSLGSALR.P
*	4.7344	2498.2456	2497.2568	-5.8	1970403.1	R.LLQDSVDFSLADAINTEFKNTR.T
*	3.5068	1254.5674	1254.567	0.3	23043984	R.LGDLYEEEMR.E
*	3.2585	1088.5363	1088.5331	2.9	9371470	R.QDVDNASLAR.L
*	4.7295	1405.762	1405.7573	3.3	109378224	K.VESLQEEIAFLK.K
*	10.848	3923.0151	3923.0144	0.2	5014507	K.LHEEEIQELQAQIQEQHVQIDVDVSKPDLTAALR.D
*	9.4152	3923.019	3923.0144	1.2	43356020	K.LHEEEIQELQAQIQEQHVQIDVDVSKPDLTAALR.D
*	6.9899	3923.022	3923.0144	1.9	46257744	K.LHEEEIQELQAQIQEQHVQIDVDVSKPDLTAALR.D
	2.9989	1309.6075	1309.6058	1.3	12553989	K.NLQEAEEWYK.S
*	4.6286	1646.8541	1646.853	0.7	4858246	R.RQVQSLTCEVDALK.G
*	5.3544	1646.854	1646.853	0.6	20463312	R.RQVQSLTCEVDALK.G
*	4.6337	1490.7539	1490.7518	1.4	14698592	R.QVQSLTCEVDALK.G
*	7.1018	2186.9648	2186.9658	-0.4	15892469	R.EMEENFAVEAANYQDTIGR.L
*	6.0673	2186.968	2186.9658	1	16922464	R.EMEENFAVEAANYQDTIGR.L
*	5.0291	2202.957	2202.9607	-1.7	451914	R.EM(15.995)EENFAVEAANYQDTIGR.L
	3.6907	1295.6683	1295.6664	1.5	34898668	K.MALDIEIATYR.K
	3.4296	1311.6615	1311.6613	0.2	1375151.2	K.M(15.995)ALDIEIATYR.K
*	3.4306	1570.8982	1570.8951	1.9	161332848	R.ISLPLPNFSSLNLR.E
*	4.685	1668.8461	1668.8439	1.3	11038231	R.ETNLDSLPLVDTHSK.R
*	3.535	1837.7839	1836.7994	-10.3	1222800	R.DGQVINETSQHHDDLE.-
sp|P11021|BIP_HUMAN						
	7.8629	2444.2378	2444.2378	0	4414340.5	K.KEDVGTVVGIDLGTTYSCVGVFK.N
	3.545	1566.7815	1566.7798	1.1	52569024	R.ITPSYVAFTPEGER.L
	4.7624	1677.8115	1677.8079	2.2	16939776	K.NQLTSNPENTVFDAK.R
	3.7855	1430.6919	1430.691	0.6	31842464	R.TWNDPSVQQDIK.F
	4.138	1604.8662	1604.8643	1.2	1815798.4	K.TKPYIQVDIGGGQTK.T
	3.5686	1536.799	1536.7977	0.8	8514505	K.TFAPEEISAMVLTK.M
	4.5669	1887.9731	1887.9712	1	1839509.8	K.VTHAVVTVPAYFNDAQR.Q
	5.0052	1887.9719	1887.9712	0.4	26521952	K.VTHAVVTVPAYFNDAQR.Q
	3.2857	1217.6288	1217.6306	-1.5	2969261.2	K.DAGTIAGLNVMR.I
	5.6181	1659.8977	1659.8951	1.5	36247564	R.IINEPTAAAIAYGLDK.R
	3.318	1815.9973	1815.9963	0.5	508588	R.IINEPTAAAIAYGLDKR.E
	5.4133	1815.9976	1815.9963	0.7	14861459	R.IINEPTAAAIAYGLDKR.E
	5.6625	2165	2164.992	3.7	2304879	R.IEIESFYEGEDFSETLTR.A
	3.1396	1313.6204	1313.6194	0.7	32471246	K.FEELNMDLFR.S
	5.216	1836.9364	1836.9337	1.5	68546432	K.SQIFSTASDNQPTVTIK.V
	2.8485	1191.6373	1191.6368	0.4	65609124	K.VYEGERPLTK.D
	5.7976	1934.0104	1934.0131	-1.4	3243018	K.DNHLLGTFDLTGIPPAPR.G
	3.3812	1316.6385	1316.6368	1.3	61016268	R.NELESYAYSLK.N
	5.8846	1974.9137	1974.908	2.9	8482458	K.IEWLESHQDADIEDFK.A
	4.6517	2174.0408	2174.04	0.3	1647593.1	K.IEWLESHQDADIEDFKAK.K
	5.8189	1525.885	1525.8835	1	1895010.8	K.KELEEIVQPIISK.L
	3.6635	1397.7906	1397.7886	1.5	15852320	K.ELEEIVQPIISK.L
	4.9884	1818.8398	1818.8392	0.3	20519906	K.LYGSAGPPPTGEEDTAEK.D
	6.7646	2175.9937	2175.9927	0.4	11253754	K.LYGSAGPPPTGEEDTAEKDEL.-
	7.0356	2175.9958	2175.9927	1.5	4444765	K.LYGSAGPPPTGEEDTAEKDEL.-
sp|Q01105|SET_HUMAN						
*	5.7341	2100.146	2100.1448	0.6	1214905.8	K.KPRPPPALGPEETSASAGLPK.K
	7.7983	3449.67	3449.6665	1	20643284	K.EQQEAIEHIDEVQNEIDRLNEQASEEILK.V
	5.8572	3449.6687	3449.6665	0.6	27178684	K.EQQEAIEHIDEVQNEIDRLNEQASEEILK.V
	3.549	1208.6051	1208.6045	0.5	6785994	R.VEVTEFEDIK.S
	5.1942	1840.8074	1840.8064	0.5	5551758	R.IDFYFDENPYFENK.V
	4.4433	1446.6521	1446.6495	1.8	5073453.5	K.EFHLNESGDPSSK.S
	9.552	3377.536	3377.5344	0.4	8738415	K.RQHEEPESFFTWFTDHSDAGADELGEVIK.D
	8.8721	3377.54	3377.5344	1.7	10599846	K.RQHEEPESFFTWFTDHSDAGADELGEVIK.D
sp|P81605|DCD_HUMAN						
	3.0955	1128.5289	1128.528	0.9	8428489	K.ENAGEDPGLAR.Q
	4.0239	1217.6737	1217.6736	0.1	11981650	R.SSLLEKGLDGAK.K
*	4.1922	1867.94	1867.9396	0.2	4596406	K.DAVEDLESVGKGAVHDVK.D
sp|Q13162|PRDX4_HUMAN						
*	3.9824	2444.0847	2443.073	3.4	1916491.9	R.TREEECHFYAGGQVYPGEASR.V
*	4.3735	1779.8625	1779.8588	2.1	4928621	K.PAPYWEGTAVIDGEFK.E

*	4.635	1779.8611	1779.8588	1.3	2911679.8	K.PAPYWEGTAVIDGEFK.E
*	3.486	1624.7638	1624.7601	2.3	559180.5	K.DYGVYLEDSGHTLR.G
*	3.1051	1225.6904	1225.69	0.4	8585074	R.QITLNDLPVGR.S
*	2.9329	1212.6273	1212.6259	1.2	3216879.2	R.LVQAFQYTDK.H
	3.8783	1141.5294	1140.5255	0.5	3142391.5	K.HGEVCPAGWK.P
*	3.7953	1281.6691	1281.6685	0.5	818277.5	K.PGSETIIPDPAGK.L
sp|P10599-2|THIO_HUMAN						
sp|P10599|THIO_HUMAN						
	3.6093	1336.6406	1336.638	2	3073634.2	K.TAFQEALDAAGDK.L
	3.0779	1739.8257	1738.8291	-3.9	1345833.6	K.LVVVDFSATWCGPCK.M
sp|P62987|RL40_HUMAN						
sp|P62979|RS27A_HUMAN						
sp|P0CG48|UBC_HUMAN						
sp|P0CG47|UBB_HUMAN						
	5.2182	1788.9309	1787.9272	0.2	1499069.9	K.TITLEVEPSDTIENVK.A
	3.0088	1067.6212	1067.6207	0.5	1453634	K.ESTLHLVLR.L
sp|P68363-2|TBA1B_HUMAN						
sp|P68363|TBA1B_HUMAN						
	6.9474	2007.8956	2007.8931	1.3	10150427	K.TIGGGDDSFNTFFSETGAGK.H
	4.2628	1701.9083	1701.9058	1.5	10071918	R.AVFVDLEPTVIDEVR.T
	4.037	1487.8798	1487.8792	0.4	13306904	R.LISQIVSSITASLR.F
	2.3819	1015.578	1015.5783	-0.2	966680.8	K.DVNAAIATIK.T
	4.3364	1824.9868	1824.9854	0.8	5730081.5	K.VGINYQPPTVVPGGDLAK.V
	1.8098	2348.9053	2348.9048	0.2	228037.2	K.DYEEVGVDSVEGEGEEEGEEY.-
sp|P63208|SKP1_HUMAN						
	4.4513	1878.936	1878.9331	1.6	859037.7	K.LQSSDGEIFEVDVEIAK.Q
	5.5399	1761.8839	1761.8806	1.9	1515425.8	K.RTDDIPVWDQEFLK.V
*	3.7747	1466.6406	1466.6394	0.8	5421448	K.NDFTEEEEAQVR.K
sp|P67775-2|PP2AA_HUMAN						
sp|P67775|PP2AA_HUMAN						
	4.4732	1704.7908	1704.7897	0.6	2501631	K.ELDQWIEQLNECK.Q
	2.8594	1655.931	1655.9254	3.4	563701.9	R.GYYSVETVTLLVALK.V
	4.3735	1791.8386	1791.837	0.9	2745033	R.QITQVYGFYDECLR.K
	4.448	1703.8252	1703.821	2.4	5175175.5	R.NVVTIFSAPNYCYR.C
	3.3105	1340.6655	1340.6633	1.6	6735077	K.YSFLQFDPAPR.R
sp|O43813|LANC1_HUMAN						
*	2.0353	1299.6013	1299.6005	0.7	4491685.5	R.AFPNPYADYNK.S
*	2.858	1256.5898	1256.5906	-0.6	5186031.5	K.SLAEGYFDAAGR.L
*	2.5747	963.49304	963.4928	0.3	2827010	K.QSLNCLTK.R
*	6.5146	2203.1614	2203.158	1.6	3127484.8	R.SITFLCGDAGPLAVAAVLYHK.M
*	4.0629	1413.8164	1413.8141	1.6	15379349	R.IGYIYALLFVNK.N
*	3.3291	1208.5973	1208.5979	-0.5	783209.4	K.PSVDYVCQLK.F
*	3.8633	1593.713	1593.7114	1	829619.1	K.FPSGNYPPCIGDNR.D
*	4.7553	1814.7467	1813.7421	0.7	1152391.9	K.FAEWCLEYGEHGCR.T
sp|P0DMV8|HS71A_HUMAN						
sp|P0DMV9|HS71B_HUMAN						
	3.0264	1487.7035	1487.7013	1.5	7925956	R.TTPSYVAFTDTER.L
	4.6297	1658.8516	1658.8496	1.2	6225181	K.NQVALNPQNTVFDAK.R
	3.3177	1222.578	1222.5773	0.6	1624768	K.FGDPVVQSDMK.H
	2.7747	1238.5731	1238.5721	0.8	457136.8	K.FGDPVVQSDM(15.995)K.H
	5.6086	1680.8489	1680.8492	-0.2	8039937.5	K.HWPFQVINDGDKPK.V
	3.08	1614.8099	1614.8083	1	3989910	K.AFYPEEISSMVLTK.M
	3.4713	1197.6962	1197.695	1	38650008	K.DAGVIAGLNVLR.I
	5.5875	1687.9044	1687.9014	1.8	18597582	R.IINEPTAAAIAYGLDR.T
	5.4352	1675.7334	1675.7307	1.6	4632578.5	K.ATAGDTHLGGEDFDNR.L
	3.5721	1465.8135	1465.8121	0.9	2512582	K.AQIHDLVLVGGSTR.I
	5.4561	2786.3645	2786.363	0.5	2994288.5	K.QTQIFTTYSDNQPGVLIQVYEGER.A
	4.7191	2786.3782	2786.363	5.4	11456443	K.QTQIFTTYSDNQPGVLIQVYEGER.A
	2.7423	1204.534	1204.5328	1	10856843	K.GGSGSGPTIEEVD.-
sp|P08758|ANXA5_HUMAN						
*	3.2791	1340.6129	1340.6117	0.9	6950343.5	R.GTVTDFPGFDER.A
*	6.2007	2888.2405	2888.238	0.8	1291033.2	K.QVYEEEYGSSLEDDVVGDTSGYYQR.M
*	3.0963	1156.6884	1156.687	1.2	1962399.9	R.MLVVLLQANR.D
*	3.944	1803.872	1802.8629	3.1	526168.4	K.YMTISGFQIEETIDR.E
*	2.7321	1106.585	1106.584	0.9	17215850	R.SEIDLFNIR.K
*	2.6022	1118.5404	1118.5398	0.5	4278574.5	K.ALLLLCGEDD.-

sp|P07355-2|ANXA2_HUMAN						
sp|P07355|ANXA2_HUMAN						
	5.1436	1542.8514	1542.8485	1.9	7146572.5	K.GVDEVTIVNILTNR.S
	4.7557	1650.9794	1650.9789	0.3	12574203	K.SALSGHLETVILGLLK.T
	4.1239	1222.5962	1222.595	1	7598787	K.TPAQYDASELK.A
	5.5502	1777.8678	1777.8636	2.3	5152304	K.GLGTDEDSLIEIICSR.T
	3.1607	1244.624	1244.6229	0.9	4090252.5	R.TNQELQEINR.V
	2.867	1225.5703	1225.5695	0.7	2075255.8	K.DIISDTSGDFR.K
	3.0294	1461.6779	1460.6726	1.3	1573100.5	K.SYSPYDMLESIR.K
sp|P14618|KPYM_HUMAN						
	3.0548	1197.6497	1197.6473	1.9	713521.4	R.LDIDSPPITAR.N
	3.2061	1359.7056	1359.705	0.4	1227655.4	R.NTGIICTIGPASR.S
	5.6392	2465.297	2465.2922	2	7992331	R.TATESFASDPILYRPVAVALDTK.G
	4.1121	1468.6804	1468.6776	1.9	2857899	K.CDENILWLDYK.N
	4.4447	1779.8789	1779.876	1.6	3413463.5	K.GADFLVTEVENGGSLGSK.K
	4.1164	1636.8928	1636.8905	1.4	3960398.8	K.GVNLPGAAVDLPAVSEK.D
	4.0448	2175.117	2175.118	-0.4	2075228.4	R.LAPITSDPTEATAVGAVEASFK.C
	4.7288	1642.7733	1642.7708	1.6	2734106.5	K.DPVQEAWAEDVDLR.V
sp|Q9NUJ1|ABHDA_HUMAN						
	2.5971	1080.5386	1080.5394	-0.8	2867409.8	K.ALAIEEFCK.S
	4.5949	2035.8735	2035.8727	0.4	507893.8	R.FDYSGVGSSDGNSEESTLGK.W
	4.8042	1641.9823	1641.9785	2.3	28553952	K.VVALIGVATAADTLVTK.F
	5.949	1825.8661	1825.8643	1	19617788	K.YSEEGVYNVQYSFIK.E
	3.2182	1229.711	1229.71	0.9	8791881	R.VLSTDVDVILR.K
sp|Q07021|C1QBP_HUMAN						
*	3.6409	1283.6528	1283.6517	0.9	9694944	K.AFVDFLSDEIK.E
*	3.1729	1621.7538	1621.7526	0.8	260189.5	K.MSGGWELELNGTEAK.L
*	4.9978	3136.4058	3136.405	0.2	637201.2	K.ALVLDCHYPEDEVGQEDEAESDIFSIR.E
*	3.4378	1513.6841	1513.6805	2.3	1624228.2	R.EVSFQSTGESEWK.D
sp|Q71U36-2|TBA1A_HUMAN						
sp|Q71U36|TBA1A_HUMAN						
	6.9474	2007.8956	2007.8931	1.3	10150427	K.TIGGGDDSFNTFFSETGAGK.H
	4.2628	1701.9083	1701.9058	1.5	10071918	R.AVFVDLEPTVIDEVR.T
	2.3819	1015.578	1015.5783	-0.2	966680.8	K.DVNAAIATIK.T
	3.5933	1598.7677	1598.7672	0.3	2775321	R.TIQFVDWCPTGFK.V
	4.3364	1824.9868	1824.9854	0.8	5730081.5	K.VGINYQPPTVVPGGDLAK.V
	1.8098	2348.9053	2348.9048	0.2	228037.2	K.DYEEVGVDSVEGEGEEEGEEY.-
sp|P62714|PP2AB_HUMAN						
*	4.2642	1690.7769	1690.774	1.7	799013.7	K.ELDQWVEQLNECK.Q
	2.8594	1655.931	1655.9254	3.4	563701.9	R.GYYSVETVTLLVALK.V
	4.3735	1791.8386	1791.837	0.9	2745033	R.QITQVYGFYDECLR.K
	4.448	1703.8252	1703.821	2.4	5175175.5	R.NVVTIFSAPNYCYR.C
	3.3105	1340.6655	1340.6633	1.6	6735077	K.YSFLQFDPAPR.R
sp|Q9HCC0|MCCB_HUMAN						
	3.418	1706.8463	1706.8451	0.7	272985.6	R.VSGVECMIIANDATVK.G
*	4.214	1340.7957	1340.7936	1.5	3507747.2	K.QGTIFLAGPPLVK.A
	3.6507	1417.7124	1417.711	0.9	13375598	K.AFYGDTLVTGFAR.I
	4.486	1802.9672	1802.968	-0.5	4261044	R.ISVMGGEQAANVLATITK.D
	3.0049	1746.9288	1746.9272	0.9	257601.7	K.QFSSADEAALKEPIIK.K
	4.1287	1548.6615	1548.6602	0.9	1812375.2	K.FEEEGNPYYSSAR.V
	4.042	1472.7064	1472.7015	3.3	5097946	R.VWDDGIIDPADTR.L
	3.8754	1743.0093	1743.0051	2.4	951032.3	R.LVLGLSFSAALNAPIEK.T
sp|P04792|HSPB1_HUMAN						
*	4.292	1902.868	1902.8671	0.5	3827812	R.GPSWDPFRDWYPHSR.L
*	3.0633	1075.5751	1075.5742	0.8	10838590	R.QLSSGVSEIR.H
*	5.6306	1905.9951	1905.9916	1.9	8977199	K.LATQSNEITIPVTFESR.A
sp|Q9BQE3|TBA1C_HUMAN						
	6.9474	2007.8956	2007.8931	1.3	10150427	K.TIGGGDDSFNTFFSETGAGK.H
	4.2628	1701.9083	1701.9058	1.5	10071918	R.AVFVDLEPTVIDEVR.T
	4.037	1487.8798	1487.8792	0.4	13306904	R.LISQIVSSITASLR.F
	2.3819	1015.578	1015.5783	-0.2	966680.8	K.DVNAAIATIK.T
	3.5933	1598.7677	1598.7672	0.3	2775321	R.TIQFVDWCPTGFK.V
	4.3364	1824.9868	1824.9854	0.8	5730081.5	K.VGINYQPPTVVPGGDLAK.V
sp|Q86UD7-3|TBC26_HUMAN						
*	1.3483	2033.7712	2033.7833	-5.9	3822312.2	A.CACCVGGCFRDWESGGR.-
*	1.1591	1873.7417	1873.7527	-5.9	5504680.5	C.ACCVGGCFRDWESGGR.-

sp|Q9UFN0|NPS3A_HUMAN						
*	3.2622	1337.6187	1337.6161	1.9	17907960	R.QYDGIFYEFR.S
*	5.1184	1652.8005	1652.7988	1	12004766	K.QESEITYLVPWCK.L
*	4.0423	1215.6161	1215.6157	0.3	902624.4	K.PGGPALWGDAFK.R
*	3.0053	1346.7758	1346.7751	0.5	2933777.5	N.MLLIPTSFSPLK.-
*	1.0199	1102.6512	1102.6506	0.6	214160.4	L.LIPTSFSPLK.-
sp|Q13405|RM49_HUMAN						
*	3.8992	1647.7762	1646.7697	2	402121.3	R.FVESVDEYQFVER.L
*	3.0432	1514.8181	1514.8173	0.6	498956.1	K.TPVTQVNEVTGTLR.I
sp|P37108|SRP14_HUMAN						
*	2.8762	1068.5945	1068.5935	0.9	2527511.2	R.TSGSVYITLK.K
*	3.4813	1263.5869	1263.5852	1.4	5837661	K.GTVEGFEPADNK.C
sp|Q06830|PRDX1_HUMAN						
*	2.5844	1107.6049	1107.6044	0.4	2467243.2	R.TIAQDYGVLK.A
	3.8783	1141.5294	1140.5255	0.5	3142391.5	K.HGEVCPAGWK.P
*	5.7559	2406.1897	2406.187	1.1	2380686.2	K.HGEVCPAGWKPGSDTIKPDVQK.S
sp|P68366-2|TBA4A_HUMAN						
sp|P68366|TBA4A_HUMAN						
	4.3431	2067.897	2067.8965	0.2	1216819.5	K.TIGGGDDSFTTFFCETGAGK.H
	3.8945	1715.9258	1715.9214	2.6	3132760.8	R.AVFVDLEPTVIDEIR.N
	4.037	1487.8798	1487.8792	0.4	13306904	R.LISQIVSSITASLR.F
	4.3364	1824.9868	1824.9854	0.8	5730081.5	K.VGINYQPPTVVPGGDLAK.V
sp|O75629|CREG1_HUMAN						
*	5.9392	2273.1968	2273.1924	1.9	6774667.5	R.FVTHVSDWGALATISTLEAVR.G
*	3.7509	1554.7695	1554.7686	0.6	2145991.8	K.IVTPEEYYNVTVQ.-
sp|P61289-2|PSME3_HUMAN						
sp|P61289|PSME3_HUMAN						
sp|P61289-3|PSME3_HUMAN						
	3.5986	1680.8496	1680.8479	1	578718.2	R.ITSEAEDLVANFFPK.K
	3.4011	1286.6952	1286.6951	0.1	1141543.2	K.SNQQLVDIIEK.V
	4.2679	1668.8092	1668.8075	1	1231437.8	R.TVESEAASYLDQISR.Y
sp|P34931|HS71L_HUMAN						
	3.0264	1487.7035	1487.7013	1.5	7925956	R.TTPSYVAFTDTER.L
	3.08	1614.8099	1614.8083	1	3989910	K.AFYPEEISSMVLTK.L
	3.4713	1197.6962	1197.695	1	38650008	K.DAGVIAGLNVLR.I
	5.6181	1659.8977	1659.8951	1.5	36247564	R.IINEPTAAAIAYGLDK.G
	5.4352	1675.7334	1675.7307	1.6	4632578.5	K.ATAGDTHLGGEDFDNR.L
	5.4561	2786.3645	2786.363	0.5	2994288.5	K.QTQIFTTYSDNQPGVLIQVYEGER.A
	4.7191	2786.3782	2786.363	5.4	11456443	K.QTQIFTTYSDNQPGVLIQVYEGER.A
sp|P07437|TBB5_HUMAN						
*	3.0546	1301.639	1301.6372	1.4	3088339.2	R.ISVYYNEATGGK.Y
	7.1243	1958.9858	1958.9818	2.1	1558851.2	K.GHYTEGAELVDSVLDVVR.K
	5.0603	1958.9855	1958.9818	1.9	5611316	K.GHYTEGAELVDSVLDVVR.K
	2.3736	1039.5938	1039.5935	0.2	3352862.5	R.YLTVAAVFR.G
	3.3217	1446.6902	1446.6893	0.6	1673434	K.EVDEQMLNVQNK.N
	4.463	1696.8362	1696.8329	1.9	10418327	K.NSSYFVEWIPNNVK.T
sp|P25398|RS12_HUMAN						
*	2.7365	1061.5453	1061.5448	0.5	1453394.9	K.LGEWVGLCK.I
*	3.147	1106.5698	1106.5697	0.1	1096193	K.VVGCSCVVVK.D
sp|Q9Y2S7|PDIP2_HUMAN						
*	3.8662	1316.748	1316.746	1.6	7234612.5	K.VLETVGVFEVPK.Q
*	3.9382	1830.9164	1830.9174	-0.5	3189444.5	K.YETGQLFLHSIFGYR.G
*	3.841	1479.7623	1479.759	2.2	1222811.6	K.THTYYQVLIDAR.D
*	3.544	1322.7313	1322.7314	-0.1	5670518.5	R.IFSLSGTLETVR.G
sp|Q8NBS9-2|TXND5_HUMAN						
sp|Q8NBS9|TXND5_HUMAN						
	3.0067	1761.7668	1761.7643	1.5	700757	K.VDCTAHSDVCSAQGVR.G
	5.6681	2193.157	2193.155	0.9	5210523	K.ALAPTWEQLALGLEHSETVK.I
	2.053	1152.6205	1152.62	0.4	7441956.5	R.GYPTLLWFR.D
sp|P52815|RM12_HUMAN						
*	2.7942	1360.7595	1360.7583	0.9	2698990	K.NYIQGINLVQAK.K
*	2.7185	1228.6794	1228.6783	0.9	1304482.2	K.AALEAVGGTVVLE.-
sp|P02545-3|LMNA_HUMAN						
sp|P02545|LMNA_HUMAN						
	2.8581	1148.5809	1148.5793	1.4	5913304.5	R.ITESEEVVSR.E
	2.6996	1165.5468	1165.5483	-1.4	1364785.8	K.AAYEAELGDAR.K

	3.5779	1893.9285	1893.9263	1.2	398822.9	R.MQQQLDEYQELLDIK.L
	2.5816	1291.6287	1291.6277	0.8	400838.8	R.QNGDDPLLTYR.F
	3.1441	1406.6405	1406.6403	0.2	1420848.1	R.TVLCGTCGQPADK.A
	3.6969	1566.7512	1566.7507	0.3	830564.4	R.SVGGSGGGSFGDNLVTR.S
sp|P17066|HSP76_HUMAN						
	3.0264	1487.7035	1487.7013	1.5	7925956	R.TTPSYVAFTDTER.L
	5.5875	1687.9044	1687.9014	1.8	18597582	R.IINEPTAAAIAYGLDR.R
	5.4352	1675.7334	1675.7307	1.6	4632578.5	K.ATAGDTHLGGEDFDNR.L
*	3.1804	1721.8085	1720.8071	-1.2	2079392	R.LYGGPGVPGGSSCGTQAR.Q
*	3.2683	1456.6835	1456.6802	2.3	483248.2	R.QGDPSTGPIIEEVD.-
sp|P22830-2|HEMH_HUMAN						
sp|P22830|HEMH_HUMAN						
	3.2808	2699.279	2699.277	0.7	312765	R.AIAFTQYPQYSCSTTGSSLNAIYR.Y
	5.1096	2699.2773	2699.277	0.1	1370780.4	R.AIAFTQYPQYSCSTTGSSLNAIYR.Y
	2.9141	1276.6569	1276.6532	2.9	1756932.8	R.AESLNGNPLFSK.A
	5.0954	1816.8883	1816.8866	0.9	2419227.5	K.QLTLSCPLCVNPVCR.E
sp|P31151|S10A7_HUMAN						
*	5.2363	1384.7124	1384.7107	1.2	5902140.5	K.KGTNYLADVFEK.K
*	3.4919	1256.6173	1256.6157	1.3	2053655	K.GTNYLADVFEK.K
sp|O43707-3|ACTN4_HUMAN						
sp|O43707|ACTN4_HUMAN						
	3.2764	1507.7034	1507.7023	0.7	351439	K.AGTQIENIDEDFR.D
	2.6631	1920.0084	1920.0072	0.6	278952.8	K.LSGSNPYTTVTPQIINSK.W
	3.7187	1386.7759	1386.7739	1.4	2084218.8	R.VGWEQLLTTIAR.T
	3.3004	1741.8148	1741.8126	1.3	796653.5	R.ETTDTDTADQVIASFK.V
sp|P10909-2|CLUS_HUMAN						
sp|P10909|CLUS_HUMAN						
sp|P10909-5|CLUS_HUMAN						
sp|P10909-4|CLUS_HUMAN						
sp|P10909-3|CLUS_HUMAN						
	3.6547	1393.6973	1393.6958	1.1	6242910.5	R.ASSIIDELFQDR.F
	5.2205	1762.8287	1762.8276	0.6	3913937.5	R.EILSVDCSTNNPSQAK.L
	3.2398	1288.6387	1288.638	0.6	5228183	R.ELDESLQVAER.L
	5.3471	1874.9951	1873.9906	0.6	6910942.5	K.LFDSDPITVTVPVEVSR.K
sp|P10809|CH60_HUMAN						
*	5.598	2365.3367	2365.3337	1.2	1200184.9	R.KPLVIIAEDVDGEALSTLVLNR.L
*	5.6763	2038.026	2038.0226	1.7	1021602.4	R.IQEIIEQLDVTTSEYEK.E
*	3.6507	1233.5957	1233.5957	0	8535203	K.VGGTSDVEVNEK.K
*	3.1481	1215.6587	1215.658	0.6	1779811.5	K.NAGVEGSLIVEK.I
sp|P25705-2|ATPA_HUMAN						
sp|P25705|ATPA_HUMAN						
	3.6897	1575.7915	1575.786	3.5	878653.4	R.ILGADTSVDLEETGR.V
	2.9515	1624.8916	1624.8904	0.8	1450812.6	R.TGAIVDVPVGEELLGR.V
	3.4914	1171.6334	1171.6317	1.5	1040430.7	R.VVDALGNAIDGK.G
	3.1337	1316.7433	1316.742	1	1101836	K.TSIAIDTIINQK.R
sp|P11142|HSP7C_HUMAN						
	3.0264	1487.7035	1487.7013	1.5	7925956	R.TTPSYVAFTDTER.L
	3.4259	1649.7971	1649.7952	1.2	888720.3	K.NQVAMNPTNTVFDAK.R
	5.6181	1659.8977	1659.8951	1.5	36247564	R.IINEPTAAAIAYGLDK.K
	3.4841	1691.7277	1691.7256	1.2	1128178.5	K.STAGDTHLGGEDFDNR.M
*	3.5315	1304.6315	1304.6304	0.8	2774037.8	K.CNEIINWLDK.N
sp|Q13501-2|SQSTM_HUMAN						
sp|Q13501|SQSTM_HUMAN						
	6.8601	2239.9106	2239.9126	-0.9	4718934	R.YKCSVCPDYDLCSVCEGK.G
	5.8316	1948.7559	1948.7544	0.8	14144584	K.CSVCPDYDLCSVCEGK.G
	5.2714	1948.7549	1948.7544	0.3	50408532	K.CSVCPDYDLCSVCEGK.G
	4.1834	2412.0205	2412.0198	0.3	2863546.5	K.CSVCPDYDLCSVCEGKGLHR.G
	3.4968	1671.8243	1671.8224	1.2	985355.7	K.NYDIGAALDTIQYSK.H
sp|P30153|2AAA_HUMAN						
	3.5622	1242.7429	1242.7416	1.1	3402213	K.LSTIALALGVER.T
*	3.2736	1109.5381	1109.5375	0.6	2171886.2	R.LAGGDWFTSR.T
*	2.9603	1400.5571	1400.5569	0.2	2522198	K.EFCENLSADCR.E
	6.4327	1942.0519	1942.0426	4.8	8596041	R.LNIISNLDCVNEVIGIR.Q
*	3.1105	1160.58	1160.5793	0.5	4660798.5	K.LTQDQDVDVK.Y
sp|P31689|DNJA1_HUMAN						
	4.005	1451.7401	1451.7377	1.7	1320290.8	K.QISQAYEVLSDAK.K

	3.996	1392.8224	1392.8209	1.1	2090412.8	R.TIVITSHPGQIVK.H
*	3.1241	1463.7227	1463.7166	4.2	1109878.1	K.VNFPENGFLSPDK.L
sp|P63244|RACK1_HUMAN						
*	2.6714	1264.6539	1264.6532	0.6	881446.3	R.LWDLTTGTTTR.R
*	2.9559	1309.6423	1309.6383	3.1	488505	K.DVLSVAFSSDNR.Q
*	1.2813	960.52576	960.5261	-0.4	9693954	V.WQVTIGTR.-
sp|P08865|RSSA_HUMAN						
*	5.0783	1740.952	1740.949	1.8	4293577.5	R.AIVAIENPADVSVISSR.N
*	2.656	1306.6472	1306.646	0.9	1436803.1	R.YVDIAIPCNNK.G
sp|P50454|SERPH_HUMAN						
*	4.3403	1659.8032	1659.8013	1.2	2727583.5	R.LYGPSSVSFADDFVR.S
*	3.656	1224.6583	1224.6582	0.1	3464495.5	K.GVVEVTHDLQK.H
*	3.2932	1293.6812	1293.6797	1.1	2551926.2	R.DTQSGSLLFIGR.L
sp|P28066|PSA5_HUMAN						
*	3.4631	1423.7859	1423.7831	2	880494.4	R.LFQVEYAIEAIK.L
	3.4358	1216.6315	1216.6307	0.6	1724858.1	K.EELEEVIKDI.-
sp|O43852-10|CALU_HUMAN						
	2.7932	1294.6176	1294.6161	1.1	1260028.5	K.TFDQLTPEESK.E
*	1.1049	1080.4957	1080.4857	9.3	1771956.2	G.WQAYQGGDR.-
sp|P27797|CALR_HUMAN						
*	2.6247	1219.7058	1219.7045	1.1	910011.6	K.GQTLVVQFTVK.H
*	4.383	2710.0464	2708.0337	2.2	492202.1	K.DEDEEDEEDKEEDEEEDVPGQAK.D
*	4.1464	1474.622	1474.6179	2.7	3822528	K.EEDEEEDVPGQAK.D
sp|Q9BPW8|NIPS1_HUMAN						
*	2.2168	1010.4326	1010.4326	-0.1	2467247.5	K.DNEGSWFR.S
*	4.5728	1710.8495	1710.8485	0.6	9459266	R.GWDENVYYTVPLVR.H
sp|P31943|HNRH1_HUMAN						
	3.9905	1841.8949	1841.8916	1.8	1330294	R.STGEAFVQFASQEIAEK.A
	4.3251	1996.9829	1996.9763	3.3	2063088.8	R.ATENDIYNFFSPLNPVR.V
sp|Q96AY3|FKB10_HUMAN						
*	3.2206	1509.791	1509.7908	0.2	579698.7	R.ASPAGGPLEDVVIER.Y
*	3.7595	1796.9246	1796.921	2	626040.5	K.DAVQLETLELPPGCVR.R
	2.2086	1324.5961	1324.595	0.8	286494.9	K.TIGDMFQNQDR.N
sp|P60709|ACTB_HUMAN						
sp|P63261|ACTG_HUMAN						
	2.1902	1132.5264	1132.527	-0.5	2408382.8	R.GYSFTTTAER.E
	4.2344	1790.8928	1790.8918	0.5	7365815	K.SYELPDGQVITIGNER.F
sp|P08238|HS90B_HUMAN						
	3.4708	1242.705	1242.7052	-0.2	1629297.1	K.ADLINNLGTIAK.S
	3.0737	1311.5719	1311.5698	1.6	1808335.2	K.EDQTEYLEER.R
*	3.3476	1848.7981	1847.797	-1.2	279937.6	R.NPDDITQEEYGEFYK.S
	3.5504	1416.6388	1416.6376	0.9	2094238.6	K.EGLELPEDEEEK.K
sp|P63151-2|2ABA_HUMAN						
sp|P63151|2ABA_HUMAN						
	3.0333	1676.7303	1676.7295	0.5	473287.4	K.LCSLYENDCIFDK.F
	5.5076	2067.076	2067.0757	0.1	1051355.6	K.ENIIAVATTNNLYIFQDK.V
sp|P58107|EPIPL_HUMAN						
*	5.1178	1884.9937	1884.9912	1.3	1159671.4	R.LSVEEAVAAGVVGGEIQEK.L
*	3.7546	2164.087	2164.0823	2.1	1285196.8	K.GFFDPNTHENLTYVQLLR.R
*	4.3319	2564.317	2564.3103	2.6	4179572.5	R.LTAIIEEAEEAPGARPQLQDAWR.G
sp|Q08380|LG3BP_HUMAN						
*	3.8514	1592.7925	1592.7915	0.6	3830668.5	R.ELSEALGQIFDSQR.G
*	2.9089	1355.7778	1355.7781	-0.2	4153239	R.SDLAVPSELALLK.A
*	2.7998	1206.5548	1206.5538	0.8	1578016.8	K.AVDTWSWGER.A
sp|Q5XKE5|K2C79_HUMAN						
	3.439	1329.7279	1329.726	1.5	10802431	R.NLDLDSIIAEVK.A
	3.544	1196.558	1196.5582	-0.2	3280706.8	R.AEAEAWYQTK.Y
	3.2856	1263.6959	1263.6943	1.3	5014557	K.LALDVEIATYR.K
sp|P52272-2|HNRPM_HUMAN						
sp|P52272|HNRPM_HUMAN						
	3.0301	1264.6943	1264.6936	0.6	2846752	R.AFITNIPFDVK.W
	4.0186	1752.875	1752.8724	1.5	620621	K.VGEVTYVELLMDAEGK.S
	3.5023	1426.7601	1426.7577	1.7	452432	R.LGSTVFVANLDYK.V
sp|P38646|GRP75_HUMAN						
*	3.0001	1694.8486	1694.8496	-0.6	689383.5	K.NAVITVPAYFNDSQR.Q
*	3.5456	1361.742	1361.7423	-0.3	3192614.8	R.AQFEGIVTDLIR.R

*	3.1663	1290.681	1290.68	0.8	1278368.8	K.VQQTVQDLFGR.A
sp|Q14247|SRC8_HUMAN						
	2.3973	1085.5276	1085.5262	1.2	480483.9	Q.SAVGFEYQGK.T
*	3.292	1200.548	1200.5531	-4.3	849053.7	R.QDSAAVGFDYK.E
sp|P68104-2|EF1A1_HUMAN						
sp|Q5VTE0|EF1A3_HUMAN						
sp|P68104|EF1A1_HUMAN						
	3.1243	1404.7278	1404.727	0.5	979611.1	K.YYVTIIDAPGHR.D
	2.6529	1025.6105	1025.6102	0.2	2069180.4	K.IGGIGTVPVGR.V
sp|P23142-2|FBLN1_HUMAN						
sp|P23142|FBLN1_HUMAN						
sp|P23142-4|FBLN1_HUMAN						
sp|P23142-3|FBLN1_HUMAN						
	3.1338	1922.7373	1922.7378	-0.3	212777.7	R.DSSCGTGYELTEDNSCK.D
	2.678	1179.5522	1178.5476	1.1	1253946.9	K.TGYYFDGISR.M
sp|P48681|NEST_HUMAN						
*	3.5712	1372.6724	1372.6703	1.5	3345426	R.DNLAEELEGVAGR.C
*	3.2026	1554.8253	1554.8235	1.2	581731.8	R.AQDAPLSLLQTQGGR.K
*	3.5065	1401.7025	1401.7008	1.2	604454.6	R.GEGEGQIWGLVEK.E
*	4.1062	1848.888	1848.8861	1	771331.6	K.DLEEAGGLGTEFSELPGK.S
sp|P11498|PYC_HUMAN						
*	5.0694	1828.8894	1828.8865	1.6	1680235.5	R.GANAVGYTNYPDNVVFK.F
*	4.7185	1547.7354	1547.7336	1.1	1225413	R.AEAEAQAEELSFPR.S
*	2.7273	958.53235	958.5316	0.8	3308350.5	K.ALAVSDLNR.A
sp|P14625|ENPL_HUMAN						
*	3.7521	1786.9038	1785.8977	1.5	329839.2	R.EEEAIQLDGLNASQIR.E
*	2.4358	1150.5394	1150.5375	1.7	570935.5	K.EAESSPFVER.L
sp|Q16531|DDB1_HUMAN						
*	3.1588	1218.6337	1218.6324	1	1555059.4	K.QSGESIDIITR.A
*	2.5166	1098.6932	1098.6921	1	1036381.9	K.YLAIAPPIIK.Q
*	2.9284	1480.7224	1480.7213	0.7	764515.4	R.QGQGQLVTCSGAFK.E
sp|A5A3E0|POTEF_HUMAN						
*	1.1566	1963.7509	1962.7648	-8.8	112010.6	K.WCCRCFPCCRESGK.S
	4.2344	1790.8928	1790.8918	0.5	7365815	K.SYELPDGQVITIGNER.F
sp|P13639|EF2_HUMAN						
*	3.1691	1594.7651	1594.7628	1.5	193630.2	R.ETVSEESNVLCLSK.S
*	2.3676	1138.5162	1138.5164	-0.1	823719.5	K.YEWDVAEAR.K
sp|P21333-2|FLNA_HUMAN						
sp|P21333|FLNA_HUMAN						
	3.6608	1226.7732	1226.7719	1.1	4935519	R.LIALLEVLSQK.K
	3.0545	1909.92	1909.9177	1.2	632475	R.EGPYSISVLYGDEEVPR.S
	3.792	1426.7549	1426.7537	0.9	564411.3	R.EAGAGGLAIAVEGPSK.A
	3.0445	2258.0276	2258.028	-0.2	210044.6	K.DGSCGVAYVVQEPGDYEVSVK.F
sp|Q13813-2|SPTN1_HUMAN						
sp|Q13813|SPTN1_HUMAN						
sp|Q13813-3|SPTN1_HUMAN						
	2.6917	1205.6064	1205.6049	1.3	727272.5	R.DVDETISWIK.E
	3.21	1213.6947	1213.694	0.6	3139301.5	R.AALLELWELR.R
	3.1182	1737.8293	1737.829	0.2	467357.3	K.TATDEAYKDPSNLQGK.V
	2.2362	1108.5637	1108.5632	0.4	771014.8	K.LLVGSEDYGR.D
	2.8006	1204.6794	1204.6783	0.9	1622769	R.DLSSVQTLLTK.Q
sp|O75533|SF3B1_HUMAN						
*	2.8809	1801.9092	1801.9065	1.5	301748.8	K.LLVDVDESTLSPEEQK.E
*	3.4321	1330.7228	1330.7212	1.2	946119.1	R.QLVDTTVELANK.V
sp|Q14315-2|FLNC_HUMAN						
sp|Q14315|FLNC_HUMAN						
	3.6608	1226.7732	1226.7719	1.1	4935519	R.LIALLEVLSQK.R
	3.2694	1601.8177	1601.817	0.5	343384.4	R.SPFEVQVSPEAGVQK.V
	4.0217	1584.8243	1584.8228	1	324114.3	R.GAGTGGLGLAIEGPSEAK.M

Supplymentary Table 3. Raw data WT R-catcher with AR competition

Unique	XCorr	M+H+	CalcM+H+	PPM	otalIntensit	Sequence
sp|P04792|HSPB1_HUMAN						
*	4.8177	1902.869	1902.867	1.2	22416124	R.GPSWDPFRDWYPHSR.L
*	3.1552	1163.622	1163.621	0.6	40831108	R.LFDQAFGLPR.L
*	7.4872	4093.066	4093.061	1.3	10368805	R.LPEEWSQWLGGSSWPGYVRPLPPAAIESPAVAAPAYSR.A
*	6.3365	4093.07	4093.061	2.3	13373401	R.LPEEWSQWLGGSSWPGYVRPLPPAAIESPAVAAPAYSR.A
*	3.1578	1075.574	1075.574	-0.6	9598944	R.QLSSGVSEIR.H
*	5.0711	1783.925	1783.923	1.2	890722	R.VSLDVNHFAPDELTVK.T
*	2.5513	917.494	917.4938	0.1	13607840	K.DGVVEITGK.H
*	7.4163	3098.564	3098.56	1.2	4803567	K.YTLPPGVDPTQVSSSLSPEGTLTVEAPMPK.L
*	4.9587	1905.994	1905.992	1.3	29435258	K.LATQSNEITIPVTFESR.A
sp|P08670|VIME_HUMAN						
*	2.2681	967.5208	967.5207	0.1	12961242	R.TYSLGSALR.P
*	2.776	1428.711	1428.712	-0.7	9528861	R.SLYASSPGGVYATR.S
	2.7909	1115.571	1115.569	1.6	1.81E+08	K.VELQELNDR.F
*	3.3692	1169.715	1169.714	1.1	1.08E+08	K.ILLAELEQLK.G
*	3.4473	1254.568	1254.567	1	20181530	R.LGDLYEEEMR.E
*	3.2859	1088.534	1088.533	1	2409587	R.QDVDNASLAR.L
*	3.8244	1405.764	1405.757	4.6	1.5E+08	K.VESLQEEIAFLK.K
*	3.055	1533.858	1533.852	3.6	699254.1	K.VESLQEEIAFLKK.L
*	8.892	3923.019	3923.014	1.1	27808554	K.LHEEEIQELQAQIQEQHVQIDVDVSKPDLTAALR.D
*	7.1928	3923.024	3923.014	2.6	46817112	K.LHEEEIQELQAQIQEQHVQIDVDVSKPDLTAALR.D
	3.0899	1309.61	1309.606	3.1	46262704	K.NLQEAEEWYK.S
*	5.0584	1646.855	1646.853	1.1	25836120	R.RQVQSLTCEVDALK.G
*	4.9988	1646.854	1646.853	0.3	4999376	R.RQVQSLTCEVDALK.G
*	4.6007	1490.754	1490.752	1.2	19494612	R.QVQSLTCEVDALK.G
*	6.9244	2186.963	2186.966	-1.1	19028780	R.EMEENFAVEAANYQDTIGR.L
*	5.4058	2186.968	2186.966	1	18879676	R.EMEENFAVEAANYQDTIGR.L
*	2.788	2202.96	2202.961	-0.4	482503.7	R.EM(15.995)EENFAVEAANYQDTIGR.L
	2.975	1121.584	1121.584	0.5	49102564	R.EYQDLLNVK.M
	3.4887	1295.668	1295.666	1.3	5193001	K.MALDIEIATYR.K
	3.4734	1311.662	1311.661	0.2	1920654	K.M(15.995)ALDIEIATYR.K
*	3.262	1570.899	1570.895	2.7	4717015	R.ISLPLPNFSSLNLR.E
*	4.6449	1668.846	1668.844	1.2	7029614	R.ETNLDSLPLVDTHSK.R
*	4.1896	1836.798	1836.799	-0.9	6489770	R.DGQVINETSQHHDDLE.-
contaminant_UBIQUITIN08						
sp|P62987|RL40_HUMAN						
sp|P62979|RS27A_HUMAN						
sp|P0CG48|UBC_HUMAN						
sp|P0CG47|UBB_HUMAN						
	5.3734	1787.928	1787.927	0.3	2246540	K.TITLEVEPSDTIENVK.A
	2.6338	1081.553	1081.552	0.8	24139096	R.TLSDYNIQK.E
	3.2915	1067.621	1067.621	0.2	4619800	K.ESTLHLVLR.L
sp|P11021|BIP_HUMAN						
	8.0966	2444.241	2444.238	1.1	7143517	K.KEDVGTVVGIDLGTTYSCVGVFK.N
	3.7684	1566.782	1566.78	1.3	2054491	R.ITPSYVAFTPEGER.L
	5.3044	1677.81	1677.808	1	5644431	K.NQLTSNPENTVFDAK.R
	3.8009	1430.692	1430.691	0.7	23452194	R.TWNDPSVQQDIK.F
	3.508	1604.868	1604.864	2.2	2950608	K.TKPYIQVDIGGGQTK.T
	5.2997	1604.864	1604.864	-0.1	10181673	K.TKPYIQVDIGGGQTK.T
	3.2705	1537.8	1536.798	-0.6	1709730	K.TFAPEEISAMVLTK.M
	3.8933	1887.97	1887.971	-0.5	1615321	K.VTHAVVTVPAYFNDAQR.Q
	4.7031	1887.972	1887.971	0.4	17838604	K.VTHAVVTVPAYFNDAQR.Q
	3.2219	1217.631	1217.631	0.5	3140589	K.DAGTIAGLNVMR.I
	4.8208	1659.899	1659.895	2.1	12702546	R.IINEPTAAAIAYGLDK.R
	3.3868	1815.998	1815.996	0.9	530597.2	R.IINEPTAAAIAYGLDKR.E
	6.0824	2164.993	2164.992	0.6	32864544	R.IEIESFYEGEDFSETLTR.A
	4.3252	2164.994	2164.992	1.1	6933032	R.IEIESFYEGEDFSETLTR.A
	3.1444	1313.621	1313.619	1.1	29153376	K.FEELNMDLFR.S
	4.6076	1460.762	1460.759	1.7	28540414	K.SDIDEIVLVGGSTR.I
	5.1529	1836.936	1836.934	1.3	21231478	K.SQIFSTASDNQPTVTIK.V
	4.8314	1934.013	1934.013	0	1003058	K.DNHLLGTFDLTGIPPAPR.G
	4.2713	1934.018	1934.013	2.5	2755924	K.DNHLLGTFDLTGIPPAPR.G
	4.9858	1974.91	1974.908	1.2	829387.4	K.IEWLESHQDADIEDFK.A

	5.481	1974.912	1974.908	1.8	5980435	K.IEWLESHQDADIEDFK.A
	5.8397	1525.885	1525.884	1.1	1893860	K.KELEEIVQPIISK.L
	4.041	1397.791	1397.789	1.7	10635903	K.ELEEIVQPIISK.L
	4.9274	1818.839	1818.839	-0.1	13210553	K.LYGSAGPPPTGEEDTAEK.D
	6.7123	2175.993	2175.993	0.2	9497963	K.LYGSAGPPPTGEEDTAEKDEL.-
	6.2065	2175.994	2175.993	0.8	1736894	K.LYGSAGPPPTGEEDTAEKDEL.-
sp|P0DMV8|HS71A_HUMAN						
sp|P0DMV9|HS71B_HUMAN						
	3.052	1487.703	1487.701	0.9	3841529	R.TTPSYVAFTDTER.L
	4.8534	1658.852	1658.85	1.3	23741294	K.NQVALNPQNTVFDAK.R
	3.1826	1222.578	1222.577	0.7	2867917	K.FGDPVVQSDMK.H
	2.1769	1238.572	1238.572	0.2	330929.8	K.FGDPVVQSDM(15.995)K.H
	6.2329	1680.849	1680.849	0	15383118	K.HWPFQVINDGDKPK.V
	3.129	1614.81	1614.808	0.8	5676068	K.AFYPEEISSMVLTK.M
	3.492	1197.697	1197.695	1.3	47052752	K.DAGVIAGLNVLR.I
	4.6927	1687.906	1687.901	2.6	29593658	R.IINEPTAAAIAYGLDR.T
	4.9763	1675.733	1675.731	1.6	6645407	K.ATAGDTHLGGEDFDNR.L
	3.4423	1675.731	1675.731	0.3	21347504	K.ATAGDTHLGGEDFDNR.L
	3.7038	1261.658	1261.658	0.1	8366388	R.LVNHFVEEFK.R
	3.3602	1542.74	1542.737	2.1	3356229	R.ARFEELCSDLFR.S
	3.1491	1315.6	1315.599	0.9	27896786	R.FEELCSDLFR.S
	4.7086	1465.813	1465.812	0.7	3455497	K.AQIHDLVLVGGSTR.I
	6.4454	2786.363	2786.363	0	4629315	K.QTQIFTTYSDNQPGVLIQVYEGER.A
	5.6017	2786.368	2786.363	1.9	31202058	K.QTQIFTTYSDNQPGVLIQVYEGER.A
	3.0309	1183.648	1183.647	0.7	2801654	R.FELSGIPPAPR.G
	3.2484	1287.605	1287.604	0.7	7262432	K.NALESYAFNMK.S
	5.1022	1876.912	1876.911	0.5	1821380	K.CQEVISWLDANTLAEK.D
	9.1617	3055.493	3055.494	-0.4	10330692	K.ELEQVCNPIISGLYQGAGGPGPGGFGAQGPK.G
	3.0622	1204.534	1204.533	0.8	4933844	K.GGSGSGPTIEEVD.-
sp|Q99417|MYCBP_HUMAN						
*	2.444	933.5264	933.5252	1.4	1038356	K.SGVLDTLTK.V
*	4.7786	2275.225	2275.222	1.4	3463523	K.VLVALYEEPEKPNSALDFLK.H
*	2.4882	1094.537	1094.536	0.8	1696110	K.YEAIVEENK.K
sp|P68363-2|TBA1B_HUMAN						
sp|P68363|TBA1B_HUMAN						
	7.2957	2007.895	2007.893	0.8	16446812	K.TIGGGDDSFNTFFSETGAGK.H
	4.414	1701.907	1701.906	0.9	17363270	R.AVFVDLEPTVIDEVR.T
	4.1211	1487.882	1487.879	2	11154716	R.LISQIVSSITASLR.F
	3.9394	1584.75	1584.752	-1.1	4055450	R.SIQFVDWCPTGFK.V
	4.5754	1824.988	1824.985	1.3	6671799	K.VGINYQPPTVVPGGDLAK.V
	6.3456	2330.022	2330.018	1.5	3624178	R.AFVHWYVGEGMEEGEFSEAR.E
	3.2531	2348.908	2348.905	1.1	553701.4	K.DYEEVGVDSVEGEGEEEGEEY.-
sp|P08865|RSSA_HUMAN						
*	5.7146	2617.278	2617.276	0.7	964780.1	K.FLAAGTHLGGTNLDFQMEQYIYK.R
*	3.0931	1135.638	1135.636	2.4	9677421	K.SDGIYIINLK.R
*	4.5747	1740.951	1740.949	1.2	10294731	R.AIVAIENPADVSVISSR.N
*	3.4709	1203.65	1203.648	1.9	5073424	K.FAAATGATPIAGR.F
*	5.7703	2996.478	2996.478	-0.2	3653492	R.ADHQPLTEASYVNLPTIALCNTDSPLR.Y
*	2.7261	1306.647	1306.646	0.8	3239947	R.YVDIAIPCNNK.G
sp|Q07021|C1QBP_HUMAN						
*	3.4552	1283.654	1283.652	1.6	5167459	K.AFVDFLSDEIK.E
*	3.2781	1621.754	1621.753	1.1	450181.5	K.MSGGWELELNGTEAK.L
*	3.6887	2287.173	2287.17	1.3	2299698	K.VEEQEPELTSTPNFVVEVIK.N
*	5.3233	3136.4	3136.405	-1.6	903074.4	K.ALVLDCHYPEDEVGQEDEAESDIFSIR.E
*	3.4183	1513.684	1513.681	1.9	1741331	R.EVSFQSTGESEWK.D
sp|P07355-2|ANXA2_HUMAN						
sp|P07355|ANXA2_HUMAN						
	2.4322	1086.482	1086.485	-2.9	6613381	K.AYTNFDAER.D
	4.658	1542.851	1542.849	1.4	6174600	K.GVDEVTIVNILTNR.S
	2.9395	1111.554	1111.553	0.4	1964834	R.QDIAFAYQR.R
	4.6586	1650.98	1650.979	0.9	6875808	K.SALSGHLETVILGLLK.T
	4.086	1222.596	1222.595	0.5	5672111	K.TPAQYDASELK.A
	6.5361	1777.875	1777.864	6.2	6625049	K.GLGTDEDSLIEIICSR.T
	2.887	1244.625	1244.623	1.8	3935263	R.TNQELQEINR.V
	2.6909	1225.57	1225.57	0.7	1737828	K.DIISDTSGDFR.K
	2.6586	1460.673	1460.673	-0.1	2571595	K.SYSPYDMLESIR.K

sp|P08758|ANXA5_HUMAN						
*	3.298	1340.614	1340.612	1.9	1286874	R.GTVTDFPGFDER.A
*	6.3403	2888.24	2888.238	0.7	1797317	K.QVYEEEYGSSLEDDVVGDTSGYYQR.M
*	6.6441	2888.242	2888.238	1.5	8830067	K.QVYEEEYGSSLEDDVVGDTSGYYQR.M
*	3.1633	1156.689	1156.687	1.5	1964586	R.MLVVLLQANR.D
*	3.6193	1802.863	1802.863	0.1	535540.5	K.YMTISGFQIEETIDR.E
*	2.8072	1106.585	1106.584	0.9	22779854	R.SEIDLFNIR.K
*	2.8682	1274.644	1274.645	-0.6	4497381	K.NFATSLYSMIK.G
*	3.2413	1118.54	1118.54	0.4	8028058	K.ALLLLCGEDD.-
sp|P80723|BASP1_HUMAN						
	2.7703	1427.666	1427.665	0.4	2997487	K.ESEPQAAAEPAEAK.E
*	2.6182	1966.941	1966.935	2.7	405105	K.AAEAAAAPAESAAPAAGEEPSK.E
	5.7009	2892.424	2892.422	0.5	966916.5	K.AQGPAASAEEPKPVEAPAANSDQTVTVKE.-
sp|P31943|HNRH1_HUMAN						
*	2.8638	1504.688	1504.685	1.7	1599141	R.GLPWSCSADEVQR.F
*	4.858	1764.82	1764.817	1.7	559777.3	R.PSGEAFVELESEDEVK.L
	3.4605	1684.771	1684.767	2	1078318	K.HTGPNSPDTANDGFVR.L
	6.9471	1841.896	1841.892	2.2	13753355	R.STGEAFVQFASQEIAEK.A
*	3.043	2717.05	2717.045	1.9	197199.3	R.GAYGGGYGGYDDYNGYNDGYGFGSDR.F
*	2.4549	2717.049	2717.045	1.5	592958.1	R.GAYGGGYGGYDDYNGYNDGYGFGSDR.F
	2.2615	1601.651	1601.647	2.5	361833.2	R.DLNYCFSGMSDHR.Y
	5.0126	1996.981	1996.976	2.3	10218354	R.ATENDIYNFFSPLNPVR.V
	5.1728	1996.982	1996.976	3.1	15758678	R.ATENDIYNFFSPLNPVR.V
	2.7636	1092.578	1092.58	-1.2	8628529	R.VHIEIGPDGR.V
sp|P30050|RL12_HUMAN						
	4.3826	1418.697	1418.695	1.5	1061165	R.CTGGEVGATSALAPK.I
*	3.9354	1666.976	1666.974	1	1975047	R.QAQIEVVPSASALIIK.A
	4.3429	1675.808	1675.807	0.6	1082003	K.EILGTAQSVGCNVDGR.H
sp|P10809|CH60_HUMAN						
	6.1306	2561.25	2560.249	-0.6	937615.3	K.LVQDVANNTNEEAGDGTTTATVLAR.S
	5.4155	2560.25	2560.249	0.7	3165500	K.LVQDVANNTNEEAGDGTTTATVLAR.S
*	6.0735	2384.231	2384.23	0.1	3884596	K.QSKPVTTPEEIAQVATISANGDK.E
*	2.8351	1190.611	1190.609	1.7	2932471	K.EIGNIISDAMK.K
*	2.9017	1389.707	1389.705	1.8	2436254	R.GYISPYFINTSK.G
*	6.6401	2365.338	2365.334	1.7	7306097	R.KPLVIIAEDVDGEALSTLVLNR.L
*	3.1719	912.588	912.5877	0.3	3826886	K.VGLQVVAVK.A
*	5.6638	2038.027	2038.023	2.1	3230517	R.IQEIIEQLDVTTSEYEK.E
*	3.7153	1233.596	1233.596	0.4	13847132	K.VGGTSDVEVNEK.K
*	5.1088	1684.907	1684.905	1.3	8495774	R.AAVEEGIVLGGGCALLR.C
*	3.166	1215.659	1215.658	0.6	2818911	K.NAGVEGSLIVEK.I
sp|P81605|DCD_HUMAN						
	3.9874	1217.674	1217.674	0.2	15259022	R.SSLLEKGLDGAK.K
	3.114	1161.563	1161.563	-0.2	13831352	K.DAVEDLESVGK.G
*	5.8028	1867.937	1867.94	-1.6	2053129	K.DAVEDLESVGKGAVHDVK.D
sp|P11142|HSP7C_HUMAN						
	3.052	1487.703	1487.701	0.9	3841529	R.TTPSYVAFTDTER.L
	3.6992	1649.798	1649.795	1.5	1213656	K.NQVAMNPTNTVFDAK.R
	3.3209	1254.567	1254.567	0.3	786198.3	R.FDDAVVQSDMK.H
	3.0826	1616.79	1616.788	1.3	5382365	K.SFYPEEVSSMVLTK.M
	3.8757	1981.999	1981.998	0.4	2786220	K.TVTNAVVTVPAYFNDSQR.Q
	4.8208	1659.899	1659.895	2.1	12702546	R.IINEPTAAAIAYGLDK.K
	3.5579	1691.727	1691.726	1.1	1308304	K.STAGDTHLGGEDFDNR.M
	3.3858	1481.81	1481.807	1.9	1323266	K.SQIHDIVLVGGSTR.I
	6.6557	2260.153	2260.146	3.2	5297564	K.SINPDEAVAYGAAVQAAILSGDK.S
	4.4033	2774.325	2774.327	-0.4	784132.9	K.QTQTFTTYSDNQPGVLIQVYEGER.A
	5.0178	2774.331	2774.327	1.6	8708825	K.QTQTFTTYSDNQPGVLIQVYEGER.A
*	3.6353	1304.632	1304.63	0.9	3422985	K.CNEIINWLDK.N
sp|P06748-2|NPM_HUMAN						
sp|P06748|NPM_HUMAN						
sp|P06748-3|NPM_HUMAN						
	4.69	1568.732	1568.73	1.6	1442478	K.VDNDENEHQLSLR.T
	5.2064	2227.218	2227.216	1.2	2319760	K.MSVQPTVSLGGFEITPPVVLR.L
	5.7265	4246.552	4246.55	0.5	920040.8	K.LAADEDDDDDDEEDDDEDDDDDDFDDEEAEEKAPVKK.G
sp|P63208|SKP1_HUMAN						
	4.3449	1878.936	1878.933	1.5	864678.3	K.LQSSDGEIFEVDVEIAK.Q
	4.9429	1761.883	1761.881	1.3	1170465	K.RTDDIPVWDQEFLK.V

	4.1026	1761.883	1761.881	1.1	7004473	K.RTDDIPVWDQEFLK.V
*	3.7024	1466.64	1466.639	0.7	4565837	K.NDFTEEEEAQVR.K
sp|Q9Y5L4|TIM13_HUMAN						
*	2.7939	1242.678	1242.676	1.4	1412481	K.LDPGLIMEQVK.V
*	4.6575	1552.882	1552.881	1.2	3955253	K.VQIAVANAQELLQR.M
sp|P38646|GRP75_HUMAN						
*	2.859	2079.027	2078.026	-0.9	214939.2	K.GAVVGIDLGTTNSCVAVMEGK.Q
*	3.3719	1450.719	1450.717	1.3	9975604	R.TTPSVVAFTADGER.L
*	4.3918	1568.77	1568.77	-0.2	3526653	R.QAVTNPNNTFYATK.R
*	4.1535	1333.639	1333.638	0.8	2854638	K.ETAENYLGHTAK.N
*	3.7206	1694.85	1694.85	0.4	1830258	K.NAVITVPAYFNDSQR.Q
*	3.4611	1242.681	1242.68	0.5	5381707	K.DAGQISGLNVLR.V
*	5.9124	2055.963	2055.962	0.8	3468733	K.STNGDTFLGGEDFDQALLR.H
*	3.573	1361.743	1361.742	0.2	6472590	R.AQFEGIVTDLIR.R
*	3.2549	1290.682	1290.68	1.1	4492221	K.VQQTVQDLFGR.A
*	4.3822	1808.904	1808.903	0.7	522736.3	K.SQVFSTAADGQTQVEIK.V
*	4.4035	1592.955	1592.952	1.8	1661369	K.LLGQFTLIGIPPAPR.G
*	3.4667	1473.793	1473.791	1.2	3322756	R.EQQIVIQSSGGLSK.D
sp|Q71U36-2|TBA1A_HUMAN						
sp|Q71U36|TBA1A_HUMAN						
	7.2957	2007.895	2007.893	0.8	16446812	K.TIGGGDDSFNTFFSETGAGK.H
	4.414	1701.907	1701.906	0.9	17363270	R.AVFVDLEPTVIDEVR.T
	3.5498	1598.767	1598.767	-0.2	3130021	R.TIQFVDWCPTGFK.V
	4.5754	1824.988	1824.985	1.3	6671799	K.VGINYQPPTVVPGGDLAK.V
	6.3456	2330.022	2330.018	1.5	3624178	R.AFVHWYVGEGMEEGEFSEAR.E
	3.2531	2348.908	2348.905	1.1	553701.4	K.DYEEVGVDSVEGEGEEEGEEY.-
sp|P07437|TBB5_HUMAN						
*	3.1254	1301.639	1301.637	1.3	7245093	R.ISVYYNEATGGK.Y
	3.997	1615.837	1615.836	0.4	4254685	R.AILVDLEPGTMDSVR.S
	8.7496	2798.348	2798.344	1.7	10732236	R.SGPFGQIFRPDNFVFGQSGAGNNWAK.G
*	3.9488	1659.898	1659.895	1.8	1407625	R.ALTVPELTQQVFDAK.N
	2.4645	1039.594	1039.594	0.2	4975216	R.YLTVAAVFR.G
	3.5734	1446.69	1446.689	0.4	4134744	K.EVDEQMLNVQNK.N
	4.9284	1696.835	1696.833	1.2	21538244	K.NSSYFVEWIPNNVK.T
	2.3318	1028.519	1028.519	-0.1	1062831	K.TAVCDIPPR.G
sp|P05387|RLA2_HUMAN						
*	4.2992	1772.904	1772.903	1	654882.5	K.ILDSVGIEADDDRLNK.V
*	3.3606	1256.686	1256.684	1.2	13768082	K.NIEDVIAQGIGK.L
sp|Q9NUJ1|ABHDA_HUMAN						
	2.5205	1080.54	1080.539	0.5	2786214	K.ALAIEEFCK.S
	5.2124	2035.87	2035.873	-1.4	1633714	R.FDYSGVGSSDGNSEESTLGK.W
	4.7963	1641.982	1641.979	1.9	13497870	K.VVALIGVATAADTLVTK.F
	5.2745	1825.866	1825.864	0.9	8471844	K.YSEEGVYNVQYSFIK.E
	3.2337	1229.711	1229.71	1	5423763	R.VLSTDVDVILR.K
sp|P14618|KPYM_HUMAN						
	3.1814	1197.648	1197.647	0.6	1236343	R.LDIDSPPITAR.N
	3.3028	1359.706	1359.705	0.4	1671276	R.NTGIICTIGPASR.S
	5.3952	2465.292	2465.292	-0.1	5101248	R.TATESFASDPILYRPVAVALDTK.G
	4.089	1468.681	1468.678	2.2	1404918	K.CDENILWLDYK.N
	4.4347	1779.879	1779.876	1.9	2282091	K.GADFLVTEVENGGSLGSK.K
	5.0994	2175.119	2175.118	0.3	2544758	R.LAPITSDPTEATAVGAVEASFK.C
	3.0965	1221.633	1221.633	-0.2	940449.7	K.CCSGAIIVLTK.S
	5.229	1642.773	1642.771	1.6	2421633	K.DPVQEAWAEDVDLR.V
sp|P68366-2|TBA4A_HUMAN						
sp|P68366|TBA4A_HUMAN						
	5.5708	2067.898	2067.897	0.9	1821368	K.TIGGGDDSFTTFFCETGAGK.H
	3.8765	1715.924	1715.921	1.6	6052356	R.AVFVDLEPTVIDEIR.N
	4.1211	1487.882	1487.879	2	11154716	R.LISQIVSSITASLR.F
	3.9394	1584.75	1584.752	-1.1	4055450	R.SIQFVDWCPTGFK.V
	4.5754	1824.988	1824.985	1.3	6671799	K.VGINYQPPTVVPGGDLAK.V
	6.3456	2330.022	2330.018	1.5	3624178	R.AFVHWYVGEGMEEGEFSEAR.E
sp|P81605-2|DCD_HUMAN						
	3.9874	1217.674	1217.674	0.2	15259022	R.SSLLEKGLDGAK.K
	3.114	1161.563	1161.563	-0.2	13831352	K.DAVEDLESVGK.G
*	3.2666	1689.793	1689.793	0.4	516016.7	K.DAVEDLESVGKGGEER.L
sp|O60220|TIM8A_HUMAN						

*	2.459	1354.589	1354.588	1.1	875881.4	R.AEACFVNCVER.F
*	2.9752	1353.717	1353.716	1	1818682	R.FIDTSQFILNR.L
sp|P02545|LMNA_HUMAN						
	2.4426	1131.526	1131.528	-1.1	3447966	K.EDLQELNDR.L
	2.9216	1148.581	1148.579	1.7	10084664	R.ITESEEVVSR.E
	3.0732	1165.55	1165.548	1.3	1772269	K.AAYEAELGDAR.K
	2.6262	1182.613	1182.611	1.3	1438818	R.TLEGELHDLR.G
	2.9269	1028.573	1028.574	-0.5	4022607	R.LADALQELR.A
	2.4342	1752.866	1752.862	2	546239.5	R.NSNLVGAAHEELQQSR.I
	4.5828	1752.864	1752.862	0.8	2695606	R.NSNLVGAAHEELQQSR.I
	5.0139	1893.928	1893.926	0.8	2034302	R.MQQQLDEYQELLDIK.L
	3.7264	1536.674	1536.675	-0.2	709500.9	K.SNEDQSMGNWQIK.R
	3.054	1291.628	1291.628	0.6	804670.8	R.QNGDDPLLTYR.F
	3.9556	1491.748	1491.747	0.8	1189803	R.TALINSTGEEVAMR.K
	3.1864	1406.641	1406.64	0.3	1204440	R.TVLCGTCGQPADK.A
	4.2626	1566.75	1566.751	-0.3	1472439	R.SVGGSGGGSFGDNLVTR.S
sp|Q9BQE3|TBA1C_HUMAN						
	7.2957	2007.895	2007.893	0.8	16446812	K.TIGGGDDSFNTFFSETGAGK.H
	4.414	1701.907	1701.906	0.9	17363270	R.AVFVDLEPTVIDEVR.T
	4.1211	1487.882	1487.879	2	11154716	R.LISQIVSSITASLR.F
	3.5498	1598.767	1598.767	-0.2	3130021	R.TIQFVDWCPTGFK.V
	4.5754	1824.988	1824.985	1.3	6671799	K.VGINYQPPTVVPGGDLAK.V
	6.3456	2330.022	2330.018	1.5	3624178	R.AFVHWYVGEGMEEGEFSEAR.E
sp|P55327-2|TPD52_HUMAN						
sp|P55327|TPD52_HUMAN						
sp|P55327-7|TPD52_HUMAN						
sp|P55327-6|TPD52_HUMAN						
sp|P55327-5|TPD52_HUMAN						
sp|P55327-4|TPD52_HUMAN						
sp|P55327-3|TPD52_HUMAN						
	4.3988	1657.904	1657.901	1.8	2178438	K.VEEEIQTLSQVLAAK.E
	3.2399	1326.632	1326.632	-0.6	1364029	K.GWQDVTATSAYK.K
	2.7757	1324.711	1324.711	0.1	648413.5	K.ASAAFSSVGSVITK.K
sp|P68104|EF1A1_HUMAN						
sp|Q5VTE0|EF1A3_HUMAN						
	4.6881	1588.884	1588.881	2.5	18198744	K.THINIVVIGHVDSGK.S
	6.1941	1588.881	1588.881	0.5	15242918	K.THINIVVIGHVDSGK.S
	3.4526	1404.728	1404.727	0.8	4727275	K.YYVTIIDAPGHR.D
	3.6936	1314.743	1314.742	1.3	4277484	R.EHALLAYTLGVK.Q
	3.5614	1314.742	1314.742	0.1	7016242	R.EHALLAYTLGVK.Q
	5.3714	3021.569	3021.567	0.6	9010335	K.DGNASGTTLLEALDCILPPTRPTDKPLR.L
	6.4263	3021.571	3021.567	1.2	15670333	K.DGNASGTTLLEALDCILPPTRPTDKPLR.L
	2.8263	1025.61	1025.61	0	25613252	K.IGGIGTVPVGR.V
	5.927	2515.387	2515.384	1.1	7166693	R.VETGVLKPGMVVTFAPVNVTTEVK.S
	4.8017	1788.958	1788.956	0.7	3775204	K.PGMVVTFAPVNVTTEVK.S
sp|P39019|RS19_HUMAN						
*	2.4045	1072.567	1072.567	-0.1	1122648	K.VPEWVDTVK.L
*	3.3183	1703.772	1703.77	1.3	17889476	K.ELAPYDENWFYTR.A
*	3.5278	1126.695	1126.694	0.5	6426624	R.RVLQALEGLK.M
sp|Q06830|PRDX1_HUMAN						
*	2.7346	1107.605	1107.604	0.3	1981678	R.TIAQDYGVLK.A
	2.9601	1211.674	1211.674	0.2	7476863	R.QITVNDLPVGR.S
*	5.1983	2406.193	2406.187	2.4	2107184	K.HGEVCPAGWKPGSDTIKPDVQK.S
sp|P17066|HSP76_HUMAN						
	3.052	1487.703	1487.701	0.9	3841529	R.TTPSYVAFTDTER.L
	3.8144	1305.67	1305.669	1.1	7238788	K.ETAEAYLGQPVK.H
*	2.8918	1169.664	1169.664	0.6	8105586	K.DAGAIAGLNVLR.I
	4.6927	1687.906	1687.901	2.6	29593658	R.IINEPTAAAIAYGLDR.R
	4.9763	1675.733	1675.731	1.6	6645407	K.ATAGDTHLGGEDFDNR.L
	3.4423	1675.731	1675.731	0.3	21347504	K.ATAGDTHLGGEDFDNR.L
	3.3602	1542.74	1542.737	2.1	3356229	R.ARFEELCSDLFR.S
	3.1491	1315.6	1315.599	0.9	27896786	R.FEELCSDLFR.S
	3.0309	1183.648	1183.647	0.7	2801654	R.FELSGIPPAPR.G
*	3.1508	1679.876	1679.875	0.6	629366.7	R.EVLAWLEHNQLAEK.E
*	3.8666	1721.808	1720.807	-1.6	2850781	R.LYGGPGVPGGSSCGTQAR.Q
*	3.1268	1456.682	1456.68	1.1	2437722	R.QGDPSTGPIIEEVD.-

sp|P62140|PP1B_HUMAN						
	4.7804	1551.848	1551.845	1.8	1419764	K.QSLETICLLLAYK.I
	4.3034	1439.805	1439.805	0.3	4136497	K.IKYPENFFLLR.G
	3.3963	2113.997	2113.993	1.6	1578651	K.TFTDCFNCLPIAAIVDEK.I
*	2.8009	1313.675	1313.674	1.3	564336.6	R.GVSFTFGADVVSK.F
	2.6365	1639.777	1639.775	1.1	324285	R.AHQVVEDGYEFFAK.R
sp|P31942-3|HNRH3_HUMAN						
sp|P31942|HNRH3_HUMAN						
	3.4547	1271.629	1271.627	1.5	4371840	R.STGEAFVQFASK.E
	3.5296	3435.311	3435.312	-0.3	930897	R.RGGDGYDGGYGGFDDYGGYNNYGYGNDGFDDR.M
	5.1037	1918.972	1918.966	3.1	4250169	R.ATENDIANFFSPLNPIR.V
	4.248	1918.971	1918.966	2.5	3494650	R.ATENDIANFFSPLNPIR.V
sp|Q9Y2S7|PDIP2_HUMAN						
*	3.8681	1316.748	1316.746	1.8	9125720	K.VLETVGVFEVPK.Q
*	4.009	1830.916	1830.917	-0.5	4805130	K.YETGQLFLHSIFGYR.G
*	3.7548	1479.762	1479.759	2	1090365	K.THTYYQVLIDAR.D
*	4.2005	1502.75	1502.75	0.3	1610816	K.NHPWLELSDVHR.E
*	3.9347	1400.738	1400.738	0.1	4585629	R.LENLDSDVVQLR.E
*	3.5357	1322.733	1322.731	0.8	5425436	R.IFSLSGTLETVR.G
sp|P62136-2|PP1A_HUMAN						
sp|P62136|PP1A_HUMAN						
	2.6333	1215.634	1215.633	0.7	1421299	K.NVQLTENEIR.G
	4.7804	1551.848	1551.845	1.8	1419764	K.QSLETICLLLAYK.I
	4.3034	1439.805	1439.805	0.3	4136497	K.IKYPENFFLLR.G
	3.3963	2113.997	2113.993	1.6	1578651	K.TFTDCFNCLPIAAIVDEK.I
	2.6365	1639.777	1639.775	1.1	324285	R.AHQVVEDGYEFFAK.R
sp|P63244|RACK1_HUMAN						
*	2.5286	1192.559	1192.559	0	3848226	R.DETNYGIPQR.A
*	2.9399	1264.653	1264.653	0.2	3818065	R.LWDLTTGTTTR.R
*	3.1984	1309.64	1309.638	1.3	1918236	K.DVLSVAFSSDNR.Q
*	4.1306	1981.873	1981.871	1.2	1890215	K.YTVQDESHSEWVSCVR.F
*	2.8394	1366.684	1366.682	1.1	2513120	R.YWLCAATGPSIK.I
sp|P12273|PIP_HUMAN						
*	2.7262	1815.973	1814.969	0.4	1288292	K.TYLISSIPLQGAFNYK.Y
*	3.1959	1283.77	1283.768	1.1	1578588	R.TVQIAAVVDVIR.E
sp|Q9UFN0|NPS3A_HUMAN						
*	3.2447	1337.618	1337.616	1.6	9375959	R.QYDGIFYEFR.S
*	4.9397	1652.8	1652.799	1	4160429	K.QESEITYLVPWCK.L
*	3.8617	1215.617	1215.616	0.6	740785.4	K.PGGPALWGDAFK.R
*	2.7269	1346.777	1346.775	1.5	1230077	N.MLLIPTSFSPLK.-
sp|P31949|S10AB_HUMAN						
*	3.0435	1307.703	1307.703	0.4	1032623	R.CIESLIAVFQK.Y
*	2.3185	1060.495	1060.495	0.8	3172354	K.DGYNYTLSK.T
sp|O43813|LANC1_HUMAN						
*	2.8538	1256.592	1256.591	1.1	4650929	K.SLAEGYFDAAGR.L
*	5.1454	2203.163	2203.158	2.1	869957.2	R.SITFLCGDAGPLAVAAVLYHK.M
*	4.134	1413.818	1413.814	2.6	17096432	R.IGYIYALLFVNK.N
*	4.6986	1886.023	1886.02	1.2	3864418	K.LHSLVKPSVDYVCQLK.F
*	3.0276	1593.712	1593.711	0.5	410283.4	K.FPSGNYPPCIGDNR.D
sp|Q01105-2|SET_HUMAN						
sp|Q01105|SET_HUMAN						
sp|Q01105-4|SET_HUMAN						
sp|Q01105-3|SET_HUMAN						
	5.5919	2195.023	2195.021	1	9049249	K.EQQEAIEHIDEVQNEIDR.L
	3.5411	1273.665	1273.663	1	12637962	R.LNEQASEEILK.V
	3.3807	1208.605	1208.605	0.4	3628711	R.VEVTEFEDIK.S
	4.1016	1446.655	1446.65	3.6	2952901	K.EFHLNESGDPSSK.S
sp|P35268|RL22_HUMAN						
*	2.7541	1242.682	1242.68	1.3	3103591	K.AGNLGGGVVTIER.S
*	3.0962	1207.656	1207.657	-0.7	2115554	K.ITVTSEVPFSK.R
sp|P55795|HNRH2_HUMAN						
	3.4605	1684.771	1684.767	2	1078318	K.HTGPNSPDTANDGFVR.L
	6.9471	1841.896	1841.892	2.2	13753355	R.STGEAFVQFASQEIAEK.A
*	2.8151	2816.131	2816.124	2.3	227907.4	R.RGAYGGGYGGYDDYGGYNDGYGFGSDR.F
	2.2615	1601.651	1601.647	2.5	361833.2	R.DLNYCFSGMSDHR.Y
	2.7636	1092.578	1092.58	-1.2	8628529	R.VHIEIGPDGR.V

sp|P68371|TBB4B_HUMAN						
*	2.6557	1329.655	1328.648	2.3	3470842	R.INVYYNEATGGK.Y
	8.7496	2798.348	2798.344	1.7	10732236	R.SGPFGQIFRPDNFVFGQSGAGNNWAK.G
	2.4645	1039.594	1039.594	0.2	4975216	R.YLTVAAVFR.G
	3.5734	1446.69	1446.689	0.4	4134744	K.EVDEQMLNVQNK.N
	4.9284	1696.835	1696.833	1.2	21538244	K.NSSYFVEWIPNNVK.T
	2.3318	1028.519	1028.519	-0.1	1062831	K.TAVCDIPPR.G
sp|P37108|SRP14_HUMAN						
*	5.8015	1808.951	1808.946	2.9	7403549	-.MVLLESEQFLTELTR.L
*	2.9034	1068.594	1068.594	0.6	3910120	R.TSGSVYITLK.K
sp|P52597|HNRPF_HUMAN						
*	5.7872	1709.788	1709.786	0.9	2778915	R.QSGEAFVELGSEDDVK.M
*	3.6795	1630.721	1630.721	0.4	1321729	K.HSGPNSADSANDGFVR.L
*	4.5535	1630.722	1630.721	0.7	8420602	K.HSGPNSADSANDGFVR.L
*	6.1906	1867.952	1867.944	4.5	3782724	K.ITGEAFVQFASQELAEK.A
	5.0126	1996.981	1996.976	2.3	10218354	K.ATENDIYNFFSPLNPVR.V
	5.1728	1996.982	1996.976	3.1	15758678	K.ATENDIYNFFSPLNPVR.V
	2.7636	1092.578	1092.58	-1.2	8628529	R.VHIEIGPDGR.V
sp|Q9HCC0|MCCB_HUMAN						
	2.7137	1706.848	1706.845	1.7	212593.9	R.VSGVECMIIANDATVK.G
*	3.8752	1340.795	1340.794	1.3	3038858	K.QGTIFLAGPPLVK.A
	3.5164	1244.649	1244.649	0.4	1378651	K.MVAAVACAQVPK.I
	3.5651	1746.928	1746.927	0.4	326696.3	K.QFSSADEAALKEPIIK.K
	4.3599	1548.661	1548.66	0.5	1866138	K.FEEEGNPYYSSAR.V
	4.1523	1472.705	1472.702	2.2	5820624	R.VWDDGIIDPADTR.L
	3.3123	1743.015	1743.005	5.5	694507.8	R.LVLGLSFSAALNAPIEK.T
sp|P24844-2|MYL9_HUMAN						
sp|P24844|MYL9_HUMAN						
	2.5777	1433.591	1433.589	1.6	746040	R.FTDEEVDEMYR.E
	2.7809	1246.587	1246.585	1.8	829577.9	K.GNFNYVEFTR.I
sp|P61204|ARF3_HUMAN						
sp|P84085|ARF5_HUMAN						
sp|P84077|ARF1_HUMAN						
	3.0147	2323.247	2323.243	1.7	1147328	K.LGEIVTTIPTIGFNVETVEYK.N
	5.0843	2323.247	2323.243	1.8	2198221	K.LGEIVTTIPTIGFNVETVEYK.N
	3.0289	1089.631	1089.63	0.7	1600407	R.DAVLLVFANK.Q
sp|Q9BPW8|NIPS1_HUMAN						
*	2.2102	1010.433	1010.433	0.2	3100648	K.DNEGSWFR.S
*	5.7417	2018.018	2018.013	2.2	2649405	R.NQLLLEFSFWNEPQPR.M
*	2.8396	1092.55	1092.551	-0.7	2634370	R.MGPNIYELR.T
*	4.8163	1866.952	1866.95	1.1	6513681	K.RGWDENVYYTVPLVR.H
*	3.9371	1710.851	1710.849	1.7	3302402	R.GWDENVYYTVPLVR.H
sp|P43243|MATR3_HUMAN						
*	5.1648	1792.886	1792.882	2	3243669	R.GDADQASNILASFGLSAR.D
*	3.6492	1479.701	1479.696	3.4	1081000	R.DLDELSRYPEDK.I
*	3.8478	1619.977	1619.973	2.5	11140004	K.ITPENLPQILLQLK.R
*	4.9316	1935.014	1935.008	3.2	1011145	K.RGAPPSSNIEDFHGLLPK.G
	5.8906	1914.932	1914.929	1.5	5581837	K.IEELDQENEAALENGIK.N
	4.0794	2146.868	2146.864	1.7	726087.7	K.NEENTEPGAESSENADDPNK.D
	2.8106	1286.554	1286.554	0.1	3590088	K.DDYTIPDEYR.I
	4.2411	1969.077	1969.079	-1.2	5202315	R.IGPYQPNVPVGIDYVIPK.T
	3.7074	1573.76	1573.757	2.2	2651879	K.LCSLFYTNEEVAK.N
sp|P34931|HS71L_HUMAN						
	3.052	1487.703	1487.701	0.9	3841529	R.TTPSYVAFTDTER.L
	3.129	1614.81	1614.808	0.8	5676068	K.AFYPEEISSMVLTK.L
	3.492	1197.697	1197.695	1.3	47052752	K.DAGVIAGLNVLR.I
	4.8208	1659.899	1659.895	2.1	12702546	R.IINEPTAAAIAYGLDK.G
	4.9763	1675.733	1675.731	1.6	6645407	K.ATAGDTHLGGEDFDNR.L
	3.4423	1675.731	1675.731	0.3	21347504	K.ATAGDTHLGGEDFDNR.L
	6.4454	2786.363	2786.363	0	4629315	K.QTQIFTTYSDNQPGVLIQVYEGER.A
	5.6017	2786.368	2786.363	1.9	31202058	K.QTQIFTTYSDNQPGVLIQVYEGER.A
	3.2484	1287.605	1287.604	0.7	7262432	K.NALESYAFNMK.S
sp|P04083|ANXA1_HUMAN						
*	3.8821	1387.771	1387.768	2	1621752	K.GVDEATIIDILTK.R
*	2.6437	1262.602	1262.601	0.6	1175831	K.TPAQFDADELR.A
*	4.7085	1702.89	1702.886	2.5	2147545	K.GLGTDEDTLIEILASR.T

*	3.1153	1739.738	1739.736	1.7	335380.9	R.SEDFGVNEDLADSDAR.A
sp|P07951-2|TPM2_HUMAN						
sp|P07951|TPM2_HUMAN						
	3.8221	1343.68	1343.68	0	4935372	K.QLEEEQQALQK.K
	3.5995	1332.642	1332.639	2	2355590	K.ATDAEADVASLNR.R
	4.1378	1399.755	1399.754	0.6	5117270	R.RIQLVEEELDR.A
	3.1417	1243.653	1243.653	0.5	8889076	R.IQLVEEELDR.A
	2.9113	1170.674	1170.673	0.5	10834050	K.LVILEGELER.S
sp|Q05682-2|CALD1_HUMAN						
sp|Q05682|CALD1_HUMAN						
sp|Q05682-6|CALD1_HUMAN						
sp|Q05682-5|CALD1_HUMAN						
sp|Q05682-4|CALD1_HUMAN						
sp|Q05682-3|CALD1_HUMAN						
	5.4768	2972.362	2972.36	0.4	1133910	K.QEEESLGQVTDQVEVNAQNSVPDEEAK.T
	5.8346	2097.958	2097.957	0.3	791205.7	K.TTTTNTQVEGDDEAAFLER.L
	4.3391	1748.872	1748.87	1.2	8745801	K.EFDPTITDASLSLPSR.R
	2.911	1341.645	1341.642	2.5	3261997	R.YEIEETETVTK.S
	3.4776	1378.734	1378.733	1.2	477172.2	K.PAASDLPVPAEGVR.N
sp|P50454|SERPH_HUMAN						
*	4.8186	1659.804	1659.801	1.3	10304054	R.LYGPSSVSFADDFVR.S
*	3.1289	1380.595	1380.595	-0.1	1128311	R.TGLYNYYDDEK.E
*	2.8788	1637.731	1637.733	-1.2	576291.1	R.TGLYNYYDDEKEK.L
*	3.8383	1224.658	1224.658	0.1	4738733	K.GVVEVTHDLQK.H
*	4.3788	1337.744	1337.742	1.1	2117144	K.HLAGLGLTEAIDK.N
*	3.2263	1293.682	1293.68	1.4	2468635	R.DTQSGSLLFIGR.L
sp|O43707|ACTN4_HUMAN						
	3.4061	1507.703	1507.702	0.5	504201.6	K.AGTQIENIDEDFR.D
*	2.8864	1515.812	1514.806	2	556504.2	K.LVSIGAEEIVDGNAK.M
	3.9812	1537.776	1537.774	0.9	1007323	R.FAIQDISVEETSAK.E
*	2.5668	1775.848	1775.845	1.6	328564.5	K.DDPVTNLNNAFEVAEK.Y
	3.0874	1215.675	1215.673	1.5	1055762	K.LASDLLEWIR.R
	3.6868	1608.807	1608.805	1.1	590788	K.CQLEINFNTLQTK.L
	3.2177	1421.708	1421.706	1.2	990029.9	K.GYEEWLLNEIR.R
	4.5245	1920.009	1920.007	0.9	653762.3	K.LSGSNPYTTVTPQIINSK.W
	3.9586	1386.776	1386.774	1.4	4515790	R.VGWEQLLTTIAR.T
	5.2196	1741.814	1741.813	0.6	1674497	R.ETTDTDTADQVIASFK.V
sp|P27797|CALR_HUMAN						
*	2.7148	1219.705	1219.705	0.7	1350990	K.GQTLVVQFTVK.H
*	4.76	2760.267	2760.263	1.2	3181565	K.IDDPTDSKPEDWDKPEHIPDPDAK.K
*	6.0574	2708.04	2708.034	2.3	707026.6	K.DEDEEDEEDKEEDEEEDVPGQAK.D
*	4.0869	1474.621	1474.618	2	3580333	K.EEDEEEDVPGQAK.D
*	2.7915	1831.774	1831.772	1.4	396110.7	K.EEDEEEDVPGQAKDEL.-
sp|P25398|RS12_HUMAN						
*	2.9446	1061.545	1061.545	0.6	5416996	K.LGEWVGLCK.I
*	3.5519	1106.571	1106.57	1.1	3682208	K.VVGCSCVVVK.D
sp|P67936|TPM4_HUMAN						
*	3.9315	1614.773	1614.772	0.5	747945.9	K.IQALQQQADEAEDR.A
	4.1378	1399.755	1399.754	0.6	5117270	R.RIQLVEEELDR.A
	3.1417	1243.653	1243.653	0.5	8889076	R.IQLVEEELDR.A
	2.9113	1170.674	1170.673	0.5	10834050	K.LVILEGELER.A
sp|P10909-2|CLUS_HUMAN						
sp|P10909|CLUS_HUMAN						
sp|P10909-5|CLUS_HUMAN						
sp|P10909-4|CLUS_HUMAN						
sp|P10909-3|CLUS_HUMAN						
	3.7253	1393.697	1393.696	1	6935543	R.ASSIIDELFQDR.F
	5.3072	1762.829	1762.828	0.8	3562259	R.EILSVDCSTNNPSQAK.L
	3.1315	1288.638	1288.638	0.2	2871688	R.ELDESLQVAER.L
	3.5229	1683.838	1683.834	2.5	406868.6	R.LANLTQGEDQYYLR.V
	5.369	1874.992	1873.991	-1.1	5769705	K.LFDSDPITVTVPVEVSR.K
sp|P46783|RS10_HUMAN						
*	3.0711	1109.664	1109.661	3.3	27256534	R.IAIYELLFK.E
	4.464	1441.673	1441.671	1.4	3311922	K.AEAGAGSATEFQFR.G
sp|Q9BWF3|RBM4_HUMAN						
	2.7636	1221.591	1221.59	0.8	2270173	K.NYGFVHIEDK.T

	4.779	1697.811	1697.809	0.9	1582229	K.FEEYGPVIECDIVK.D
	2.6905	1108.529	1108.527	1.7	2578597	R.GLDNTEFQGK.R
	5.3195	1855.883	1855.882	0.6	1476239	R.VADLTEQYNEQYGAVR.T
sp|Q13162|PRDX4_HUMAN						
*	4.316	1779.862	1779.859	2	1762901	K.PAPYWEGTAVIDGEFK.E
*	2.7837	1225.691	1225.69	0.8	2043931	R.QITLNDLPVGR.S
*	2.7762	1212.627	1212.626	0.5	2217605	R.LVQAFQYTDK.H
sp|P02794|FRIH_HUMAN						
*	2.5883	1153.597	1153.596	0.6	3453339	K.ELGDHVTNLR.K
*	4.8514	1627.769	1627.767	1.3	2657869	K.MGAPESGLAEYLFDK.H
sp|P52272-2|HNRPM_HUMAN						
sp|P52272|HNRPM_HUMAN						
	2.3708	1868.834	1867.849	-9.8	530061.7	K.MEEESGAPGVPSGNGAPGPK.G
	3.3399	1264.695	1264.694	1	7273742	R.AFITNIPFDVK.W
	4.7627	1752.878	1752.872	3.1	1598386	K.VGEVTYVELLMDAEGK.S
	4.1939	1426.759	1426.758	0.9	1610967	R.LGSTVFVANLDYK.V
	2.4745	1193.636	1193.635	1	937244.4	K.EVFSMAGVVVR.A
	4.63	2034.955	2034.953	1.1	1441958	R.GNFGGSFAGSFGGAGGHAPGVAR.K
sp|P67809|YBOX1_HUMAN						
	2.1479	940.4638	940.4636	0.3	1433689	R.NGYGFINR.N
	5.0937	1795.826	1795.823	1.3	1604512	R.SVGDGETVEFDVVEGEK.G
*	5.0872	1695.869	1695.866	1.4	1373909	K.GAEAANVTGPGGVPVQGSK.Y
sp|P60842|IF4A1_HUMAN						
	3.3522	1394.696	1394.691	3.2	1143207	K.GYDVIAQAQSGTGK.T
	2.809	1140.674	1140.674	0.3	1558182	K.ATQALVLAPTR.E
	2.5362	1114.684	1114.683	1.1	2933674	R.VLITTDLLAR.G
	4.2956	2145.138	2144.135	0.3	548721.3	R.GIDVQQVSLVINYDLPTNR.E
sp|P05388-2|RLA0_HUMAN						
sp|Q8NHW5|RLA0L_HUMAN						
sp|P05388|RLA0_HUMAN						
	3.1358	1266.615	1266.615	0.5	2118415	K.CFIVGADNVGSK.Q
	2.9062	2181.101	2180.102	-1.7	382244.6	R.AGAIAPCEVTVPAQNTGLGPEK.T
sp|P09651-2|ROA1_HUMAN						
sp|Q32P51|RA1L2_HUMAN						
sp|P09651|ROA1_HUMAN						
sp|P09651-3|ROA1_HUMAN						
	4.7234	1784.911	1784.907	2.6	7339852	K.LFIGGLSFETTDESLR.S
	3.1179	1218.641	1218.64	1	3456976	K.IEVIEIMTDR.G
	5.9383	1855.863	1855.861	0.9	1819517	K.RGFAFVTFDDHDSVDK.I
sp|P63104|1433Z_HUMAN						
*	4.5538	1548.714	1548.714	-0.1	1089792	K.SVTEQGAELSNEER.N
	4.8531	2040.989	2040.987	0.8	380999.4	K.GIVDQSQQAYQEAFEISK.K
sp|P24534|EF1B_HUMAN						
*	2.5413	1771.77	1770.767	0	320307.5	K.YGPADVEDTTGSGATDSK.D
	2.9206	1346.663	1346.662	0.5	2010671	K.LQIQCVVEDDK.V
sp|Q96DH6-2|MSI2H_HUMAN						
sp|Q96DH6|MSI2H_HUMAN						
sp|Q96DH6-3|MSI2H_HUMAN						
	4.1875	1647.898	1647.895	1.6	1276481	K.IFVGGLSANTVVEDVK.Q
	4.4623	1716.814	1716.812	1.3	2928471	R.GFGFVTFENEDVVEK.V
sp|P62913-2|RL11_HUMAN						
sp|P62913|RL11_HUMAN						
	2.9376	1492.69	1492.688	1.1	1278564	K.LCLNICVGESGDR.L
	2.529	975.5507	975.551	-0.3	2160925	K.YDGIILPGK.-
sp|P09493-10|TPM1_HUMAN						
sp|P09493|TPM1_HUMAN						
sp|P09493-9|TPM1_HUMAN						
sp|P09493-4|TPM1_HUMAN						
sp|P09493-3|TPM1_HUMAN						
	3.8823	1301.696	1301.695	0.8	3937174	K.QLEDELVSLQK.K
	3.5995	1332.642	1332.639	2	2355590	K.ATDAEADVASLNR.R
	4.1378	1399.755	1399.754	0.6	5117270	R.RIQLVEEELDR.A
	3.1417	1243.653	1243.653	0.5	8889076	R.IQLVEEELDR.A
sp|O75340-2|PDCD6_HUMAN						
sp|O75340|PDCD6_HUMAN						
	3.5135	1441.712	1441.711	0.4	1890108	K.AGVNFSEFTGVWK.Y

	2.7354	1341.66	1341.659	1.4	2701336	K.YITDWQNVFR.T
sp|P49748-2|ACADV_HUMAN						
sp|P49748|ACADV_HUMAN						
sp|P49748-3|ACADV_HUMAN						
	1.9565	848.524	848.524	0	2273330	K.GILLFGTK.A
	2.4305	934.5356	934.5356	-0.1	3252113	K.ITAFVVER.G
	3.8841	1412.682	1412.68	1.4	1820454	K.ASNTAEVFFDGVR.V
	5.4332	1518.78	1518.78	0.3	2407860	R.VPSENVLGEVGSGFK.V
	3.5074	1316.733	1316.732	0.8	1225767	K.GIVNEQFLLQR.L
	4.4434	2021.94	2021.939	0.7	675819.9	R.EGMAALQSDPWQQELYR.N
sp|P67936-2|TPM4_HUMAN						
*	2.829	1353.703	1353.701	1.3	566142.4	K.QVEEELTHLQK.K
	4.1378	1399.755	1399.754	0.6	5117270	R.RIQLVEEELDR.A
	3.1417	1243.653	1243.653	0.5	8889076	R.IQLVEEELDR.A
	2.9113	1170.674	1170.673	0.5	10834050	K.LVILEGELER.A
sp|P07910-2|HNRPC_HUMAN						
sp|P07910|HNRPC_HUMAN						
sp|P07910-4|HNRPC_HUMAN						
	3.863	1316.795	1316.794	1.4	5572444	R.VFIGNLNTLVVK.K
	3.4307	1329.661	1329.659	1.7	1479687	K.GFAFVQYVNER.N
	2.9326	1159.621	1159.621	0.5	2873479	K.VDSLLENLEK.I
sp|P08238|HS90B_HUMAN						
	2.1166	1039.497	1039.494	2.1	2320536	R.YESLTDPSK.L
	2.891	1194.65	1194.648	1.7	1570323	K.IDIIPNPQER.T
	3.479	1242.707	1242.705	1.5	1230459	K.ADLINNLGTIAK.S
	3.1683	1311.574	1311.57	2.8	2232794	K.EDQTEYLEER.R
*	4.5726	1847.797	1847.797	0.1	782628	R.NPDDITQEEYGEFYK.S
	2.8416	1527.744	1527.744	0.3	325131.8	K.SLTNDWEDHLAVK.H
	2.6443	1416.642	1416.638	3.3	967321.3	K.EGLELPEDEEEK.K
sp|P61978-2|HNRPK_HUMAN						
sp|P61978|HNRPK_HUMAN						
sp|P61978-3|HNRPK_HUMAN						
	3.8455	1780.799	1780.798	0.4	2430433	R.TDYNASVSVPDSSGPER.I
	2.5935	1098.453	1098.452	1	974217.7	K.GSDFDCELR.L
	5.2658	1518.938	1518.937	0.6	3691813	R.LLIHQSLAGGIIGVK.G
	3.0105	1259.576	1259.575	1.2	2933885	K.IDEPLEGSEDR.I
sp|P31689-2|DNJA1_HUMAN						
sp|P31689|DNJA1_HUMAN						
	4.3103	1451.739	1451.738	1	2418883	K.QISQAYEVLSDAK.K
	3.932	1392.822	1392.821	0.8	4205957	R.TIVITSHPGQIVK.H
	2.9429	1351.651	1351.65	0.9	936368.4	K.CVLNEGMPIYR.R
sp|Q15019-2|SEPT2_HUMAN						
sp|Q15019|SEPT2_HUMAN						
sp|Q15019-3|SEPT2_HUMAN						
	3.7461	1603.82	1603.817	1.8	622832.7	R.TVQIEASTVEIEER.G
	3.6531	1513.755	1513.753	1.2	1043097	K.TIISYIDEQFER.Y
	3.7466	1759.961	1759.959	1.5	1875826	K.ASIPFSVVGSNQLIEAK.G
sp|Q8NC51-3|PAIRB_HUMAN						
sp|Q8NC51-4|PAIRB_HUMAN						
	4.5834	3378.476	3378.473	1	1073064	K.DELTDLDQSNVTEETPEGEEHHPVADTENK.E
	2.999	1515.72	1515.717	1.9	604632.6	K.ENEVEEVKEEGPK.E
sp|P25705-2|ATPA_HUMAN						
sp|P25705|ATPA_HUMAN						
	5.3576	1575.787	1575.786	0.5	2346295	R.ILGADTSVDLEETGR.V
	4.0447	1624.891	1624.89	0.5	3585855	R.TGAIVDVPVGEELLGR.V
	4.153	1316.743	1316.742	0.6	2266932	K.TSIAIDTIINQK.R
	3.9393	1287.695	1287.694	0.8	1339579	K.HALIIYDDLSK.Q
sp|P22626-2|ROA2_HUMAN						
sp|P22626|ROA2_HUMAN						
	3.2565	1188.648	1188.647	0.8	8505543	K.IDTIEIITDR.Q
	6.083	1851.868	1851.866	1.2	4158426	K.RGFGFVTFDDHDPVDK.I
	2.1883	1013.444	1013.444	0.2	6692290	R.GGNFGFGDSR.G
sp|Q99729-2|ROAA_HUMAN						
sp|Q99729-3|ROAA_HUMAN						
	4.2208	1503.771	1503.769	1.4	1588122	K.IFVGGLNPEATEEK.I
	2.0738	958.5397	958.5397	0.1	1827372	R.GFVFITFK.E

	3.6411	1499.688	1499.687	0.5	1600413	K.EVYQQQQYGSGGR.G
sp|Q9Y3D9|RT23_HUMAN						
*	2.5055	1108.601	1108.6	0.8	906802.3	R.LETVGSIFSR.T
*	2.5604	1212.606	1212.605	0.7	1137623	R.AFDLFNPNFK.S
sp|P60709|ACTB_HUMAN						
sp|P63261|ACTG_HUMAN						
	2.3432	1132.527	1132.527	-0.1	3317449	R.GYSFTTTAER.E
	4.2873	1790.893	1790.892	0.3	15128025	K.SYELPDGQVITIGNER.F
	4.0186	1516.704	1516.703	0.9	1507194	K.QEYDESGPSIVHR.K
sp|Q15366-2|PCBP2_HUMAN						
sp|Q15366|PCBP2_HUMAN						
sp|Q15366-8|PCBP2_HUMAN						
sp|Q15366-7|PCBP2_HUMAN						
sp|Q15366-6|PCBP2_HUMAN						
sp|Q15366-5|PCBP2_HUMAN						
sp|Q15366-4|PCBP2_HUMAN						
sp|Q15366-3|PCBP2_HUMAN						
	3.254	1288.598	1288.595	2.5	14071398	R.INISEGNCPER.I
	3.3515	1712.965	1712.962	2.1	2701401	R.AITIAGIPQSIIECVK.Q
	2.6785	1158.576	1158.575	1.2	2975010	K.IANPVEGSTDR.Q
sp|Q13501-2|SQSTM_HUMAN						
sp|Q13501|SQSTM_HUMAN						
	2.0569	2241.916	2239.913	-1.7	268331.9	R.YKCSVCPDYDLCSVCEGK.G
	7.2369	2239.916	2239.913	1.5	5000403	R.YKCSVCPDYDLCSVCEGK.G
	4.9933	1948.756	1948.754	0.6	1.02E+08	K.CSVCPDYDLCSVCEGK.G
	4.7854	1948.755	1948.754	0.3	99838528	K.CSVCPDYDLCSVCEGK.G
	4.0691	2412.021	2412.02	0.3	4594978	K.CSVCPDYDLCSVCEGKGLHR.G
	4.3303	1671.824	1671.822	0.9	1225673	K.NYDIGAALDTIQYSK.H
sp|P06753-2|TPM3_HUMAN						
sp|P06753-6|TPM3_HUMAN						
sp|P06753-5|TPM3_HUMAN						
sp|P06753-4|TPM3_HUMAN						
sp|P06753-3|TPM3_HUMAN						
	4.0814	1642.804	1642.803	0.4	3023194	K.IQVLQQQADDAEER.A
	4.1378	1399.755	1399.754	0.6	5117270	R.RIQLVEEELDR.A
	3.1417	1243.653	1243.653	0.5	8889076	R.IQLVEEELDR.A
sp|P25685|DNJB1_HUMAN						
*	3.9257	1477.722	1477.717	3.4	3051645	K.EIAEAYDVLSDPR.K
*	4.2758	1605.815	1605.812	2	552404.7	K.EIAEAYDVLSDPRK.R
	3.4007	1228.576	1228.575	1.1	2553936	R.NPFDTFFGQR.N
	3.051	1006.593	1006.593	0.3	2700768	K.VPGEGLPLPK.T
sp|P62258-2|1433E_HUMAN						
sp|P62258|1433E_HUMAN						
	3.7567	1476.745	1476.744	1.1	3228870	K.LICCDILDVLDK.H
	3.0293	1194.6	1194.6	-0.1	3543500	K.EAAENSLVAYK.A
sp|P04406-2|G3P_HUMAN						
sp|P04406|G3P_HUMAN						
	3.3085	1530.797	1530.794	1.4	660182.8	R.VPTANVSVVDLTCR.L
	3.2175	1763.805	1763.802	1.7	686488	K.LISWYDNEFGYSNR.V
sp|O43615|TIM44_HUMAN						
*	3.7088	1420.739	1420.735	2.5	2718770	K.TEMSEVLTEILR.V
*	4.0203	2309.067	2309.065	0.7	1287843	K.DWCYEATYSQLAHPIQQAK.A
*	4.2408	1477.672	1477.671	0.9	1759142	R.DQDELNPYAAWR.L
sp|P49368-2|TCPG_HUMAN						
sp|P49368|TCPG_HUMAN						
	3.3541	1428.754	1428.752	2	1463893	K.IPGGIIEDSCVLR.G
	3.1668	1333.691	1333.689	1.3	1490550	R.TLIQNCGASTIR.L
	2.8779	1254.709	1254.709	0.1	7685931	K.ELGIWEPLAVK.L
	3.1759	1185.72	1185.72	-0.1	1618867	K.TAVETAVLLLR.I
sp|P13804|ETFA_HUMAN						
*	3.1068	1290.673	1290.672	0.5	556081.1	R.LGGEVSCLVAGTK.C
	2.7183	1630.721	1630.719	1	396465.6	R.GTSFDAAATSGGSASSEK.A
sp|P55735-2|SEC13_HUMAN						
sp|P55735|SEC13_HUMAN						
sp|P55735-4|SEC13_HUMAN						
sp|P55735-3|SEC13_HUMAN						

	3.1112	1169.664	1169.664	0.1	797724.7	R.NGGQILIADLR.G
	3.3362	2013.902	2013.901	0.2	682082.5	R.VFIWTCDDASSNTWSPK.L
sp|P11586|C1TC_HUMAN						
*	2.4737	983.6005	983.5996	0.9	879785.5	R.LAILQVGNR.D
*	2.9609	1180.585	1180.585	0.6	569668.5	R.DDSNLYINVK.L
*	3.3496	1536.684	1536.682	1.5	601499.6	R.GDLNDCFIPCTPK.G
*	3.0999	1165.574	1165.574	0	1311209	K.VVGDVAYDEAK.E
*	2.5508	1159.611	1159.611	0.3	910297.5	R.QPSQGPTFGIK.G
*	3.7829	1486.757	1486.754	2.4	2241486	R.LDIDPETITWQR.V
*	2.7841	1651.87	1651.869	0.3	964653.8	R.AAQAPSSFQLLYDLK.L
sp|Q14247|SRC8_HUMAN						
	2.5823	1085.528	1085.526	1.6	859371.6	Q.SAVGFEYQGK.T
*	3.4879	1200.554	1200.553	0.3	1358989	R.QDSAAVGFDYK.E
	2.9061	1874.88	1874.877	1.7	219763.9	K.NASTFEDVTQVSSAYQK.T
sp|Q96IZ0|PAWR_HUMAN						
*	4.2421	1607.815	1607.812	1.7	712606.6	R.DANVSGTLVSSSTLEK.K
*	3.7805	1575.705	1575.702	1.8	549554.2	R.DLDDIEDENEQLK.Q
sp|P12814-2|ACTN1_HUMAN						
sp|P12814|ACTN1_HUMAN						
sp|P12814-4|ACTN1_HUMAN						
sp|P12814-3|ACTN1_HUMAN						
	3.9812	1537.776	1537.774	0.9	1007323	R.FAIQDISVEETSAK.E
	3.0874	1215.675	1215.673	1.5	1055762	K.LASDLLEWIR.R
	3.6868	1608.807	1608.805	1.1	590788	K.CQLEINFNTLQTK.L
	3.2177	1421.708	1421.706	1.2	990029.9	K.GYEEWLLNEIR.R
	4.067	1712.92	1711.915	1	972029.3	K.LLETIDQLYLEYAK.R
	3.9586	1386.776	1386.774	1.4	4515790	R.VGWEQLLTTIAR.T
sp|Q9NQC3-2|RTN4_HUMAN						
*	3.4162	1537.802	1537.801	0.6	798649.3	R.GSSGSVVVDLLYWR.D
	4.2774	1807.937	1807.932	2.5	1326194	R.AYLESEVAISEELVQK.Y
sp|O76003|GLRX3_HUMAN						
*	3.5301	1286.646	1286.648	-1.5	3363344	K.LEAEGVPEVSEK.Y
*	3.8357	1729.861	1729.858	1.2	2051661	K.AYSNWPTYPQLYVK.G
sp|Q9UHD9|UBQL2_HUMAN						
	3.3576	1738.83	1738.828	0.7	1400078	K.EEFAVPENSSVQQFK.E
*	3.7914	1419.784	1419.784	-0.4	1359537	K.SQTDQLVLIFAGK.I
	4.7338	2040.074	2040.072	1.1	668283.9	R.EANLQALIATGGDINAAIER.L
	4.5058	2040.075	2040.072	1.3	1281235	R.EANLQALIATGGDINAAIER.L
sp|Q14103-2|HNRPD_HUMAN						
sp|Q14103|HNRPD_HUMAN						
sp|Q14103-4|HNRPD_HUMAN						
sp|Q14103-3|HNRPD_HUMAN						
	2.3172	1355.669	1355.666	1.5	978641.8	K.MFIGGLSWDTTK.K
	4.0039	1488.759	1488.758	0.7	2882390	K.IFVGGLSPDTPEEK.I
sp|O43175|SERA_HUMAN						
*	3.6651	1298.732	1298.731	0.1	1607652	K.ILQDGGLQVVEK.Q
*	4.6725	1488.731	1488.729	1.3	2996476	R.AGTGVDNVDLEAATR.K
*	3.5768	1345.77	1345.769	1	988446.9	K.GTIQVITQGTSLK.N
sp|Q9UBS4|DJB11_HUMAN						
*	4.4501	1771.841	1771.839	1.5	1384138	K.FQDLGAAYEVLSDSEK.R
*	2.4311	1302.585	1302.585	0.5	1655551	K.QYDTYGEEGLK.D
sp|P58107|EPIPL_HUMAN						
*	4.5358	1884.994	1884.991	1.4	774567.3	R.LSVEEAVAAGVVGGEIQEK.L
*	4.262	2164.085	2164.082	1.5	1159199	K.GFFDPNTHENLTYVQLLR.R
*	3.1363	1618.862	1618.859	1.8	1131045	R.AVPVWDVLASGYVSR.A
*	4.1583	2564.315	2564.31	1.6	4084078	R.LTAIIEEAEEAPGARPQLQDAWR.G
sp|P46060|RAGP1_HUMAN						
*	3.1306	1436.693	1436.69	2	864102.4	K.EIEDFDSLEALR.L
*	4.0722	1718.845	1718.844	0.1	839189.9	K.SSVLIAQQTDTSDPEK.V
*	3.5944	1692.8	1692.798	1.4	376650.1	K.AFNSSSFNSNTFLTR.L
sp|Q13148|TADBP_HUMAN						
*	4.2431	1341.779	1341.778	1	3975581	K.TSDLIVLGLPWK.T
	4.4569	1726.771	1726.768	1.5	1338437	R.FGGNPGGFGNQGGFGNSR.G
sp|P50990-2|TCPQ_HUMAN						
sp|P50990|TCPQ_HUMAN						
	3.5606	1333.749	1333.747	0.9	3328650	K.LFVTNDAATILR.E

	3.8963	1372.744	1372.743	0.5	2557498	K.AIADTGANVVVTGGK.V
	3.2628	1151.593	1150.589	0.1	2004642	K.FAEAFEAIPR.A
sp|P50502|F10A1_HUMAN						
	4.8446	1470.781	1470.78	0.9	2696596	K.VAAIEALNDGELQK.A
	3.2179	1370.615	1370.614	0.4	1088542	K.LDYDEDASAMLK.E
sp|Q16181-2|SEPT7_HUMAN						
sp|Q16181|SEPT7_HUMAN						
	3.5211	1953.984	1953.982	1	1163007	K.NLEGYVGFANLPNQVYR.K
	2.6909	1566.712	1566.71	0.9	1325475	K.ADTLTPEECQQFK.K
sp|Q15365|PCBP1_HUMAN						
	3.254	1288.598	1288.595	2.5	14071398	R.INISEGNCPER.I
*	3.6933	1388.817	1388.815	1.2	10030720	R.IITLTGPTNAIFK.A
sp|Q9Y230-2|RUVB2_HUMAN						
sp|Q9Y230|RUVB2_HUMAN						
	3.2561	1158.637	1158.637	0.6	1320473	K.VQAGDVITIDK.A
	2.892	1684.862	1683.859	0.2	481853.5	R.TQGFLALFSGDTGEIK.S
sp|P21333-2|FLNA_HUMAN						
sp|P21333|FLNA_HUMAN						
	3.4067	1226.773	1226.772	1.3	5401510	R.LIALLEVLSQK.K
	4.8791	3230.504	3229.504	-1	1187575	R.ALGALVDSCAPGLCPDWDSWDASKPVTNAR.E
	3.477	1652.864	1652.86	2.4	2116431	K.VTAQGPGLEPSGNIANK.T
	3.773	1784.888	1784.885	1.8	406091.8	R.VQVQDNEGCPVEALVK.D
	2.6305	1225.573	1225.57	2.6	775937.9	R.EATTEFSVDAR.A
	4.6719	1763.858	1763.856	1.2	482988.5	R.VANPSGNLTETYVQDR.G
	3.8152	1909.92	1909.918	1.2	1250744	R.EGPYSISVLYGDEEVPR.S
	2.7174	1938.017	1938.018	-0.3	246099.9	K.DAGEGLLAVQITDPEGKPK.K
	3.9762	1500.755	1500.754	0.8	798870	K.DAGEGGLSLAIEGPSK.A
	3.4654	1533.77	1533.77	0.4	2887413	R.AEAGVPAEFSIWTR.E
sp|Q08380|LG3BP_HUMAN						
*	3.614	1592.792	1592.792	0.2	2998942	R.ELSEALGQIFDSQR.G
*	3.1584	1355.779	1355.778	0.7	3544428	R.SDLAVPSELALLK.A
*	2.5889	1206.554	1206.554	0.5	804214.9	K.AVDTWSWGER.A
sp|P22830-2|HEMH_HUMAN						
sp|P22830|HEMH_HUMAN						
	2.6843	1276.656	1276.653	2.4	1180727	R.AESLNGNPLFSK.A
	4.5674	1816.889	1816.887	1.4	2252525	K.QLTLSCPLCVNPVCR.E
sp|Q96RQ3|MCCA_HUMAN						
*	3.7373	1507.776	1507.775	0.5	1981919	K.LGVQTVAVYSEADR.N
*	3.8534	1580.72	1580.719	0.5	855891.6	R.SEQEFQEQLESAR.R
*	4.4645	2108.002	2108.001	0.6	580084.6	K.TFQVLGNLYSEGDCTYLK.C
sp|Q12874|SF3A3_HUMAN						
*	2.2686	2099.968	2099.967	0.5	423696.9	K.EELNAISGPNEFAEFYNR.L
*	3.1693	1310.686	1310.684	1.5	844923	K.SLESLDTSLFAK.N
sp|Q14677-2|EPN4_HUMAN						
sp|Q14677|EPN4_HUMAN						
sp|Q14677-3|EPN4_HUMAN						
	3.0385	1336.6	1336.602	-1.3	907715.8	K.ELVEFAQDDDR.L
	3.0677	1329.645	1329.643	1	2666083	K.YVGVSSDSVGGFR.Y
	3.5024	1149.6	1149.6	0.4	3055629	K.IGSTIDDTISK.F
sp|Q13813-2|SPTN1_HUMAN						
sp|Q13813|SPTN1_HUMAN						
sp|Q13813-3|SPTN1_HUMAN						
	2.6629	1205.606	1205.605	0.7	1599183	R.DVDETISWIK.E
	3.0731	1213.695	1213.694	1.1	2439550	R.AALLELWELR.R
	3.6589	1737.831	1737.829	1.3	576455.3	K.TATDEAYKDPSNLQGK.V
	2.7171	1119.585	1119.587	-1.3	645412.4	R.MNEVISLWK.K
	2.7903	1291.583	1291.58	2.3	2317509	R.DVEDEETWIR.E
	3.5567	1471.777	1471.775	1	2135180	R.ENLLEEQGSIALR.Q
	3.1533	1501.732	1501.728	2.8	2479765	R.EANELQQWINEK.E
	3.5893	1433.682	1433.679	2	2330633	R.DVDEIEAWISEK.L
	2.2818	1108.563	1108.563	0	1247387	K.LLVGSEDYGR.D
	2.5589	1204.679	1204.678	0.3	2800949	R.DLSSVQTLLTK.Q
	3.0007	1154.533	1154.532	0.2	1230215	K.SSEEIESAFR.A
	2.9986	1587.776	1587.769	4.2	2115029	R.ELPTAFDYVEFTR.S
sp|P29692-2|EF1D_HUMAN						
sp|P29692|EF1D_HUMAN						

sp|P29692-3|EF1D_HUMAN						
	3.8074	1358.728	1358.727	0.4	1751726	R.IASLEVENQSLR.G
	3.6988	1299.728	1299.727	1.2	1173364	R.GVVQELQQAISK.L
	2.9206	1346.663	1346.662	0.5	2010671	K.LQIQCVVEDDK.V
sp|O94925-3|GLSK_HUMAN						
sp|O94925|GLSK_HUMAN						
	2.6886	1480.619	1480.619	0.2	646193.6	K.DGPGETDAFGNSEGK.E
	3.8752	1922.91	1922.907	1.6	614230.7	K.FSPDLWGVSVCTVDGQR.H
sp|O95817|BAG3_HUMAN						
*	4.98	1915.911	1915.909	1.3	5618522	K.IDPQTGWPFFVDHNSR.T
*	4.0524	1413.724	1413.722	1.2	3090339	K.ELLALDSVDPEGR.A
sp|P30153|2AAA_HUMAN						
sp|P30154|2AAB_HUMAN						
sp|P30154-5|2AAB_HUMAN						
sp|P30154-4|2AAB_HUMAN						
sp|P30154-2|2AAB_HUMAN						
	3.4053	1242.743	1242.742	0.9	1599243	K.LSTIALALGVER.T
	4.6795	1942.051	1942.043	4.3	1024103	R.LNIISNLDCVNEVIGIR.Q
sp|Q16204|CCDC6_HUMAN						
*	3.0608	1684.809	1684.806	1.6	840597.9	R.AEQEEEFISNTLFK.K
*	2.3876	1066.542	1066.542	0.5	1952297	K.ETLAVNYEK.E
sp|P11498|PYC_HUMAN						
	3.2154	1408.629	1408.627	1.3	1231054	R.ADFAQACQDAGVR.F
*	3.4847	2226.193	2226.192	0.4	850666.2	K.ASPSPTDPVVPAVPIGPPPAGFR.D
*	3.4915	1363.677	1363.674	2.1	2256022	K.IAEEFEVELER.G
*	2.4745	958.5323	958.5316	0.7	2210373	K.ALAVSDLNR.A
sp|P23142-2|FBLN1_HUMAN						
sp|P23142|FBLN1_HUMAN						
sp|P23142-4|FBLN1_HUMAN						
sp|P23142-3|FBLN1_HUMAN						
	2.0291	1922.738	1922.738	0	209250	R.DSSCGTGYELTEDNSCK.D
	2.5892	1178.55	1178.548	2.3	2250484	K.TGYYFDGISR.M
sp|Q96AY3|FKB10_HUMAN						
*	2.8453	1509.793	1509.791	1.7	466799.9	R.ASPAGGPLEDVVIER.Y
*	3.1199	1197.732	1197.731	0.8	1482670	R.NTLVAIVVGVGR.L
sp|P26641-2|EF1G_HUMAN						
sp|P26641|EF1G_HUMAN						
	2.615	1118.683	1118.682	0.4	1100079	R.ILGLLDAYLK.T
	2.9099	1609.797	1609.794	1.6	625971	R.WFLTCINQPQFR.A
sp|Q96AE4-2|FUBP1_HUMAN						
sp|Q96AE4|FUBP1_HUMAN						
	3.3109	1973.892	1973.884	4.2	434023.9	K.IGGDAGTSLNSNDYGYGGQK.R
	2.162	988.4412	988.441	0.2	3193034	K.AWEEYYK.K
sp|Q92945|FUBP2_HUMAN						
*	3.7589	1533.803	1533.802	0.9	840818.8	K.AINQQTGAFVEISR.Q
	2.162	988.4412	988.441	0.2	3193034	K.AWEEYYK.K
sp|P14625|ENPL_HUMAN						
*	3.8798	1786.907	1785.898	3.3	381414.5	R.EEEAIQLDGLNASQIR.E
*	3.8187	1485.756	1485.754	0.7	495830.9	K.GVVDSDDLPLNVSR.E
sp|P19338|NUCL_HUMAN						
*	3.9993	1594.746	1594.742	2.1	2059586	K.GYAFIEFASFEDAK.E
*	2.9207	1322.636	1322.632	2.7	1822921	K.GLSEDTTEETLK.E
sp|P78344-2|IF4G2_HUMAN						
sp|P78344|IF4G2_HUMAN						
	3.2612	2011.935	2011.936	-0.4	337773.1	R.LAEDAPNFDGPAAEGQPGQK.Q
	2.961	1408.713	1408.711	1.6	1404032	R.EWLTELFQQSK.V
sp|Q13492-2|PICAL_HUMAN						
sp|Q13492|PICAL_HUMAN						
sp|Q13492-5|PICAL_HUMAN						
sp|Q13492-4|PICAL_HUMAN						
sp|Q13492-3|PICAL_HUMAN						
	3.0551	1267.669	1267.668	0.8	716446.2	R.TTNSSWVVVFK.S
	3.2714	1425.738	1425.737	0.7	831290.6	R.NTLFNLSNFLDK.S
sp|Q14157-1|UBP2L_HUMAN						
sp|Q14157|UBP2L_HUMAN						
sp|Q14157-5|UBP2L_HUMAN						

sp|Q14157-4|UBP2L_HUMAN						
sp|Q14157-3|UBP2L_HUMAN						
	4.3387	2240.938	2240.947	-4	419037.5	R.TATEEWGTEDWNEDLSETK.I
	2.7432	1524.719	1524.718	0.6	339598.7	R.DGSLASNPYSGDLTK.F
sp|Q14974|IMB1_HUMAN						
*	4.0234	1658.913	1658.911	1.2	2670808	R.AAVENLPTFLVELSR.V
	1.4962	1765.626	1765.624	0.8	238628.7	K.QDENDDDDDWNPCK.A
sp|P35606-2|COPB2_HUMAN						
sp|P35606|COPB2_HUMAN						
	3.7277	1735.887	1735.886	0.6	673576.5	R.GSNNVALGYDEGSIIVK.L
	2.9187	1235.632	1235.631	0.8	2096065	K.EAFVVEEWVK.E
sp|P48681|NEST_HUMAN						
*	3.616	1372.672	1372.67	1.1	1360571	R.DNLAEELEGVAGR.C
*	3.043	1554.826	1554.824	1.6	633733.1	R.AQDAPLSLLQTQGGR.K
*	2.5724	1183.67	1183.668	1.9	1071641	K.DVEVVRPLEK.E
*	2.7107	1272.671	1272.67	1.4	841071.8	R.SLGAWNLENLR.S
sp|Q09666|AHNK_HUMAN						
*	2.637	1578.689	1578.688	0.9	665332.4	K.SEDGVEGDLGETQSR.T
*	3.0344	1267.654	1267.653	0.7	1790265	K.AEGPEVDVNLPK.A
*	2.8375	1239.622	1239.622	0	946141.1	K.GEGPDVDVNLPK.A
sp|Q9UPQ0-10|LIMC1_HUMAN						
sp|Q9UPQ0|LIMC1_HUMAN						
sp|Q9UPQ0-9|LIMC1_HUMAN						
sp|Q9UPQ0-8|LIMC1_HUMAN						
sp|Q9UPQ0-6|LIMC1_HUMAN						
sp|Q9UPQ0-5|LIMC1_HUMAN						
sp|Q9UPQ0-4|LIMC1_HUMAN						
sp|Q9UPQ0-3|LIMC1_HUMAN						
sp|Q9UPQ0-2|LIMC1_HUMAN						
	3.6166	1496.82	1496.818	1.2	731425.6	R.QEQLQLINNQLR.E
	2.8567	1274.635	1274.634	1.4	1429854	K.GQLGDAVSGTDVR.I
sp|O75369-2|FLNB_HUMAN						
sp|O75369|FLNB_HUMAN						
sp|O75369-9|FLNB_HUMAN						
sp|O75369-8|FLNB_HUMAN						
	3.4067	1226.773	1226.772	1.3	5401510	R.LIALLEVLSQK.R
	3.8059	1656.683	1656.681	1.5	630755.9	K.IEYNDQNDGSCDVK.Y
	3.205	1644.864	1644.863	1	312089.9	R.APSVATVGSICDLNLK.I
	3.9342	1442.751	1442.749	1.5	1275015	R.EAGAGGLSIAVEGPSK.A
sp|P35579|MYH9_HUMAN						
*	3.662	1284.717	1284.716	0.6	1016685	K.VEAQLQELQVK.F
	3.0882	1348.623	1348.623	-0.1	899713.4	R.DELADEIANSSGK.G
	3.558	1530.762	1530.765	-1.5	994480.8	K.IAQLEEQLDNETK.E
sp|Q14315-2|FLNC_HUMAN						
sp|Q14315|FLNC_HUMAN						
	3.4067	1226.773	1226.772	1.3	5401510	R.LIALLEVLSQK.R
	2.5077	1584.824	1584.823	0.5	538944.2	R.GAGTGGLGLAIEGPSEAK.M
	3.9342	1442.751	1442.749	1.5	1275015	R.EAGAGGLSIAVEGPSK.A
sp|P46821|MAP1B_HUMAN						
*	2.9771	1768.672	1768.671	0.8	273376.5	R.SPDEEDYDYESYEK.T
*	3.1218	1490.667	1490.665	1.5	704601.8	K.SPSDSGYSYETIGK.T
*	2.4615	1658.745	1658.743	1.1	396778.1	K.TPEDGDYSYEIIEK.T

Supplymentary Table 4. Raw data D129A mutant R-catcher

Unique	XCorr	M+H+	CalcM+H+	PPM	TotalIntensity	Sequence
sp|Q01105-2|SET_HUMAN						
*	4.8213	1774.777	1773.7886	-8.7	2.00E+07	K.KELNSNHDGADETSEK.E
	6.048	2195.022	2195.021	0.3	8059251.5	K.EQQEAIEHIDEVQNEIDR.L
	7.9053	3449.664	3449.6665	-0.8	2.66E+07	K.EQQEAIEHIDEVQNEIDRLNEQASEEILK.V
	5.652	3449.669	3449.6665	0.8	3.11E+07	K.EQQEAIEHIDEVQNEIDRLNEQASEEILK.V
	3.5511	1208.605	1208.6045	0.7	5.29E+07	R.VEVTEFEDIK.S
	5.2062	1840.809	1840.8064	1.3	2.49E+07	R.IDFYFDENPYFENK.V
	4.0354	1840.807	1840.8064	0.1	3250567.5	R.IDFYFDENPYFENK.V
	4.6554	1446.652	1446.6495	1.9	3.55E+07	K.EFHLNESGDPSSK.S
	9.5654	3377.536	3377.5344	0.4	9917632	K.RQHEEPESFFTWFTDHSDAGADELGEVIK.D
	9.1835	3377.537	3377.5344	0.7	1.43E+07	K.RQHEEPESFFTWFTDHSDAGADELGEVIK.D
	7.4784	3377.539	3377.5344	1.2	7822219.5	K.RQHEEPESFFTWFTDHSDAGADELGEVIK.D
sp|Q01105|SET_HUMAN						
*	5.422	2100.146	2100.1448	0.6	837556.1	K.KPRPPPALGPEETSASAGLPK.K
	6.048	2195.022	2195.021	0.3	8059251.5	K.EQQEAIEHIDEVQNEIDR.L
	7.9053	3449.664	3449.6665	-0.8	2.66E+07	K.EQQEAIEHIDEVQNEIDRLNEQASEEILK.V
	5.652	3449.669	3449.6665	0.8	3.11E+07	K.EQQEAIEHIDEVQNEIDRLNEQASEEILK.V
	3.5511	1208.605	1208.6045	0.7	5.29E+07	R.VEVTEFEDIK.S
	5.2062	1840.809	1840.8064	1.3	2.49E+07	R.IDFYFDENPYFENK.V
	4.0354	1840.807	1840.8064	0.1	3250567.5	R.IDFYFDENPYFENK.V
	4.6554	1446.652	1446.6495	1.9	3.55E+07	K.EFHLNESGDPSSK.S
	9.5654	3377.536	3377.5344	0.4	9917632	K.RQHEEPESFFTWFTDHSDAGADELGEVIK.D
	9.1835	3377.537	3377.5344	0.7	1.43E+07	K.RQHEEPESFFTWFTDHSDAGADELGEVIK.D
	7.4784	3377.539	3377.5344	1.2	7822219.5	K.RQHEEPESFFTWFTDHSDAGADELGEVIK.D
sp|P47929|LEG7_HUMAN						
*	3.717	1238.628	1238.6263	1	1117492.2	R.LDTSEVVFNSK.E
*	4.5422	1735.893	1735.8901	1.5	4064973.2	R.GQPFEVLIIASDDGFK.A
*	3.52	1485.792	1485.7908	1.1	1283828	R.LVEVGGDVQLDSVR.I
sp|P11021|BIP_HUMAN						
	3.249	1566.782	1566.7798	1.2	3149557	R.ITPSYVAFTPEGER.L
	3.3143	1430.692	1430.691	0.5	2606851.5	R.TWNDPSVQQDIK.F
	3.8365	1536.8	1536.7977	1.4	3177451	K.TFAPEEISAMVLTK.M
	4.0358	1887.971	1887.9712	-0.3	1140999.9	K.VTHAVVTVPAYFNDAQR.Q
	5.4754	1659.898	1659.8951	1.4	6725170.5	R.IINEPTAAAIAYGLDK.R
	4.0929	2164.991	2164.992	-0.3	1393771	R.IEIESFYEGEDFSETLTR.A
	4.533	1836.936	1836.9337	1.1	1227567.1	K.SQIFSTASDNQPTVTIK.V
	2.7797	1191.637	1191.6368	0.1	1.49E+07	K.VYEGERPLTK.D
	4.5401	1974.909	1974.908	0.4	561031.5	K.IEWLESHQDADIEDFK.A
	5.1316	1974.913	1974.908	2.4	1947756	K.IEWLESHQDADIEDFK.A
	4.0694	1525.883	1525.8835	-0.3	844459	K.KELEEIVQPIISK.L
	3.3528	1397.791	1397.7886	1.4	2870262	K.ELEEIVQPIISK.L
	5.3155	1819.845	1818.8392	1.4	929324.4	K.LYGSAGPPPTGEEDTAEK.D
	3.7321	2175.994	2175.9927	0.4	524396.5	K.LYGSAGPPPTGEEDTAEKDEL.-
sp|Q96I24|FUBP3_HUMAN						
*	6.0922	2713.388	2713.383	1.6	4852567.5	K.IDSIPHLNNSTPLVDPSVYGYGVQK.R
	5.0038	2997.52	2997.5198	0.2	1666804.4	K.IQIASESSGIPERPCVLTGTPESIEQAK.R
	6.0866	2567.277	2567.2737	1.1	4009025	R.NGPGFHNDIDSNSTIQEILIPASK.V
*	5.3715	2247.092	2247.0928	-0.2	2991130	R.GDWSVGAPGGVQEITYTVPADK.C
sp|P11498|PYC_HUMAN						
	4.2857	1409.627	1408.6274	-2.9	796560.4	R.ADFAQACQDAGVR.F
	4.7144	1796.882	1796.8813	0.5	1971692.4	K.QVGYENAGTVEFLVDR.H
	7.7323	2345.215	2345.2136	0.5	5522235.5	R.LDNASAFQGAVISPHYDSLLVK.V
*	3.9657	2226.19	2226.1917	-0.5	2256255	K.ASPSPTDPVVPAVPIGPPPAGFR.D
*	4.9505	1828.889	1828.8865	1.2	1377006.4	R.GANAVGYTNYPDNVVFK.F
*	4.1485	1747.932	1747.916	8.9	2783934.2	K.IVGDLAQFMVQNGLSR.A
*	4.8099	1547.734	1547.7336	0.5	2067665.8	R.AEAEAQAEELSFPR.S
*	6.0833	2432.246	2432.2397	2.4	3611383	R.SVVEFLQGYIGVPHGGFPEPFR.S
*	3.4776	1548.866	1548.8632	1.5	1268602.9	R.PGASLPPLDLQALEK.E
*	3.5578	1363.675	1363.674	1	1685424.2	K.IAEEFEVELER.G
sp|P0DMV8-2|HS71A_HUMAN						

sp|P0DMV9|HS71B_HUMAN						
sp|P0DMV8|HS71A_HUMAN						
	2.8807	1487.703	1487.7013	1.4	985412.4	R.TTPSYVAFTDTER.L
	5.1531	1658.852	1658.8496	1.3	2106557.8	K.NQVALNPQNTVFDAK.R
	2.5593	1222.578	1222.5773	0.3	690685	K.FGDPVVQSDMK.H
	3.4656	1197.696	1197.695	0.7	4159087.5	K.DAGVIAGLNVLR.I
	4.5608	1687.906	1687.9014	2.7	3988701.8	R.IINEPTAAAIAYGLDR.T
	2.4876	1204.533	1204.5328	0.4	1028933.8	K.GGSGSGPTIEEVD.-
sp|P67809|YBOX1_HUMAN						
	3.2529	1795.825	1795.8232	0.7	310981.6	R.SVGDGETVEFDVVEGEK.G
*	3.5751	1695.869	1695.8661	1.7	375020.8	K.GAEAANVTGPGGVPVQGSK.Y
sp|P68363-2|TBA1B_HUMAN						
sp|Q9BQE3|TBA1C_HUMAN						
sp|P68363|TBA1B_HUMAN						
	5.3722	2007.895	2007.8931	1.1	1080804.9	K.TIGGGDDSFNTFFSETGAGK.H
	3.466	1487.883	1487.8792	2.9	2462719.5	R.LISQIVSSITASLR.F
sp|P60709|ACTB_HUMAN						
sp|P63261|ACTG_HUMAN						
	2.478	1198.523	1198.5222	0.4	2886709.8	K.DSYVGDEAQSK.R
	2.3288	1132.528	1132.527	0.8	1215194.8	R.GYSFTTTAER.E
	4.5656	1790.895	1790.8918	1.5	2966935.5	K.SYELPDGQVITIGNER.F
sp|P04406-2|G3P_HUMAN						
sp|P04406|G3P_HUMAN						
	3.055	1530.796	1530.7944	1	262204.1	R.VPTANVSVVDLTCR.L
	3.338	1763.805	1763.8024	1.5	925296.7	K.LISWYDNEFGYSNR.V
sp|Q7Z5L9-2|I2BP2_HUMAN						
sp|Q7Z5L9|I2BP2_HUMAN						
	5.8062	1930.937	1930.9352	1.1	835691.1	R.GPADSLSTAAGAAELSAEGAGK.S
	2.4937	1596.7	1596.6958	2.4	279173.1	K.QQGASGEVYCPSGEK.C
sp|Q9Y2S7|PDIP2_HUMAN						
*	3.7562	1316.748	1316.746	1.6	2121252	K.VLETVGVFEVPK.Q
*	3.7624	1400.739	1400.7379	0.8	711468.1	R.LENLDSDVVQLR.E
sp|P34931|HS71L_HUMAN						
	2.8807	1487.703	1487.7013	1.4	985412.4	R.TTPSYVAFTDTER.L
	3.4656	1197.696	1197.695	0.7	4159087.5	K.DAGVIAGLNVLR.I
	5.4754	1659.898	1659.8951	1.4	6725170.5	R.IINEPTAAAIAYGLDK.G
sp|Q96RQ3|MCCA_HUMAN						
*	3.2535	1507.776	1507.775	0.5	411349.9	K.LGVQTVAVYSEADR.N
*	3.8146	1594.898	1594.8951	1.5	2595425.2	K.QEGIIFIGPPPSAIR.D
*	3.3612	1415.796	1415.7926	2.1	528743.6	K.ESLCQAALGLILK.E
sp|O43813|LANC1_HUMAN						
*	2.0355	1299.602	1299.6005	1	3502200.5	R.AFPNPYADYNK.S
*	4.1329	1413.816	1413.8141	1.6	7136045.5	R.IGYIYALLFVNK.N
sp|Q13501-2|SQSTM_HUMAN						
sp|Q13501|SQSTM_HUMAN						
	4.945	2239.909	2239.9126	-1.7	848397.9	R.YKCSVCPDYDLCSVCEGK.G
	5.8469	1948.756	1948.7544	0.8	5.31E+08	K.CSVCPDYDLCSVCEGK.G
	4.5027	1948.755	1948.7544	0.3	9399459	K.CSVCPDYDLCSVCEGK.G
sp|P52272-2|HNRPM_HUMAN						
sp|P52272|HNRPM_HUMAN						
	3.536	1426.759	1426.7577	0.9	379187.5	R.LGSTVFVANLDYK.V
	4.0255	1284.63	1284.6292	0.9	703941.8	K.QGGGGGGGSVPGIER.M
sp|P15924|DESP_HUMAN						
	3.6619	1271.707	1271.7067	0.5	1686761.1	R.QLQNIIQATSR.E
*	4.05	1685.945	1685.9432	1.1	317603.4	K.ITNLTQQLEQASIVK.K
	3.5636	1414.706	1414.7074	-1	917752.7	K.QQIQNDLNQWK.T
	2.3776	1713.73	1713.7272	1.9	193769.1	R.ETQTECEWTVDTSK.L
	3.6463	1538.88	1538.8788	0.9	339829.7	R.LLEAQIATGGIIDPK.E
	3.7474	1387.716	1387.7137	1.8	740305.7	K.DEETGLCLLPLK.E
sp|Q02413|DSG1_HUMAN						
*	3.3868	1635.835	1635.8336	0.5	687520.2	K.YQGTILSIDDNLQR.T
	3.1835	1089.554	1089.5535	0.8	1337083.2	R.DGSNVIVTER.V
sp|Q13085-2|ACACA_HUMAN						

sp|Q13085|ACACA_HUMAN						
sp|Q13085-4|ACACA_HUMAN						
sp|Q13085-3|ACACA_HUMAN						
	3.1252	1439.718	1439.7166	1.1	909802.8	R.DFTVASPAEFVTR.F
	3.0076	1571.698	1571.6973	0.2	401912.9	K.EASFEYLQNEGER.L
	3.0301	1591.85	1591.8479	1.5	469190.5	R.IGSFGPQEDLLFLR.A

Supplymentary Table 5. Raw data WT R-catcher RA competition

Unique	XCorr	M+H+	CalcM+H+	PPM	TotalIntensity	Sequence
sp|Q01105-2|SET_HUMAN						
sp|Q01105|SET_HUMAN						
sp|Q01105-4|SET_HUMAN						
sp|Q01105-3|SET_HUMAN						
	5.745	2195.023	2195.021	0.7	4506457.5	K.EQQEAIEHIDEVQNEIDR.L
	7.2267	3449.666	3449.667	-0.1	1.58E+07	K.EQQEAIEHIDEVQNEIDRLNEQASEEILK.V
	3.3932	1208.604	1208.605	-0.1	4107219.8	R.VEVTEFEDIK.S
	5.0908	1840.808	1840.806	1.1	3.10E+07	R.IDFYFDENPYFENK.V
	4.0839	1840.806	1840.806	-0.1	5285331.5	R.IDFYFDENPYFENK.V
	3.4981	1446.651	1446.65	1	1866407.8	K.EFHLNESGDPSSK.S
	9.6622	3377.544	3377.534	3	8419458	K.RQHEEPESFFTWFTDHSDAGADELGEVIK.D
	7.1403	3377.542	3377.534	2.2	7516068.5	K.RQHEEPESFFTWFTDHSDAGADELGEVIK.D
sp|P62987|RL40_HUMAN						
sp|P62979|RS27A_HUMAN						
sp|P0CG48|UBC_HUMAN						
sp|P0CG47|UBB_HUMAN						
	4.7542	1787.928	1787.927	0.5	1036572.3	K.TITLEVEPSDTIENVK.A
	3.0434	1067.621	1067.621	0	776393	K.ESTLHLVLR.L
sp|P0DMV8|HS71A_HUMAN						
sp|P0DMV9|HS71B_HUMAN						
	3.0058	1487.704	1487.701	1.6	5554505	R.TTPSYVAFTDTER.L
	4.2318	1658.852	1658.85	1.2	2015057.4	K.NQVALNPQNTVFDAK.R
	2.8922	1222.578	1222.577	0.3	962305.6	K.FGDPVVQSDMK.H
	5.0118	1680.85	1680.849	0.2	3877272.8	K.HWPFQVINDGDKPK.V
	3.0854	1614.811	1614.808	1.8	3647808	K.AFYPEEISSMVLTK.M
	3.474	1197.696	1197.695	1	2.41E+07	K.DAGVIAGLNVLR.I
	4.8066	1687.904	1687.901	1.7	2.36E+07	R.IINEPTAAAIAYGLDR.T
	3.3794	1675.733	1675.731	1.3	6052030	K.ATAGDTHLGGEDFDNR.L
	3.0934	1542.742	1542.737	3.2	1718935.9	R.ARFEELCSDLFR.S
	4.9483	2786.368	2786.363	1.7	2868958	K.QTQIFTTYSDNQPGVLIQVYEGER.A
	5.1962	2786.376	2786.363	4.5	1.24E+07	K.QTQIFTTYSDNQPGVLIQVYEGER.A
	2.8344	1287.605	1287.604	0.6	946160.8	K.NALESYAFNMK.S
	5.1033	1876.912	1876.911	0.6	2573023.5	K.CQEVISWLDANTLAEK.D
	2.7765	1204.534	1204.533	0.9	4549501.5	K.GGSGSGPTIEEVD.-
sp|P10599-2|THIO_HUMAN						
sp|P10599|THIO_HUMAN						
	5.1472	1738.83	1738.829	0.6	4089833.2	K.LVVVDFSATWCGPCK.M
	2.7864	1001.552	1001.551	0.3	3658157.5	K.LEATINELV.-
sp|P63208|SKP1_HUMAN						
	5.7034	1878.934	1878.933	0.6	1360448.8	K.LQSSDGEIFEVDVEIAK.Q
	4.7489	1761.882	1761.881	1	1119919.1	K.RTDDIPVWDQEFLK.V
*	3.5411	1466.64	1466.639	0.7	5180052.5	K.NDFTEEEEAQVR.K
sp|P11021|BIP_HUMAN						
	3.7053	1566.782	1566.78	1.2	9964064	R.ITPSYVAFTPEGER.L
	3.5875	1430.692	1430.691	0.7	6891278.5	R.TWNDPSVQQDIK.F
	3.2976	1536.798	1536.798	0.4	6896303	K.TFAPEEISAMVLTK.M
	3.2846	1887.976	1887.971	2.3	436189.9	K.VTHAVVTVPAYFNDAQR.Q
	4.1617	1887.972	1887.971	0.3	1818499.6	K.VTHAVVTVPAYFNDAQR.Q
	3.0731	1217.63	1217.631	-0.8	1211708.1	K.DAGTIAGLNVMR.I
	5.3694	1659.898	1659.895	1.5	6015632.5	R.IINEPTAAAIAYGLDK.R
	5.2838	2164.989	2164.992	-1.2	1.36E+07	R.IEIESFYEGEDFSETLTR.A
	5.098	1836.936	1836.934	1.3	2417613	K.SQIFSTASDNQPTVTIK.V
	4.73	1974.907	1974.908	-0.6	1823742.8	K.IEWLESHQDADIEDFK.A
	5.3502	1974.913	1974.908	2.4	3804305.2	K.IEWLESHQDADIEDFK.A
	5.6851	1525.884	1525.884	0.5	722141.7	K.KELEEIVQPIISK.L
	3.5244	1397.79	1397.789	1	2748283.2	K.ELEEIVQPIISK.L
	4.2955	1818.842	1818.839	1.3	4529167.5	K.LYGSAGPPPTGEEDTAEK.D
	5.6069	2175.993	2175.993	0.2	2791214.5	K.LYGSAGPPPTGEEDTAEKDEL.-
	5.0712	2175.995	2175.993	1	825031.8	K.LYGSAGPPPTGEEDTAEKDEL.-
sp|P67775|PP2AA_HUMAN						
	4.4453	1704.792	1704.79	1.4	2120772.5	K.ELDQWIEQLNECK.Q
	4.8503	1791.838	1791.837	0.5	2191823	R.QITQVYGFYDECLR.K
	4.6166	2655.282	2655.28	0.6	2466528.2	R.GAGYTFGQDISETFNHANGLTLVSR.A

	4.4396	1703.826	1703.821	3.1	4428438	R.NVVTIFSAPNYCYR.C
	3.2931	1340.667	1340.663	2.7	3567943.8	K.YSFLQFDPAPR.R
sp|Q9Y587-4|AP4S1_HUMAN						
*	1.585	3563.713	3561.682	6.7	361738.3	M.HSGPYQTRSCSVTQAGVQRCDHGSLHPGSPGLK.-
*	1.8974	1619.799	1617.791	0.5	1260257.1	Q.RCDHGSLHPGSPGLK.-
sp|P12273|PIP_HUMAN						
*	5.8966	2069.125	2069.124	0.7	2079071.2	K.SVRPNDEVTAVLAVQTELK.E
*	3.4475	1283.77	1283.768	1.3	5014411.5	R.TVQIAAVVDVIR.E
sp|P81605-2|DCD_HUMAN						
sp|P81605|DCD_HUMAN						
	2.8284	1128.53	1128.528	1.8	3338891.5	K.ENAGEDPGLAR.Q
	3.9849	1217.674	1217.674	0.7	5414135.5	R.SSLLEKGLDGAK.K
sp|Q06830|PRDX1_HUMAN						
*	4.8218	1622.858	1622.857	0.3	1768792.8	K.QGGLGPMNIPLVSDPK.R
*	2.6645	1107.605	1107.604	0.3	2327554.5	R.TIAQDYGVLK.A
	3.7438	1140.525	1140.526	-0.1	1451902.8	K.HGEVCPAGWK.P
sp|P63098|CANB1_HUMAN						
*	2.8693	1369.701	1369.7	1	944235	K.EFIEGVSQFSVK.G
*	4.2282	1920.955	1920.952	1.4	1483298.2	R.ISFEEFCAVVGGLDIHK.K
sp|P14618-2|KPYM_HUMAN						
sp|P14618|KPYM_HUMAN						
	3.0796	1197.648	1197.647	0.6	830187.9	R.LDIDSPPITAR.N
	3.1248	1359.706	1359.705	0.5	595435.5	R.NTGIICTIGPASR.S
	5.526	2465.298	2465.292	2.3	6081171.5	R.TATESFASDPILYRPVAVALDTK.G
	3.9986	1468.681	1468.678	2	1402633	K.CDENILWLDYK.N
	4.0258	1779.88	1779.876	2.5	653895.8	K.GADFLVTEVENGGSLGSK.K
	4.9559	1642.773	1642.771	1.3	1064496.2	K.DPVQEAWAEDVDLR.V
sp|P34931|HS71L_HUMAN						
	3.0058	1487.704	1487.701	1.6	5554505	R.TTPSYVAFTDTER.L
	3.0854	1614.811	1614.808	1.8	3647808	K.AFYPEEISSMVLTK.L
	3.474	1197.696	1197.695	1	2.41E+07	K.DAGVIAGLNVLR.I
	5.3694	1659.898	1659.895	1.5	6015632.5	R.IINEPTAAAIAYGLDK.G
	3.3794	1675.733	1675.731	1.3	6052030	K.ATAGDTHLGGEDFDNR.L
	4.9483	2786.368	2786.363	1.7	2868958	K.QTQIFTTYSDNQPGVLIQVYEGER.A
	5.1962	2786.376	2786.363	4.5	1.24E+07	K.QTQIFTTYSDNQPGVLIQVYEGER.A
	2.8344	1287.605	1287.604	0.6	946160.8	K.NALESYAFNMK.S
sp|P17066|HSP76_HUMAN						
	3.0058	1487.704	1487.701	1.6	5554505	R.TTPSYVAFTDTER.L
	3.3794	1305.671	1305.669	1.7	1884094.8	K.ETAEAYLGQPVK.H
	4.8066	1687.904	1687.901	1.7	2.36E+07	R.IINEPTAAAIAYGLDR.R
	3.3794	1675.733	1675.731	1.3	6052030	K.ATAGDTHLGGEDFDNR.L
	3.0934	1542.742	1542.737	3.2	1718935.9	R.ARFEELCSDLFR.S
*	3.7571	1721.808	1720.807	-1.7	1249542.2	R.LYGGPGVPGGSSCGTQAR.Q
*	3.2412	1456.682	1456.68	1.5	863066.4	R.QGDPSTGPIIEEVD.-
sp|P68363-2|TBA1B_HUMAN						
sp|Q9BQE3|TBA1C_HUMAN						
sp|P68363|TBA1B_HUMAN						
	6.6652	2007.894	2007.893	0.5	3388195	K.TIGGGDDSFNTFFSETGAGK.H
	4.5723	1487.882	1487.879	1.7	6380180.5	R.LISQIVSSITASLR.F
	3.3817	1824.987	1824.985	0.9	1293168.4	K.VGINYQPPTVVPGGDLAK.V
sp|O43813|LANC1_HUMAN						
*	4.1935	1413.817	1413.814	1.7	1.47E+07	R.IGYIYALLFVNK.N
*	4.811	1886.022	1886.02	0.6	2482158.8	K.LHSLVKPSVDYVCQLK.F
*	3.0512	1208.599	1208.598	0.8	396429.1	K.PSVDYVCQLK.F
*	2.5855	1593.713	1593.711	1.2	429289.1	K.FPSGNYPPCIGDNR.D
*	4.8293	1813.742	1813.742	0	1263299.8	K.FAEWCLEYGEHGCR.T
sp|O76003|GLRX3_HUMAN						
*	3.972	2069.959	2069.956	1.3	1236223.1	K.HNIQFSSFDIFSDEEVR.Q
*	3.9768	1633.763	1633.763	0.1	441789.5	K.ELEASEELDTICPK.A
*	4.5536	1729.861	1729.858	1.3	2274249.2	K.AYSNWPTYPQLYVK.G
sp|P68366-2|TBA4A_HUMAN						
sp|P68366|TBA4A_HUMAN						
	2.377	2067.894	2067.897	-1.3	290936.4	K.TIGGGDDSFTTFFCETGAGK.H
	4.5723	1487.882	1487.879	1.7	6380180.5	R.LISQIVSSITASLR.F
	3.3817	1824.987	1824.985	0.9	1293168.4	K.VGINYQPPTVVPGGDLAK.V
sp|P31151|S10A7_HUMAN						

*	4.7562	1384.712	1384.711	1.1	8643172	K.KGTNYLADVFEK.K
*	3.5542	1256.617	1256.616	1.1	3146530.8	K.GTNYLADVFEK.K
sp|P07355-2|ANXA2_HUMAN						
sp|P07355|ANXA2_HUMAN						
	5.0259	1542.851	1542.849	1.8	3296524.5	K.GVDEVTIVNILTNR.S
	4.7115	1650.98	1650.979	0.7	4751455	K.SALSGHLETVILGLLK.T
	3.2188	1222.598	1222.595	2.2	1744544.5	K.TPAQYDASELK.A
sp|P30153|2AAA_HUMAN						
	3.4584	1242.742	1242.742	0.5	3418643.2	K.LSTIALALGVER.T
*	3.305	1109.537	1109.538	-0.1	2069270.2	R.LAGGDWFTSR.T
*	2.8912	1400.557	1400.557	0.3	1805566.2	K.EFCENLSADCR.E
	6.3404	1942.043	1942.043	0.3	7678940.5	R.LNIISNLDCVNEVIGIR.Q
*	3.1728	1160.58	1160.579	0.4	4725006	K.LTQDQDVDVK.Y
sp|Q9UKD1|GMEB2_HUMAN						
*	1.8546	5400.808	5400.774	6.2	6370524	Q.LLTLPGLGPTLQNVAQASPGSSTIVTVPAGAAP.-
*	1.3394	2490.168	2489.173	-3.3	239838.8	A.APGPEEHTATIEVAAMAEDHERK.-
*	2.0357	2490.159	2489.173	-6.9	4298353.5	A.APGPEEHTATIEVAAMAEDHERK.-
*	1.7747	2166.994	2167.008	-6.5	989152.9	P.EEHTATIEVAAMAEDHERK.-
sp|Q9NUS5|AP5S1_HUMAN						
*	2.4743	2382.167	2382.188	-8.6	2429182.8	L.FLNDQFVQGLEKEFSAAWPR.-
*	4.1195	2382.171	2382.188	-7	2460194.8	L.FLNDQFVQGLEKEFSAAWPR.-
sp|P04406-2|G3P_HUMAN						
sp|P04406|G3P_HUMAN						
	3.0937	1613.899	1613.901	-1.4	780570.7	K.LVINGNPITIFQER.D
	2.9898	1763.803	1763.802	0.2	342704.3	K.LISWYDNEFGYSNR.V
sp|P00505-2|AATM_HUMAN						
sp|P00505|AATM_HUMAN						
	2.153	3403.804	3403.779	7.5	4395465.5	F.SIYMTKDGRISVAGVTSSNVGYLAHAIHQVTK.-
	1.0344	2809.5	2808.512	-5.5	180370.4	T.KDGRISVAGVTSSNVGYLAHAIHQVTK.-
sp|P08670|VIME_HUMAN						
*	3.311	1088.535	1088.533	1.7	2145736.5	R.QDVDNASLAR.L
*	4.1401	1405.759	1405.757	1.4	5514965	K.VESLQEEIAFLK.K
*	3.2559	1570.9	1570.895	3.3	2521843.2	R.ISLPLPNFSSLNLR.E
sp|Q9Y2S7|PDIP2_HUMAN						
*	3.7882	1316.748	1316.746	1.5	3685994.8	K.VLETVGVFEVPK.Q
*	3.8069	1830.913	1830.917	-2.6	779023.3	K.YETGQLFLHSIFGYR.G
sp|O43435-3|TBX1_HUMAN						
*	1.8911	3059.376	3058.378	-1.8	610114	A.AAAAAAAAAAAAAANMYSSAGAAPPGSYDYCPR.-
*	1.9211	2705.215	2703.193	5.8	6495242	A.AAAAAAAAANMYSSAGAAPPGSYDYCPR.-
sp|Q14764|MVP_HUMAN						
*	3.1037	5902.004	5901.025	-4.2	2.29E+07	D.GSTPINLFNTAFGLLGMGPEGQPLGRRVASGPSPGE.-
*	2.4879	5843.974	5844.004	-5.2	8.86E+07	G.STPINLFNTAFGLLGMGPEGQPLGRRVASGPSPGEG.-
*	2.7425	5844.981	5844.004	-4.6	3.01E+07	G.STPINLFNTAFGLLGMGPEGQPLGRRVASGPSPGEG-
sp|P11142|HSP7C_HUMAN						
	3.0058	1487.704	1487.701	1.6	5554505	R.TTPSYVAFTDTER.L
	5.3694	1659.898	1659.895	1.5	6015632.5	R.IINEPTAAAIAYGLDK.K
*	3.2931	1304.632	1304.63	1.2	1166012.8	K.CNEIINWLDK.N
sp|Q13501-2|SQSTM_HUMAN						
sp|Q13501|SQSTM_HUMAN						
	2.0732	2239.913	2239.913	0.1	208861.4	R.YKCSVCPDYDLCSVCEGK.G
	6.4131	2239.915	2239.913	1	4814667	R.YKCSVCPDYDLCSVCEGK.G
	5.6541	1948.755	1948.754	0.2	4830830	K.CSVCPDYDLCSVCEGK.G
	5.1898	1948.755	1948.754	0.5	7.88E+07	K.CSVCPDYDLCSVCEGK.G
sp|Q9NP62|GCM1_HUMAN						
*	2.4449	2433.147	2432.152	-3.3	5504187.5	M.TYLGLDHCNNDMLLNLCPLR.-
*	1.6095	2169.038	2168.041	-3.1	1341702.2	Y.LGLDHCNNDMLLNLCPLR.-
sp|P14543-2|NID1_HUMAN						
sp|P14543|NID1_HUMAN						
	2.5607	2749.292	2748.286	0.9	2171461.8	C.LATPGSRTCRCPDNTLGVDCIEQK.-
	1.2545	1965.872	1964.862	3.3	1014091	T.CRCPDNTLGVDCIEQK.-
sp|P11498|PYC_HUMAN						
*	3.9916	1547.735	1547.734	0.9	648377.2	R.AEAEAQAEELSFPR.S
*	3.7516	1363.677	1363.674	1.9	1296240.9	K.IAEEFEVELER.G
sp|Q6N022|TEN4_HUMAN						
*	1.2052	2767.267	2766.283	-7.2	355841.8	E.QYPELSDSANNIHFMRQSEMGRR.-
*	1.1513	1848.884	1846.891	-7.4	318629.9	S.ANNIHFMRQSEMGRR.-

Supplymentary Table 6. Final working list

		WT Rcatcher			WT Rcather with AR competition			D129A Mutant Rcatcher			WT Rcatcher with RA competition			
**Uniprot ID**	**Unique uniprot ID/Gene name**	**Spectrum count**	**Sequence coverage (%)**	**Sum of peptide intensity**	**Spectrum count**	**Sequence coverage (%)**	**Sum of peptide intensity**	**Spectrum count**	**Sequence coverage (%)**	**Sum of peptide intensity**	**Spectrum count**	**Sequence coverage (%)**	**Sum of peptide intensity**	**No. of unique intensity**
P08758	sp|P08758|ANXA5_HUMAN	7	25.30%	32224369.5	10	28.80%	49719677.8	-	-	NA	-	-	NA	1
P23142	sp|P23142|FBLN1_HUMAN	5	3.80%	1466724.6	2	3.80%	2459734.0	-	-	NA	-	-	NA	2
Q13162	sp|Q13162|PRDX4_HUMAN	9	35.10%	26078595.4	3	13.70%	6024437.6	-	-	NA	-	-	NA	3
P07437	sp|P07437|TBB5_HUMAN	6	14.60%	25703129.9	8	25.20%	55350672.9	-	-	NA	-	-	NA	4
P68104	sp|P68104|EF1A1_HUMAN	2	5.00%	3048791.5	13	22.10%	110698479.5	-	-	NA	-	-	NA	5
Q14315	sp|Q14315|FLNC_HUMAN	3	1.60%	5603017.7	3	1.70%	7215468.6	-	-	NA	-	-	NA	6
P63244	sp|P63244|RACK1_HUMAN	4	9.80%	11063905.3	6	19.20%	13987860.8	-	-	NA	-	-	NA	7
P22830	sp|P22830|HEMH_HUMAN	4	12.10%	5859705.7	2	6.40%	3433252.2	-	-	NA	-	-	NA	8
Q9NUJ1	sp|Q9NUJ1|ABHDA_HUMAN	9	23.50%	60338924.6	6	23.50%	31813404.5	-	-	NA	-	-	NA	9
Q07021	sp|Q07021|C1QBP_HUMAN	5	23.40%	12216562.9	7	30.50%	10561743.6	-	-	NA	-	-	NA	10
P08238	sp|P08238|HS90B_HUMAN	4	6.80%	5811808.5	8	11.20%	9429192.3	-	-	NA	-	-	NA	11
P58107	sp|P58107|EPIPL_HUMAN	3	6.30%	6624440.7	5	7.40%	7148889.4	-	-	NA	-	-	NA	12
Q5VTE0	sp|Q5VTE0|EF1A3_HUMAN	2	5.00%	3048791.5	13	22.10%	110698479.5	-	-	NA	-	-	NA	13
P02545	sp|P02545|LMNA_HUMAN	6	11.60%	10329164.5	15	22.30%	31423324.7	-	-	NA	-	-	NA	14
Q13813	sp|Q13813|SPTN1_HUMAN	5	2.30%	6727715.1	12	5.50%	21917267.4	-	-	NA	-	-	NA	15
Q14247	sp|Q14247|SRC8_HUMAN	2	5.60%	1329537.6	3	8.70%	2438124.6	-	-	NA	-	-	NA	16
P25705	sp|P25705|ATPA_HUMAN	4	9.90%	4471732.7	5	9.80%	9538660.3	-	-	NA	-	-	NA	17
O43707	sp|O43707|ACTN4_HUMAN	4	6.50%	3511264.1	10	15.10%	11877222.6	-	-	NA	-	-	NA	18
P31943	sp|P31943|HNRH1_HUMAN	2	7.60%	3393382.8	10	28.50%	52748142.7	-	-	NA	-	-	NA	19
Q9HCC0	sp|Q9HCC0|MCCB_HUMAN	9	21.10%	29536330	8	17.80%	13338068.2	-	-	NA	-	-	NA	20
P21333	sp|P21333|FLNA_HUMAN	4	2.50%	6342449.9	10	6.30%	15553661.0	-	-	NA	-	-	NA	21
P10909	sp|P10909|CLUS_HUMAN	5	12.50%	22295973.5	5	15.60%	19546062.9	-	-	NA	-	-	NA	22
P14625	sp|P14625|ENPL_HUMAN	2	3.20%	900774.7	2	3.70%	877245.4	-	-	NA	-	-	NA	23
Q96AY3	sp|Q96AY3|FKB10_HUMAN	3	7.20%	1492234.1	2	4.60%	1949469.7	-	-	NA	-	-	NA	24
P10809	sp|P10809|CH60_HUMAN	4	11.00%	12536801.8	12	27.90%	52881751.1	-	-	NA	-	-	NA	25
Q9BPW8	sp|Q9BPW8|NIPS1_HUMAN	3	7.70%	11926513.5	6	16.90%	18200505.3	-	-	NA	-	-	NA	26
P08865	sp|P08865|RSSA_HUMAN	2	9.50%	5730380.6	6	34.20%	32903795.3	-	-	NA	-	-	NA	27
P25398	sp|P25398|RS12_HUMAN	2	14.40%	2549587.9	2	14.40%	9099203.0	-	-	NA	-	-	NA	28
P48681	sp|P48681|NEST_HUMAN	5	3.60%	5302944	4	3.00%	3907017.0	-	-	NA	-	-	NA	29
Q9UFN0	sp|Q9UFN0|NPS3A_HUMAN	5	19.00%	33963288.3	4	19.00%	15507249.5	-	-	NA	-	-	NA	30
P27797	sp|P27797|CALR_HUMAN	3	8.20%	5224741.7	6	14.60%	9216025.1	-	-	NA	-	-	NA	31
Q71U36	sp|Q71U36|TBA1A_HUMAN	6	21.50%	29922465.5	7	23.70%	47789780.9	-	-	NA	-	-	NA	32
P37108	sp|P37108|SRP14_HUMAN	2	16.20%	8365172.2	3	18.40%	11313668.7	-	-	NA	-	-	NA	33
Q08380	sp|Q08380|LG3BP_HUMAN	3	6.30%	9561924.3	3	6.30%	7347583.9	-	-	NA	-	-	NA	34
P50454	sp|P50454|SERPH_HUMAN	3	9.10%	8744005.2	6	15.30%	21333166.9	-	-	NA	-	-	NA	35
P04792	sp|P04792|HSPB1_HUMAN	3	20.50%	23643601	13	70.70%	145325769.0	-	-	NA	-	-	NA	36
P38646	sp|P38646|GRP75_HUMAN	3	5.60%	5160367.1	15	25.90%	43724203.0	-	-	NA	-	-	NA	37
P31689	sp|P31689|DNJA1_HUMAN	4	9.80%	4520581.7	3	9.30%	7561207.7	-	-	NA	-	-	NA	38
P52272	sp|P52272|HNRPM_HUMAN	3	5.50%	3919805.0	7.0	12.90%	13392359.5	2.0	3.80%	1083129.3	-	-	NA	145
P60709	sp|P60709|ACTB_HUMAN	3	6.90%	9774197.8	6.0	10.40%	19952668.1	3.0	9.90%	7068840.1	-	-	NA	146
P63261	sp|P63261|ACTG_HUMAN	3	6.90%	9774197.8	6.0	10.40%	19952668.1	3.0	9.90%	7068840.1	-	-	NA	
P08670	sp|P08670|VIME_HUMAN	34	42.10%	537769480.8	39.0	46.60%	768408848.6	-	-	NA	3	7.70%	10182544.7	147
P0CG47	sp|P0CG47|UBB_HUMAN	2	32.80%	2952703.9	5.0	44.50%	31005435.5	-	-	NA	2	32.80%	1812965.3	148
P0CG48	sp|P0CG48|UBC_HUMAN	2	32.80%	2952703.9	5.0	44.70%	31005435.5	-	-	NA	2	32.80%	1812965.3	
P62979	sp|P62979|RS27A_HUMAN	2	16.00%	2952703.9	5.0	21.80%	31005435.5	-	-	NA	2	16.00%	1812965.3	
P62987	sp|P62987|RL40_HUMAN	2	19.50%	2952703.9	5.0	26.60%	31005435.5	-	-	NA	2	19.50%	1812965.3	
P07355	sp|P07355|ANXA2_HUMAN	8	26.50%	40210475.3	9.0	31.90%	42170467.6	-	-	NA	3	12.10%	9792524	149
P68366	sp|P68366|TBA4A_HUMAN	4	15.00%	23386565.8	7.0	22.30%	33379866.3	-	-	NA	3	11.60%	7964285.3	150
P11142	sp|P11142|HSP7C_HUMAN	6	10.80%	48964456.6	13.0	26.90%	47557589.7	-	-	NA	4	6.00%	12736150.3	151
P81605	sp|P81605|DCD_HUMAN	3	37.30%	25006545.0	3.0	27.30%	31143502.9	-	-	NA	2	20.90%	8753027	152
P11498	sp|P11498|PYC_HUMAN	3	3.40%	6213999.0	4.0	4.80%	6548114.5	12.0	14.30%	23340759.8	2	2.10%	1944618.1	153
Q9BQE3	sp|Q9BQE3|TBA1C_HUMAN	6	20.00%	43001332.3	7.0	22.30%	58390795.5	2.0	7.60%	3543524.4	3	11.60%	11061543.9	154
P11021	sp|P11021|BIP_HUMAN	44	42.00%	496331015.8	44.0	40.70%	255231121.8	18.0	25.70%	41963583.4	23	26.00%	66461412.3	155
P68363	sp|P68363|TBA1B_HUMAN	6	21.70%	40454048.5	8.0	26.80%	59869925.7	2.0	7.50%	3543524.4	3	11.50%	11061543.9	156
Q9Y2S7	sp|Q9Y2S7|PDIP2_HUMAN	5	13.90%	17317387.1	7.0	20.40%	26643094.9	2.0	6.50%	2832720.1	2	7.30%	4465018.1	157
O43813	sp|O43813|LANC1_HUMAN	12	25.60%	33776781.2	5.0	18.80%	26892019.3	2.0	5.80%	10638246	6	14.00%	19251428.8	158
P0DMV8	sp|P0DMV8|HS71A_HUMAN	15	25.30%	117963214.3	26.0	39.50%	262536450.6	7.0	12.50%	12959378.3	16	29.20%	94861648.5	159
P0DMV9	sp|P0DMV9|HS71B_HUMAN	15	25.30%	117963214.3	26.0	39.50%	262536450.6	7.0	12.50%	12959378.3	16	29.20%	94861648.5	
P34931	sp|P34931|HS71L_HUMAN	8	14.80%	105896748.0	10.0	16.50%	140359610.2	4.0	6.40%	11869670.4	10	16.50%	61597914.3	160
